# Resilience, innovation and collapse of settlement networks in later Bronze Age Europe: New survey data from the southern Carpathian Basin

**DOI:** 10.1371/journal.pone.0288750

**Published:** 2023-11-10

**Authors:** Barry Molloy, Dragan Jovanović, Caroline Bruyere, Marta Estanqueiro, Miroslav Birclin, Lidija Milašinović, Aleksandar Šalamon, Kristina Penezić, Christopher Bronk Ramsey, Darja Grosman

**Affiliations:** 1 UCD School of Archaeology, University College Dublin, Belfield, Dublin, Ireland; 2 City Museum Vršac, Vršac, Serbia; 3 Centre for Studies in Archaeology, Arts and Heritage Sciences (CEAACP), University of Coimbra, Largo da Porta Férrea, Coimbra, Portugal; 4 National Museum Pančevo, Pančevo, Serbia; 5 National Museum Kikinda, Kikinda, Serbia; 6 National Museum Zrenjanin, Zrenjanin, Serbia; 7 Biosense Institute, University of Novi Sad, Novi Sad, Serbia; 8 School of Archaeology, University of Oxford, Oxford, United Kingdom; 9 Darja Grosman, Department of Archaeology, Faculty of Philosophy, University of Ljubljana, Ljubljana, Slovenia; Austrian Academy of Sciences, AUSTRIA

## Abstract

Societies of the later Early to Middle Bronze Age (ca. 2200–1600 BC) in the Carpathian Basin exhibited complex, hierarchical and regionally influential socio-political organisation that came to an abrupt end in the 16^th^ century BC. Considered a collapse by some, this change was characterised by abandonment of virtually all central places / nodes in settlement networks. Until recently, the complexity that characterised the period was believed to have substantially diminished alongside depopulation. This model was reinforced by a combination of the loss of established external networks and low-resolution knowledge of where and how people lived in the first stages of the Late Bronze Age (between 1600 and 1200 BC). We contest the idea of a diminished Late Bronze Age and argue that a fully opposite trajectory can be identified–increased scale, complexity and density in settlement systems and intensification of long-distance networks. We present results of a settlement survey in the southern Pannonian Plain using remote and pedestrian prospection, augmented by small-scale excavations. New absolute dates are used to define the occupational history of sites dating primarily between 1500–1200 BC. We argue that climate change played a substantial role in in the transformation of settlement networks, creating a particular ecological niche enabling societies to thrive. New and specific forms of landscape exploitation developed that were characterised by proximity to wetlands and minor watercourses. In this context, the largest monuments of Bronze Age Europe were created and inhabited. In considering the origins and demise of these megasites and related settlements, we provide a new model for Late Bronze Age societies in the Carpathian Basin and their regional relevance.

## Introduction

**Fig 1:**
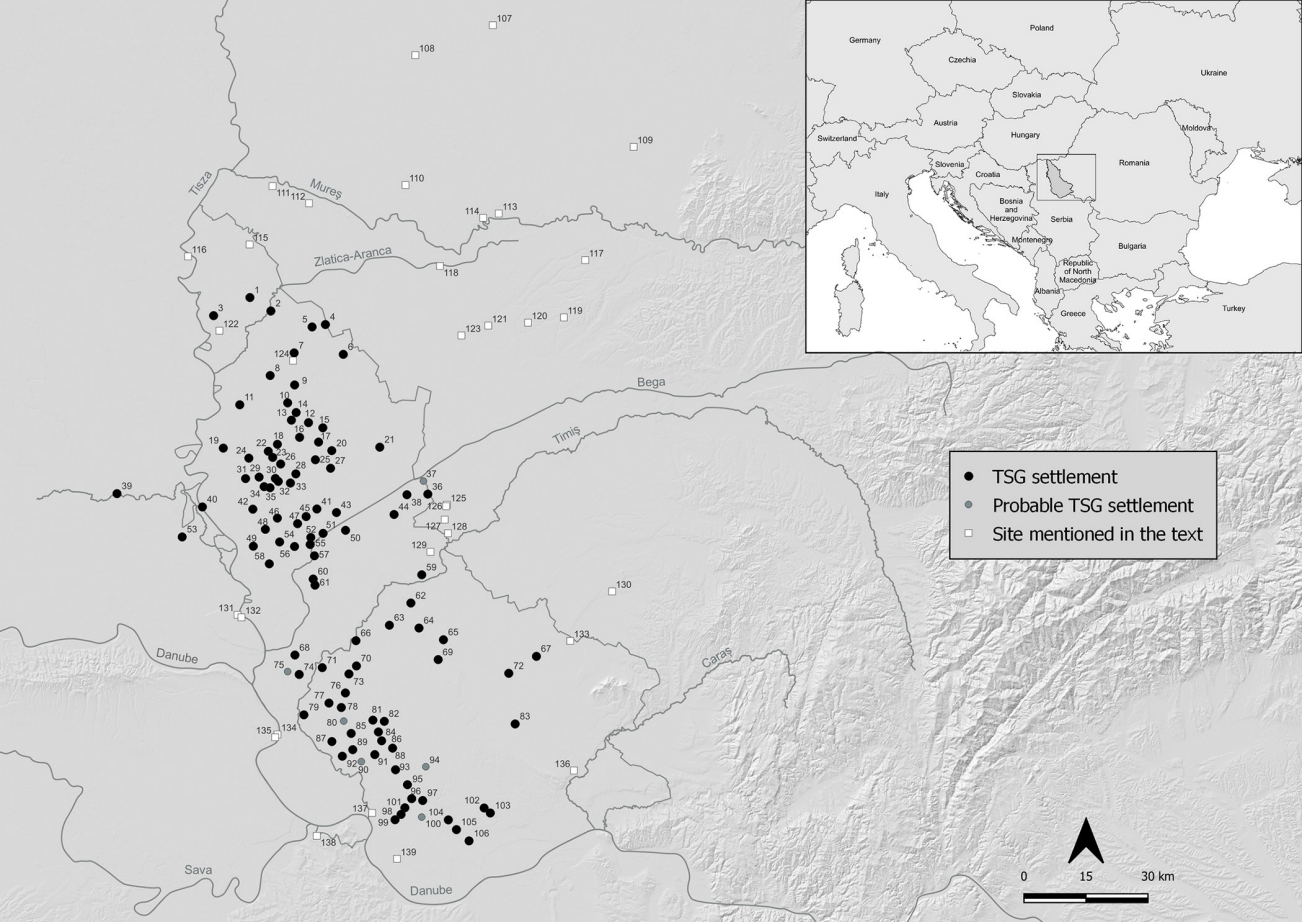
Location map of study are and sites mentioned in the text. Black circles: TSG sites; Grey circles: probable TSG sites (not surveyed); white squares: TSG-like sites. 5 = Mokrin; 7 = Gradište-Iđoš; 11 = Novo Miloševo 4; 18 = Novo Miloševo; 25 = Bašaid; 27 = Torda 2; 29 = Kumane 3; 34 = Melenci 2; 39 = Turija-Gradište 41 = Banatski Dvor; 46 = Melenci; 50 = Žitište; 52 = Jankov Most; 65 = Dobrica; 73 = Idvor; 76 = Sakule; 84 = Crepaja; 92 = Glogonj; 93 = Kačarevo 2; 95 = Pančevo; 96 = Pančevo 2-Stari Tamiš; 97 = Dolovo; 102 = Mramorak; 103 = Mramorak 2; 104 = Bavanište; 107 = Újkígyós-Örök-dűlő; 108 = Orosháza Nagytatársánc; 109 = Sântana-Cetatea Veche; 110 = Csanádpalota-Foldvar; 111 = Klárafalva-Hajdova; 112 = Kiszombor-"Juhos Miklos tanya = Uj Elet Tsz"; 113 = Pecica in Vii; 114 = Pecica Şanţul Mare; 115 = Rabe-Anka Siget; 116 = Kanjiža-Ribarski Trg; 117 = Sagu Sit A1_1; 118 = Periam-Movila Santului; 119 = Corneşti-Iarcuri; 120 = Carani; 121 = Satchinez-Râtul Popii; 122 = Ostojićevo-Stari Vinogradi; 123 = Biled-Câmpia Arsă; 124 = Budžak-Livade; 125 = Foeni-Gomila Lupului II; 126 = Foeni-Gomila Lupului I; 127 = Foeni-Cimitirul Ortodox; 128 = Cruceni-Modusi Ut; 129 = Jaša Tomić; 130 = Butin; 131 = Mošorin-Feudvar; 132 = Mošorin-Stubarlija; 133 = Vatin-Bela Bara; 134 = Belegiš-Šančine; 135 = Belegiš-Stojića Gumno; 136 = Orešac-Židovar; 137 = Pančevo-Najeva Ciglana; 138 = Karaburma; 139 = Omoljica–Zlatica. See Table 2 for other TSG sites numbered in this figure (Map by Marta Estanqueiro; basemap hillshade derived from ALOS DSM AW3D30, reprinted from https://www.eorcJaxaJp/ALOS/en/dataset/aw3d30/aw3d30_e.htm under a CC BY license, with permission from JAXA -Japan Aerospace Exploration Agency, original copyright 2023).

The mid-second millennium BC was a time of major transformation in Europe, considered to be a key turning point for prehistoric societies [1]. Metal, which had long served as a prestige resource was increasingly used for utilitarian objects, transforming communities into truly metal-using societies that were dependent on it in daily life and, at a more macro-scale, its consumption fuelled competitive political economies [2–4]. Societies of the Carpathian Basin have long been regarded as creative driving forces behind the cultural, exchange and political networks that defined the globalised connections of this time [5–10]. Some of the most important overland and river navigational corridors in Europe intersect in the south of that region, particularly the Danube and Tisza rivers. Bronze Age research there has primarily focussed on societies centred around tell settlements of the later Early and Middle Bronze Age (ca. 2100–1600 BC) [11]. At that time, well-resourced communities created a wealth of material culture indicative of complex, hierarchical societies. With the abandonment of tells and surrounding sites in the 16^th^ century BC, this social order came to an abrupt end. After this, when the kingdoms and empires of the Mediterranean reached their apogee between 1500–1200 BC and many European polities grew in complexity, we know remarkably little about what was happening in the once-central Carpathian Basin (Fig 1).

It has been argued the area was marginalised in political and economic networks of Europe and the Mediterranean during the Late Bronze Age (LBA) [12–14]. However, we argue in this paper that a new internally highly-connected and externally well-networked society emerged after 1600 BC, characterised by a dense and hierarchically organised complex of enclosed sites. Located in the south Pannonian Plain area of the Carpathian Basin, these sites were commonly monumental in scale, ranging from small sites of 5–10 hectares, through larger ones of many 10’s of hectares up to the largest site which exceeded 1,750 hectares of space encircled by 33km of ditches and ramparts [15–19]. We aim to explain how this dense and prosperous network came into existence in the 16^th^ century and why it collapsed in the 13^th^ century BC. We contend that, alongside the Aegean and Po Valley, this newly identified lower Pannonian network was one of the major cultural centres of southern Europe, exerting regional scale influences across the continent and into the Mediterranean.

We focus on the southeast Pannonian Plain due to the quality and quantity of new data from recent excavation programmes and our survey, but also because this area was exceptionally (for the region) densely occupied and prosperous in the LBA. We include sites from the area in and around the historical province of Banat (spanning parts of today’s Hungary, Romania and Serbia), correlating with the hinterlands of the river Mureș in the north, the river Tisza in the west, the river Danube in the south and fringe of the Transylvanian uplands to the east (Fig 1). Characterised by common features in settlement design, mortuary practice, metalwork style and ceramic forms (particularly of the Belegiš tradition), shared elements of social reproduction common across a wide area indicate the presence of a socio-political entity that we call the *lower Pannonian network*. This was constituted by one to several multi-local polities. Each included multiple settlements and shared a core macroculture. To date research excavation projects have focussed on large and complex sites that served as central places. While this has begun to provide some insights into the social world in which these large sites found meaning, we contextualise these megasites alongside the heterogenous array of smaller sites that make up an interlinked social landscape [15]. Harding [15] introduced the term megafort to refer to the massive enclosed LBA sites of this area. In this paper, we define megasite or megafort as an individual, built enclosure (i.e. not a composite of multi-local related constructions that are not physically linked) that is substantially larger than typical across all periods and places in prehistoric Europe. We suggest that an enclosed space of greater than 75 hectares and possessing monumentality vis-à-vis a substantial ditch and / or rampart is contextually meaningful for the LBA of this region, even if not all sites of this scale had identical functions.

Our fieldwork has identified over 100 new sites in a part of this region. They are located in the hinterlands of the Tisza river and so we term them the Tisza Site Group (TSG). The TSG is a network of highly similar sites set within an area of ca. 8,000 km^2^, roughly 140km north-south and 80km east-west (S1 File). They are aligned along a broadly north-south axis east of a river corridor formed by the River Tisza and a north-south stretch of the River Danube. The results of our survey provide a basic model for settlement frequency, density, organisation and size. The distribution and locational choices make it clear that TSG sites occupied a strategic location along important arterial river routes that connected them to each other, continental Europe and onwards to the Adriatic, Black and east Mediterranean seas [20]. We present results of remote prospection, pedestrian survey, targeted excavations and new absolute chronological research on this TSG cluster and use this to characterise the lower Pannonian network in its regional and European context.

### The landscape, environmental and climate context of the southern Pannonian Plain 2000–1000 BC

The Carpathian Basin is defined as a bowl with a mountainous periphery and an exceptionally flat interior (the Pannonian Plain) punctuated by very rare and isolated hills or mountains (Fig 1). Features such as marshes appear to have played a role in shaping settlement locations in prehistory, but the massive rivers of the plain were even more formative. They are broad all year round but when in flood they become vast watery zones creating seasonal no-man’s land spaces. These rivers possess a ‘“yin/yang” character, in that they function both to hinder and to facilitate movement’, enabling societies to shape, interact and sustain large multi-local social aggregates or perhaps polities [20].

To better understand the changing environmental context of settlement between the MBA and LBA, and also the eventual abandonment of LBA settlements, data for paleoclimatic conditions and landscape management are revealing (Table 1). Studies from areas adjacent to the TSG present a consistent picture. Demény et al.’s [21] study of a variety of climate proxies provide the highest resolution records and are supported by other speleothem data from Ascunsă, Poleva and Urşilor caves, an ice core from Scărișoara cave, and sediment cores from Lake Brazi and Late Ighiel in western Romania [22–26]. Most LBA 1 and 2 datapoints indicating wetness levels are below those indicating the wettest MBA conditions, suggesting consistently higher rainfall. The warmest peaks of LBA 1 and 2 are just above the lowest points for the MBA in Demény et al.’s model, suggesting relatively cooler LBA conditions.

**Table 1:** General trends in climate proxy data for the southern Pannonian Plain. This table is based on data from Demény et al. 2019 and supported by data from Drăguşin et al. 2014. Demeny et al. is based primarily on data from Trio Cave and Dragusin et al. focus on Ascunsă, Poleva, and Ursilor caves. These are located ca. 200 km to the west-north-west and east-north-east of the centre of the TSG network respectively. All conditions in the figure are relative to those of 2000–1900 BC when this timeline begins. As the dating of changes is broadly construed within century blocks, when peaks occur within a block, the following trajectory is opposite to the preceding one, i.e. if preceded by cooling then following the peak, prevailing conditions shifted to warming.

Relative Chronology	Absolute chronology	Temperature	Precipitation	TREND
MBA 1	2000–1900 BC	Cold	Wet	
MBA 2	1900–1800 BC	Warming	Increasing aridity	**Warm and dry but vacillating** Steadily decreasing temperature and increasing precipitation
	1800–1700 BC	Warming	Gradually lowering aridity	
MBA 3	1700–1600 BC	Warm peak	Increasing wetness	
LBA 1	1600–1500 BC	Brief but coldest peak	Increasing wetness	Increasing stability
	1500–1400 BC	Begins with warm peak then gradual cooling	Drying, but wet	**Cold and wet** Steadily increasing temperature and decreasing precipitation
LBA 2	1400–1300 BC	Gradual and slight cold peak, colder	Stable but wet	
	1300–1200 BC	Warming toward slight warm peak	Increasing wetness	
LBA 3	1200–1100 BC	Slight cold peak	Drying	**Warm and dry** Slowing of rivers, rising aridity
	1100–1000 BC	Warmer	Drier	

When the number and complexity of Middle Bronze Age (MBA) tells were increasing (broadly 1950–1750 BC), palaeoclimate data indicate comparatively warm and dry–but quite vacillating–conditions. Data suggest a steady increase in wetness and falling average temperatures from the 17^th^ to mid-16^th^ century BC [21]. The same data indicate the onset of less frequently fluctuating conditions in the 16^th^ century BC. This 16^th^ century BC change horizon was sufficiently pronounced for Kern et al. [27] to posit a 3.5ka climate event occurred across east-central and south-eastern Europe. During the LBA 1 and 2 phases the climate appears relatively cooler and wetter than the growth period of MBA tells. During the 14^th^ to 13^th^ centuries BC rainfall decreased alongside rising temperatures but with relatively stable hydroclimatic conditions overall. This trend accelerates ca. 1250 BC, with an increasingly arid climate and rising temperatures for ca. two centuries, bringing conditions close to those of the growth period of MBA tells [21, 23]. French’s [28] geoarchaeological study of the nearby Benta Valley suggests this 13^th^-12^th^ century aridity played a role in the slowing of rivers in parts of the plain.

Hydroclimate and temperature observations are primarily derived from speleothem analyses, with the Hungarian data possibly biased towards reflecting wintertime conditions. There is less clarity on the seasonality of change (greater summer or winter rainfall patterns) in the Romanian data. In many ecosystems, winter rainfall provides an opportunity for soils to recharge and remain productive in annual cycles, particularly when comparatively dry summers are experienced [21, 23]. This may have had benefits for agricultural productivity.

Regarding vegetation history of the Pannonian Plain, there are few data relevant to the Bronze Age, largely a consequence of poor pollen preservation. Gumnior et al.’s [18] study of the site and hinterlands of Corneşti Iarcuri, a LBA mega-fort of the lower Pannonian network in Western Romania, shows diverse species and relatively high tree-coverage, with arboreal pollen accounting for 38–46% of pollen from the settlement and 60–65% of pollen from nearby lake sediments. Notably, anthropogenic pollen markers are reduced after 1200 BC, suggesting a shift in landscape exploitation [18].

Working at a multi-decadal scale, we can identify a general correlation between the MBA / LBA transition and changing climatic conditions. This provides a context, rather than a cause, for social transformations given that societies existed at the interface between environmental conditions and social expectations of landscape use [22, 29–36]. The exceptionally flat landscape of the southern Pannonian Plain with its frequent and massive waterways means that ecosystems were finely balanced relative to hydroclimatic conditions. For this reason, socially specific modes of land use would have been vulnerable to shifts in the predictability and levels of rainfall and their impact on the water table.

### Material culture and the relative chronology

**Fig 2:**
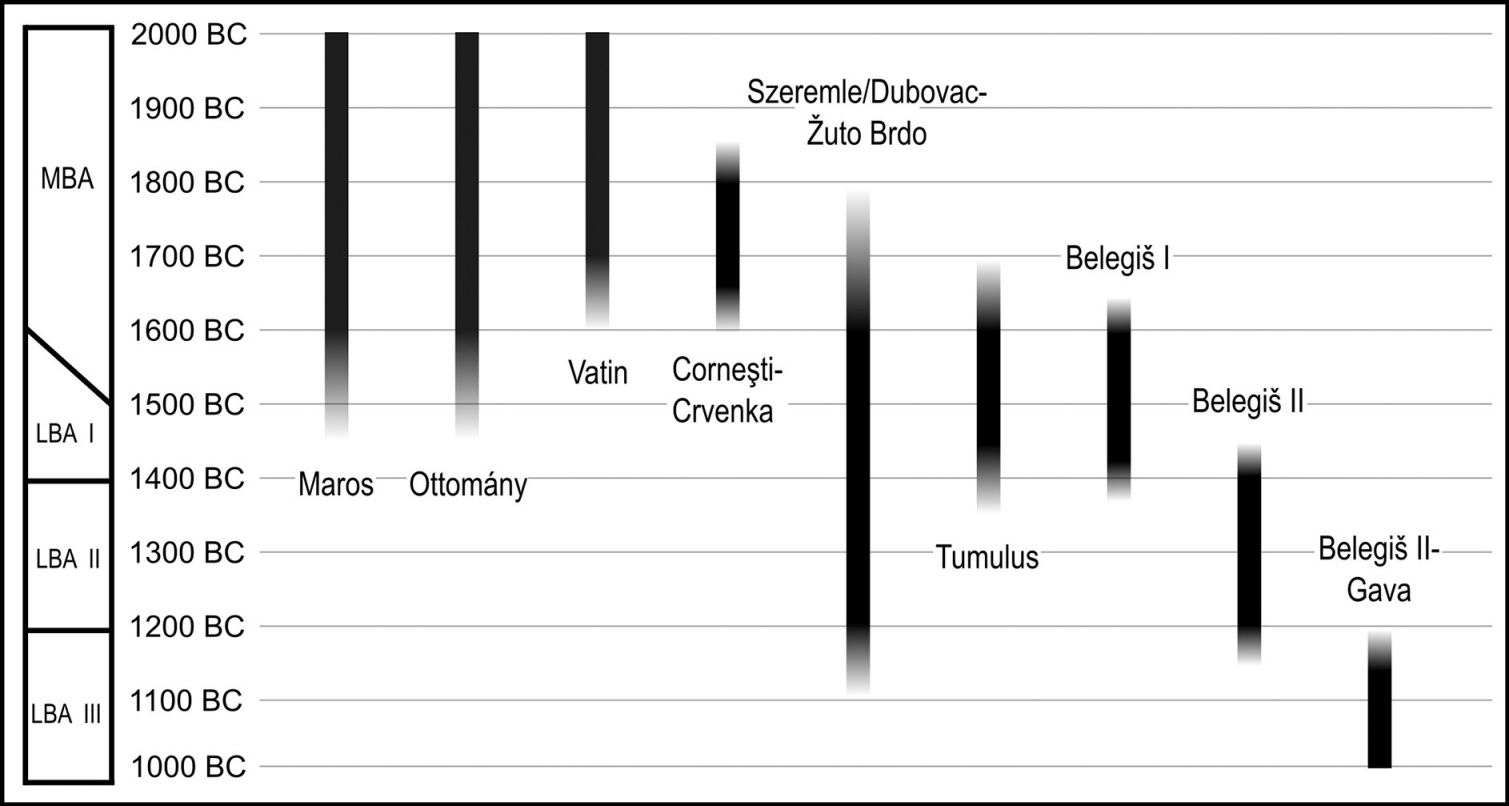
Relative ceramic chronologies commonly used in the study area (Drawn by Caroline Bruyere).

The following brief overview of ceramic relative chronologies introduces relevant terminology (Fig 2). The MBA in the south Pannonian Plain was a complex patchwork of material culture styles. At the peak of tell habitation, sites located south of the river Timiş primarily consumed variants of Vatin style pottery, for example at the tells of Židovar and Feudvar and the settlement at Vatin. Along the Mureş river, tells such as Pecica, Periam and Kiszombor were dominated by the Maros style of pottery alongside a minority of the Otomani-Füzesabonystyle that was dominant farther north [19, 37, 38]. Recent work favours the term Cornești-Crvenka style for pottery of the latest phase of the MBA, though Vatin style can also be found in literature of recent decades [38–40]. On many tells a combination of styles can be found in various proportions, though minority styles tend to be labelled as imports. There remains debate about boundaries and relevance of these groups, but relevant literature uses these conventions.

The long-standing Tumulus Culture invasion model was introduced to explain the adoption of new material culture styles from the 16^th^ century BC, prompting arguments for a causal relationship between inward migration and the fall of the tells, an idea now out of vogue [5, 41–46]. This model was based in part on the development of distinctive metalwork of the so-called “Koszider horizon” (ca. 1600–1450 BC) along with the “Tumulus culture” or HGK (Hügelgräber-Kultur) pottery style of the same general date range [44, 47]. Recent work demonstrates that the introduction of these was asynchronous, gradual and embedded within local craft and societal dynamics [48]. The term Koszider is common in the literature but problematic, even unhelpful today, as it has been alternatively linked to hoarding practices, metalwork styles or a period of time, with its relevance for understanding past phenomena shifting according to how the term itself is used [49]. We prefer tumulus culture (with a small c) as a typo-chronological term when dealing with the ceramic styles. Kapuran argues that the impetus to use tumulus culture ceramics was introduced into the area of the lower Pannonian network via two streams from the north across the Mureș and from the south along the Danube [47]. This is based on a bias in distribution of pottery of this style in the north and south, and less-so the middle, of Banat. This is seen for example in the published assemblage from Gradište Iđoš and from its cemetery at Budžak Livade in the north and the cemetery of Karaburma in the south [50–53].

In the Hungarian Danube region, Transdanubian Encrusted Ware develops during the MBA and the theory remains that migration brought this style into the Serbian Danube area, having developed into the related encrusted Szeremle style and influenced the Dubovac-Žuto Brdo style. The Szeremle style developed along the north-south stretch of the Danube corridor and Dubovac-Žuto Brdo style along the same river after its turn to an easterly flow. There are close similarities to both styles during the LBA with regard to motifs, decorative schema and vessel shapes, but the earliest phases of each style are traced to the MBA when they initially draw on related but distinct local traditions. Sidestepping discussion of minor differences for our purposes here during the LBA phase we consider them as a broad stylistic family under the acronym SDŽB. The consumption of SDŽB was focussed on the Danube but it was consumed commonly in the southern reaches of the lower Pannonian network [47, 54, 55].

In general, the southward spread of the consumption of tumulus culture and encrusted ware styles signal mobility. While we may only speculate on whether this was people, things or ideas on the move, it correlates with a disruptive phase at the end of the MBA. Their introduction alongside other ceramic styles is relevant for understanding the late 17^th^ to 16^th^ centuries BC as a pivotal time of reorganisation across the southern plain [54].

In the LBA, Belegiš I ceramics emerge as a stylistic tradition ca. 1600 BC and were related to the Vatin and Maros traditions [55–57]. The second phase, Belegiš II emerged in the late 15^th^ century and is closely related to pre-Gava styles in Hungary [38, 56, 58–61]. These ceramics were consumed throughout the lower Pannonian network, but also as far east as the foothills of the Transylvanian plateau and past the Sava-Drava confluence in the west. They are found less commonly south of the Danube prior to 1200 BC [62]. Notably, the co-occurrence of Belegiš I and SDŽB at TSG settlements, and the common use of incised and / or encrusted decoration, suggests our division between the two, based primarily on cemetery assemblages, is perhaps sharper than would have been viewed in prehistory. When the Belegiš II pottery style emerged, many features such as hanging garland decoration, cylindrical necks with everted rims and four equispaced protomes derived from the Belegiš I style. These features were shared also in a modified format on later SDŽB pottery, demonstrating entanglement of concepts even when broader decorative techniques were adhered to.

Beyond serving as typo-chronological markers, these trends show that the TSG communities were emerging when distinctive pottery styles of Belegiš, SDŽB and tumulus cultural traditions were consumed together. This demonstrated incorporation of the preceding Vatin and Maros traditions, of influences from the Danube and Mureș hinterlands, as well as of styles from non-adjacent areas from farther to the north. The image gained from earlier LBA craftwork / domestic wares is that communities were permeable enough to accommodate influences from varied networks. By the onset of the LBA 2 period ca. 1400 BC, there was increasingly widespread use of Belegiš II style, suggesting that material culture had become a vehicle for cultural integration, bucking a long-standing trend for it to be used to embody difference. Research in the Carpathian Basin and Balkans has often been dominated by culture history / typo-chronological frameworks. It is important to avoid slippage between speaking of ceramic groups as material culture categories with spatial and temporal relevance and the people who made the pots [63, 64]. Ceramic groups cannot viewed as direct proxies for ethnic or cultural identity and political boundaries, but represent expressions of choice and knowledge [65]. Nonetheless, ceramic traditions are linked to geographic spaces and between shape, decoration and function they structured the experiences / habitus of people using them. They were one among many features of daily life that embodied difference between groups. It is important because societies of the Carpathian Basin during the MBA and LBA produced a rich array of ceramic traditions. In aiming to explain, as well as describe, why distribution patterns change in temporal and geographic space we must account for choices and their implications for the materiality of communities. Pots may not equal people, but in this area they could and did express difference.

### The middle Bronze age social and settlement background

Many EBA-MBA tell sites in the Pannonian Plain have been known since the earliest days of archaeology in the 19^th^ century, yet few have been systematically excavated and published, particularly in the east and south Pannonian Plain [37, 48, 66–70]. A sufficient number has been investigated to reveal there was no single foundation horizon. The origins of Bronze Age settlements are staggered at existing (some had been intermittently occupied since the Neolithic) or newly founded tells [66, 71, 72]. This site-type became increasingly common from the later third millennium BC to peak in the middle of the first half of the second millennium BC with respect to site numbers and density of occupation [73]. In the Carpathian Basin 188 MBA “proper tell sites”–defined by Gogâltan [66] as sites with over 1m of continuous occupation stratigraphy–are known. As definitions for what constitutes a tell may be debated, this can be seen as a minimum number.

Tells were a feature common to most, not all, MBA communities in the Pannonian Plain. There remained diversity in material culture and associated elements of death- and lifeways in different parts of the plain [12, 54, 57, 74]. While tells constituted a common settlement form, there was regional diversity in how they were integrated into settlement networks and organised internally [66, 71, 75]. Kienlin emphasises that Bronze Age tell sites represent a conceptual continuity with the Neolithic with regard to use of space and managing this landscape [76]. In essence, the commonality of tell societies lies in their sustained physical and ideological attachment to place. As we discuss below, this frames a sharp discontinuity in LBA ideologies of settlement and landscape.

It has been argued that a three-tier hierarchy existed between settlements in the Pecica polity, with Pecica-Şanţul Mare serving as a primary centre, other tells as secondary centres and smaller, commonly flat, settlements being third in the hierarchical order. In this model, primary centres were locales of craft production, limited centralisation of surplus produce and feasting in the east Pannonian Plain [37, 77]. It is evident that this model of hierarchical relations between tell and flat settlements is not tenable in all areas of the plain [12, 57, 71, 72, 75, 76]. A dearth of systematic excavation at flat settlements, and even the flat components of settlements surrounding tells, creates a bias in the data available to model hierarchies and networks [66, 75]. Current evidence indicates that not all MBA sites were equal in status, longevity, size or density and so a hierarchy of places was evidently recognised in the past [37].

A terminal phase between ca. 1600–1500 calBC is identified at almost all tells and many flat settlements with absolute dates throughout the Pannonian Plain, after which a widespread abandonment horizon has been identified [73]. This was for a long time considered to have been a regional scale collapse, though recent research demonstrates that this transformation was complex, staggered over a century, and that a transition rather than schism can be observed in some areas [19, 66, 71, 76, 78]. As we argue below, new evidence from LBA settlements indicates much of this change was proactive adaptation and innovation in the context of changing political conditions that undermined the systemic logic and power dynamics of southern MBA regimes.

In the northern Pannonian Plain Fischl et al. [12] detect a shift from dense and complex settlement patterns centred around MBA tells to less complex, open LBA settlements, whereas in the Benta Valley, despite a reduction of site numbers and estimated population level, hierarchical ordering of settlements survives into the LBA [69, 79]. In the southeast Pannonian Plain, decline can be detected between 1700 and 1550 BC, with some settlements being abandoned and the density of activity at previously prosperous sites diminishing [77, 80]. Changes in the 16^th^ century were not sudden, but were socially far-reaching, irreversible, spatially extensive and included virtually all central sites / primary centres / core network nodes and their cemeteries. This unfolded in less than a century–a timeframe consistent with anthropological models of collapse [36, 81, 82]. In our view, what collapsed was the political / ideological regimes, and widespread participation in these. Despite these fundamental shifts in how people organised themselves in settlements and communities, features of daily life such as craft, rituals and diet reveal resilience in macrocultural traits of the general populace. As Johnson [81] puts it “ordinary citizens tend to muddle through transitions and adapt to a new normal,” while those exploiting or directly profiting from higher-order organisational infrastructures tend to be destabilised or disenfranchised.

**Fig 3:**
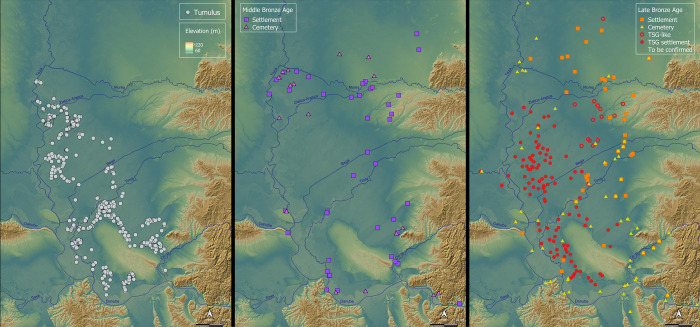
Tumuli (Left) MBA (Centre) and LBA (Right) settlements and cemeteries in topographic perspective (Drawn by Marta Estanqueiro and Caroline Bruyére; basemap hillshade reprinted from https://www.eorcJaxaJp/ALOS/en/dataset/aw3d30/aw3d30_e.htm under a CC BY license, with permission from JAXA -Japan Aerospace Exploration Agency, original copyright 2023).

Around the northern frontier of the area to be occupied by the lower Pannonian network, abandonment of central sites and cemeteries of the Pecica polity occurred between 1600 and 1500 BC [19, 48]. The tells at Mošorin-Feudvar and Belegiš (on the western TSG limit), Židovar (southern limit) and Foeni along with the tell-like settlements of Vatin (east and south-east limit) and Pančevo-Najeva Ciglana and Zlatica–Omoljica east and south-west limit) will be discussed (Fig 1). It is immediately obvious from Fig 3 that 1) these important MBA sites are located on the margin of the area to become densely occupied in the LBA and 2) few MBA sites have been identified within that zone. On current evidence, the dividing line between settlements clustering around the perimeter and clustering within the core of the TSG area is set at ca. 1600–1500 BC. An overview of the abandonment of the above listed sites provides a context to the emergence of LBA sites and helps identify the nature of resilience, transformation and innovation as part of this societal reorganisation.

#### The Pecica polity

The tell of Pecica (Pecica-Șanțul Mare) lies on the northern bank of the Mureș river. It has been investigated since the 19^th^ century and the results of successive excavations identify the site as a central place in its local settlement network [66, 68, 77, 83, 84]. O’Shea [37] considers Pecica to lie at the heart of a ‘Pecica polity’ spreading over an area of 4,000sq KM encompassing the middle and lower stretches of the River Mureș and its confluence with the Tisza. This is immediately north of the TSG area but there is no spatial overlap between these networks of sites. This polity is said to incorporate secondary centres located 50 and 55km away at Klárafalva Hajdova and Rábé Anke-sziget respectively, as well as many flat settlements [77], though this model is debated [85]. A further tell on the opposite (west) bank of the Tisza from Rábé –Anka-sziget at Kanjiža-Ribarski trg may also have served as a secondary centre, though only small test excavations have taken place [86]. Other tell settlements occur in the hinterlands of the Mureș to the east and north of Pecica but are not discussed here as we are focussed on those bordering the TSG [80].

The development of centrality at Pecica has been viewed as a process embroiled in conflict as hegemony and autonomy were negotiated between sites in this network. The “Fluorescent period” at Pecica, when substantial building works and dense activity were defined on the tell, lasted from ca. 1850 to 1680 calBC [77]. After this, most flat settlements and cemeteries in the lower Mureș zone of the Pecica polity were abandoned and activity at Pecica itself became considerably sparser until its final abandonment before 1545 calBC. Based on relative dating of pottery, Klárafalva Hajdova, Kanjiža-Ribarski trg and Rábé Ankasziget were abandoned around this time, though absolute dates from coring at the latter are no later than 1600 calBC [48]. The MBA cemetery of Ostojićevo declined in use in the 18^th^ century BC but experienced a brief upswing in activity from 1600–1500 BC before being abandoned for over a century [48]. Ultimately, the Maros group had experienced declining population levels along with site abandonments since the 17^th^ century, a cycle that was complete by the mid-16^th^ century according to absolute dates from excavations [73]. These abandonments and the “terminal phase” at Pecica were contemporary to the swing towards wetter and cooler weather in palaeoclimate records in the mid-17^th^ to mid-16^th^ centuries BC (Table 1).

There was clear continuity of occupation in the surrounding area with new and important centres established in the mid-second millennium BC. The role of monumental central places continued but changed in form. Surface finds of LBA ceramics from the nearby site of Pecica in Vii suggest continuity near to the Pecica tell. Although the size of this site remains undefined, it may extend up to 100 ha in the LBA [87]. Within a ca. 30km radius of Pecica the megaforts of Csanádpalota (460ha) and Sântana Cetatea Veche (130ha) were constructed soon after the tell was abandoned [88–91]. Nearby, the extensively excavated unenclosed flat settlement of Șagu was a new foundation of the 16^th^ century BC [19, 38, 92]. Its relatively small size for an LBA site (22 ha) and lack of boundary ditch indicate it was probably not a high status site. The extensive evidence for metalworking there indicates a shift in ideology, control and status of metalworking and metalworkers in the MBA-LBA transition [4].

#### Mošorin-Feudvar

Looking beyond this site network in the north, fieldwork has also been conducted at the western limit of the TSG on the west bank of the Tisza at the Titel Plateau. The site of Mošorin-Feudvar was excavated by Hänsel and Medović in the late 1980’s, though publication has been partial [70, 93]. Falkenstein has conducted intensive surface survey and coring on the surrounding plateau, with results published along with some details from the excavations at Mošorin-Feudvar [94, 95]. The tell was established during the EBA at some point before 2000 BC, based on relative chronology of excavated strata. In the 17^th^ century and 16^th^ centuries BC, a notable increase in interaction with people making Maros pottery is attested. Consumption of late Maros and Otomani-Füzesabony ceramics (sometimes difficult to distinguish) is initially in a minority, but in the early 16^th^ century they briefly become dominant, while the previously dominant Vatin style ceramics continue as a minority component. The timing of the introduction of influences from Maros group ceramics correlates with abandonment of sites in the Pecica polity, including Pecica itself.

There are hints of conflict in the changes in settlement patterns observed around the Titel Plateau. Virtually all satellite settlements were abandoned ca. 1600 BC, around the time of a brief period of growth and fortification at Mošorin-Feudvar itself [70]. This tell was occupied into the 16^th^ century, yet exceptionally few sherds of Belegiš I or tumulus culture forms have been recovered there despite this pottery being common in sites in the surrounding area.

The tell was abandoned before the end of the 16^th^ century BC and intensive pedestrian survey shows that settlement thereafter spread across the 8,197ha Titel plateau. This reveals a shift from intensive to extensive patterns of settlement and land-use. The pottery used changes only then, when SDŽB and Belegiš families are adopted. A cemetery on the plateau at Stubarlija was established and remained in use until ca. 1200 BC. The tell at Mošorin-Feudvar was reoccupied in the 12^th^ century BC, based on absolute dates from the excavation and dating of pottery styles by Tasić and Falkenstein which are alternatively termed Belegiš IIb, Belegiš II-Gava or Belegiš III [56, 94].

Defining conflicts through pottery is a precarious affair as it risks equating pots with people, but the timing of changes in consumption patterns at Mošorin-Feudvar is important. Maros pottery was introduced during a period of decline in the core area of its manufacture and at a time when the newly emerging Belegiš style was excluded from the tell settlement. Following the abandonment of the central site, the dominant ceramic types switched and new settlements and a new cemetery were established. Falkenstein et al. [70] argue for a complete change of population during this upheaval. An argument for changes in hegemonic practices and elite lineages might well be seen in these marked changes in pottery the communities found it socially appropriate to use, with new rulers being more receptive to immigration and / or accommodating some social conventions of neighbouring groups. Other changes included the marginalisation of the tell as a focus of activity after it had long-served as a centre of local power. As will be discussed below, the extensification of land-use seen on the plateau is a hallmark of LBA conventions in the TSG area, even if a large enclosure is missing in this naturally defined space.

There is also a Bronze Age tell at Belegiš – Šančine ca. 30km south on the west bank of the Danube, but there are no published reports of investigations there to confirm its precise chronology. The LBA cemetery of Belegiš Stojića Gumno lies 800 metres from this tell, demonstrating continued activity in its immediate environs after 1600 BC [60].

#### Pančevo-Najeva Ciglana and Zlatica–Omoljica

Brief comments can be made about the excavated settlements at Pančevo-Najeva Ciglana and Zlatica–Omoljica. The former lies on the outskirts of the town of Pančevo, towards the southwest margin of the TSG. Stratified deposits were not recovered and the site can generally be dated to the MBA on the basis of Vatin pottery recovered. The site appears to have been small, however, according to Ljuština [96]. Zlatica–Omoljica also lies at the margin of the TSG system, close to the Danube. This was a larger site with multiple phases and after the MBA phase with Vatin pottery, a phase with SDŽB pottery was identified. It is unclear if there was a break in occupation between these two ceramic horizons, but whether continuous or as a new establishment, this early LBA occupation of a specific location that was occupied in the MBA is very rare in the surrounding area. There is nothing to indicate that this settlement was of particularly high status. No clear abandonment date within the MBA has been defined for either site [96, 97].

#### Foeni and Butin

Located just north of the east-west flowing Timiş river on the Romanian side of today’s Serbia-Romania border, the Foeni site complex consists of a tell-like settlement, a tell, and a flat settlement [98]. These were sequentially occupied and were all located along the same river terrace. The tell-like settlement (Foeni-Cimitrul Ortodox) was enclosed, had Neolithic origins, and though Bronze Age layers were heavily disturbed, it appears to have been limited to late Early- to early Middle Bronze Age habitation. Sporadic finds of Belegiš I ceramics indicate low-level activity there in the LBA [98]. Established at the same time as the tell-like settlement was abandoned, a small tell (Foeni-Gomila Lapului I) to the north continued in use until the end of the MBA with ceramics of the Vatin family documented as predominant rather than the Maros or Otomani-Füzesabony group ceramics common in the Pecica polity. The final phase of occupation documented in Gogâltan’s test trenches was dated by the presence of SDŽB pottery dated to LBA 1 [99].

Following the abandonment of the tell, a new flat settlement (Foeni-Gomila Lapului II) was established nearby which had Belegiš I and II pottery [99]. This demonstrates important continuity across the MBA to LBA horizon at the Foeni complex, yet a clear rejection of the tell as a focus of activity. It is unclear from aerial imagery if the LBA settlement conforms to the TSG form, but a site of that characteristic form can be found 6km to the south at Cruceni (Cruceni-Modusi Ut). Mention should be made of the partly-destroyed site of Butin to the south of Foeni and east of the TSG. This may be a MBA tell, but there are few data regarding its date apart from probable finds of Vatin pottery [100].

#### Židovar

Equally little is published yet for the southernmost tell investigated in the Pannonian Plain at Orešac-Židovar (S2 File). It is located, similar to Mošorin-Feudvar, on an isolated part of a high loess terrace above the valley of the river Karaš [101]. The site was occupied by 2000 BC, with density of activity increasing in the following centuries on the tell and in an outer lower settlement that was constructed at this time, as well as the creation of surrounding terraces. There is 3.5m (out of 5.6m overall) of Bronze Age stratigraphy at Židovar, of which MBA levels are dominated by Vatin pottery forms and are 2m deep [57, 101, 102]. The use of Vatin pottery suggests some similarities in the expression of identity to the community at Foeni and earlier MBA phases at Mošorin-Feudvar, while also marking this as notably different to Pecica and the later phases of Mošorin-Feudvar. This relates to both local pottery production and the use of exogenous styles in community dynamics at Židovar. Following the MBA phase, the next stratigraphic horizon skips several centuries and is characterised by black-burnished pottery of the Belegiš II-Gava tradition, dated after 1200 BC [57]. This is the same gap as recognised at Mošorin-Feudvar. The chronology of the lower settlement is unclear beyond a general contemporaneity, partly because of disturbance by Early Iron Age pits. Some Belegiš II-Gava ceramics were also recovered. As with Mošorin-Feudvar, the final MBA phases were disturbed by natural and later activity, but there is currently no published evidence to confirm re-occupation of the tell after 1500 BC and prior to 1200 BC when Belegiš II-Gava pottery was introduced.

#### Vatin and Bugarska Humka

Vatin (Vatin Bela Bara) is an oddity as it should be documented as a tell-like settlement because it is largely flat in today’s landscape, but with over 2 metres of stratigraphy, it conforms to Gogâltan’s definition of a tell [66]. An original settlement plan associated with Vatin pottery was reconfigured at a time when later MBA Crvenka- Cornești (1850–1600 BC) pottery was adopted. This created complex stratigraphic relations, compounded by a coarse sandy geology which made phasing of occupation horizons difficult. Occasional finds of Chalcolithic pottery are known from the site, but the earliest architectural features identified were built ca. 1900 BC. The small excavation windows at Vatin indicate it was densely occupied and served as a central site for its immediate hinterland. The results of recent excavations are being prepared for publication by one of the present authors (DJ) and we can state here that the site was abandoned in the last decades of the 17th century calBC. After that, a cemetery of LBA date was established immediately above parts of the MBA settlement, with burial pits commonly dug into the ruins of houses. The earliest burial was an inhumation with metalwork including a sword, a miniature battle axe and a needle datable to the 15^th^ century BC [103–105]. A second LBA cemetery (Vatin-Selo) was created on the right bank of the river about 0.5 km to the northwest. Nonetheless, no LBA settlement has been located nearby. Finds from the cemetery reveal continued occupation of the hinterland, with vessels of both SDŽB and Belegiš I-II styles recovered.

Ca. 12km to the southwest of Vatin, a small tell or tell-like settlement at Bugarska Humka was identified in our survey. Surface finds include later MBA (Cornești-Crvenka) ceramics, which also occur in the surrounding fields. The size and abandonment phase of the site are unclear, but no LBA pottery was found in the vicinity.

### Late Bronze age settlement–current knowledge

The LBA settlement archaeology of the wider Carpathian Basin is less well-researched than the MBA and so our knowledge of LBA society is commensurately less well-developed. Much focus has been on typo-chronologies of ceramics and metalwork, commonly derived from cemeteries and hoards, with little evaluation or theorisation of the LBA social and political landscape. In terms of contextualising our survey, therefore, we rely primarily on recent excavation projects that have focussed on higher-order, or simply larger, monumental settlements.

A key reason that so little is known about LBA settlements is their low visibility on the ground–most are simple flat settlements. Indeed, those LBA settlements with visible ramparts were the first to attract attention through excavation in recent years. This monumentality was a key design feature for many LBA settlements, as testified by the extensive use of ditches for enclosures [106]. Nonetheless, when no / small ramparts accompanied them, the infilling of diches over time means they have very low surface visibility today. Surviving upstanding ramparts are rare and are associated with the larger and most monumental sites, the extreme case being Corneşti Iarcuri at ca. 1765 ha [107]. This was the largest construction built in any part of Europe up to that time, dwarfing the citadels of the Aegean world and their surrounding towns. Other impressive sites, while smaller, were nonetheless massive relative to earlier and contemporary sites in Europe, with sites such as Sântana Cetatea Veche, Gradište-Iđoš, Sakule, Crepaja, Bašaid, Csanádpalota, Orosháza Nagytatársánc and Újkígyós all warranting the term megasite or megafort, ranging from 75 to 460 Ha of enclosed space (Fig 1) [50, 89, 108].

Recent excavations within some of these sites have provided new knowledge on LBA building episodes, design of space and domestic economies. At Cornești Iarcuri, evidence for LBA domestic structures and activity more generally is very limited despite its massive size. Food was clearly produced and consumed at the site based on finds of querns, archaeobotanical and zooarchaeological assemblages, but there are no indications as to whether this was permanent or occasional occupancy [109]. Similarly at Csanádpalota, despite its massive size of 460 ha, the evidence for permanent occupation within is very limited [108]. It should be stressed that their enormous size means a tiny fraction of these sites has been subject to excavation and that surface prospection is impacted by soil formation and cultivation activity. At both sites, there remains a possibility that the architectural traditions in a landscape devoid of stone may have resulted in poor archaeological visibility, particularly if they were not burned. The argument for poor architectural visibility is reinforced by the extensive development-led excavations at Șagu, where masses of domestic and industrial refuse were recovered in over 300 pits, but no structures were defined in the open-area excavations.

At the 130ha site of Sântana, located north of the Mureș, exceptionally wide ramparts were constructed that are unique in the area and the excavators point to similarities with Terramare ramparts in northern Italy [19]. Geophysics revealed three large buildings of ca. 50m length on their long axis and between nine and 20 other buildings in the inner enclosure (I), demonstrating the possible presence of monumental buildings, but also a notably different use of space than at TSG sites. The outer enclosures (II and III), however, have very low density buildings (of unknown date) indicating a similar concern with maintaining enclosed open spaces [89, 110]. In southern Hungary north of the Mureș river, the LBA sites of Orosháza Nagytatársánc (two enclosures ca. 105ha + 85ha, overall 190ha) and Újkígyós (ca. 70ha) are very large enclosed settlements that have many visible pale activity areas and multiple enclosures. Although their spatial organisation is slightly different, they incorporate a small enclosure found at many larger TSG sites.

At Gradište Iđoš, excavation has focussed on the enclosure ditches, yet the activity areas discussed below are indicative of domestic structures clustered together, leaving large parts of the enclosed space apparently unoccupied. Two habitation areas are defined by ditches along with an embanked citadel area and these are entirely enclosed by further ditches, defining a space of up to 200 ha. Gaydarska and Chapman argue that the absence of evidence for buildings at Gradište Iđoš should be read literally as evidence of absence and they correlate this with the dearth of visible structures at Cornești Iarcuri and Csanádpalota to suggest these sites were not settlements [111]. They contend that the primary function of the largest enclosed sites was as a place of assembly. However, viewing Gradište Iđoš as one of 100+ settlements in Banat with similar occupation traces defined by pale ‘activity areas’, and with reference to Orosháza Nagytatársánc and Újkígyós in Hungary, their theory cannot serve as a rule for all large enclosed sites / megaforts of this area.

Turning to cemeteries, of those in use during the MBA in the broad environs of the TSG, most were abandoned by 1600 BC [41, 48]. Unfortunately along with the dearth of MBA settlements, there is a dearth of known cemeteries of MBA date in the immediate proximity of TSG sites. Nonetheless, the establishment of new cemeteries after 1600 BC is a robust statement of new death and lifeways characteristic of the LBA and we can identify many that were established then and used until ca. 1200 BC (Fig 3). Only a small few continued to be used at a reduced level after that time [38].

**Fig 4:**
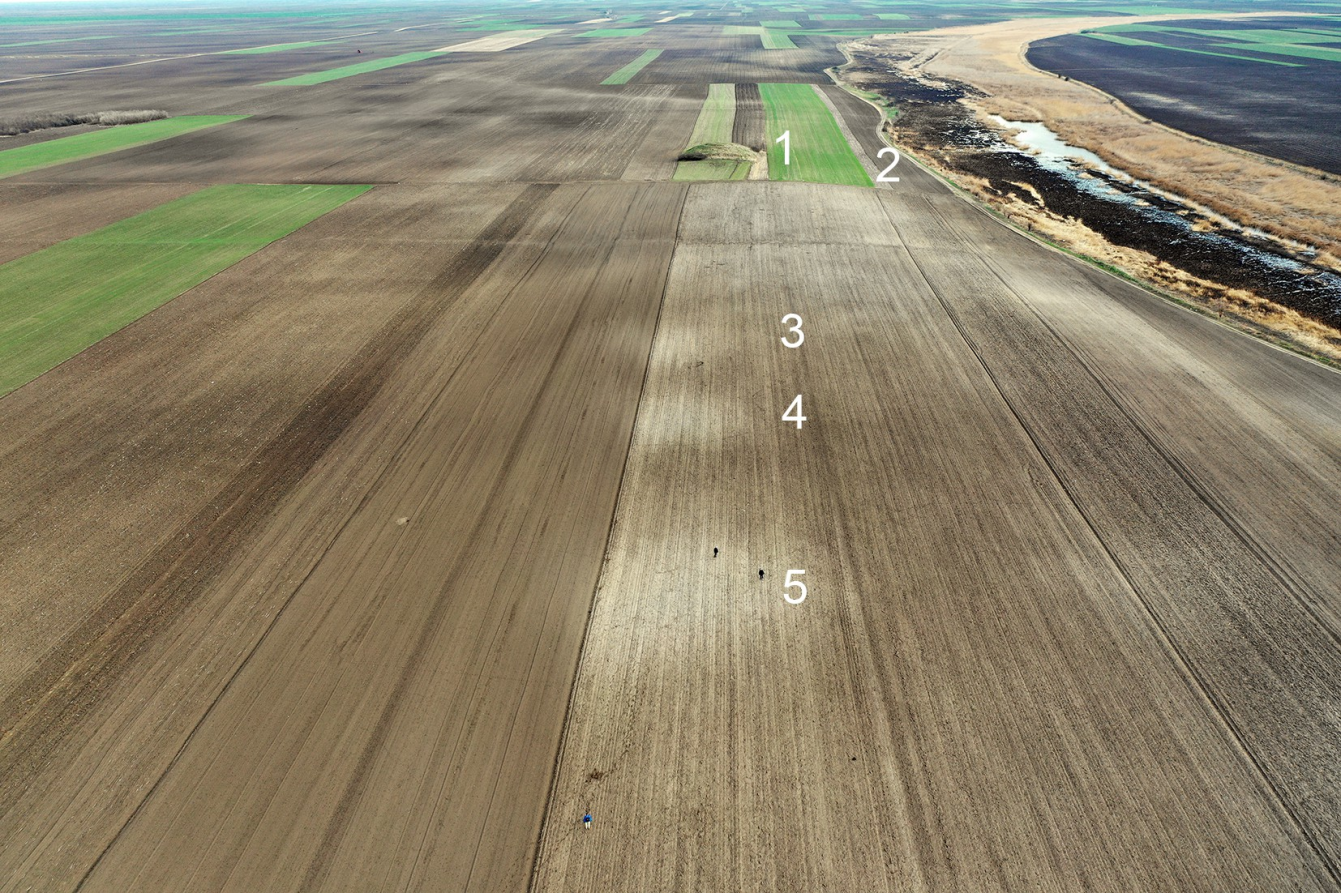
Central area in TSG site of Baranda showing 1) Tumulus, 2) watercourse and 3–5) activity areas aligned with tumulus (Photograph by Barry Molloy).

The relationship of LBA settlements and cemeteries to the tumuli situated within and around them remains unclear because no tumuli have been excavated and published in the hinterlands of the TSG [112]. Despite the lack of chronological determination, if we accept they could be prehistoric and pre-LBA, their spatial relationship with LBA settlements and cemeteries suggests that tumuli played an important ideological role in LBA social landscapes (e.g. Figs 3, 4 and S3 File). For example, the flat cemetery of Budžak Livade beside Gradište Iđoš lies along the path of a tumulus alignment, which in turn follows a paleochannel. Tumuli throughout the area commonly align with water channels, with the greatest density associated with the largest East-West flowing water courses, with important alignments terminating near to the megaforts of Gradište Iđoš and Sakule (Fig 3). Considering excavated tumuli from the wider region, it is probable that large diameter and tall examples date to the 4^th^ to early 3^rd^ millennium BC and some of those with 10-20m diameter and low mounds (1–1.5m in height) may even be LBA in date. This possibility is based on the same metalwork styles found in tumuli with those proportions dated to the 14^th^ to 13^th^ century calBC in areas south of the TSG along the Western Morava and tributaries of the Sava to the south and Susani-Grămurada at the edge of the Pannonian Plain to the east of Cornești Iarcuri [58, 113–116].

All size ranges of tumuli were built in close proximity to each other, occurring both within and immediately adjacent to many TSG sites. This defines a complex palimpsest landscape of settlement and mortuary sites. Whether tumuli are all older or if some are near-contemporary to the settlements, we see co-location as stating a claim–recent, invented, partly-constructed or entirely appropriated–to an ancestral landscape that had not been related to tell communities. LBA communities could thus “borrow” legitimacy by using places of the dead. Establishing flat cemeteries similarly created new ancestral relationships in time and space. These moves allowed new narratives of landscape, continuity and legitimacy to emerge based on strategic locational choices. This was conceptually in opposition to the continuous occupation of domestic spaces that had been a defining characteristic of MBA tells.

## Materials and methods

In order to better understand the settlement network in its entirety, we must contextualise the better known large sites within a continuum down to the smallest ones [15]. With this in mind, we initiated the *Survey In the Lower Tisza* (SILT) project in 2019 combining remote prospection and ground truthing of prospective sites. This is not an exhaustive mapping of all LBA activity in this landscape as our objective was to identify those settlements with specific features visible through remote prospection.

Below, we follow the general LBA chronology established by Gogâltan [117] as follows: LBA 1 = 1600–1400 BC, LBA 2 = 1400–1200 BC and LBA 3 = 1200-1050/1000 BC. The 16^th^ century is problematic because in many schema this is counted as MBA and we acknowledge that the MBA-LBA boundary is nebulous. In relative chronological terms, Belegiš I and tumulus culture (HGK) correspond to LBA 1, Belegiš II to LBA 2, and SDŽB pottery spans both.

The identification of archaeological sites through aerial prospection, pioneered by D. Grosman, began in the early 2010s and further LBA sites of TSG form have previously been recognised through survey [118–122]. For this study, prospection began with Google Earth historical imagery. While some sites were visible year-round, images from February to May were the most suitable for identifying characteristic features. A close association between site location and water courses was observed, with most sites set back around 20km or more from the current course of the River Tisza, to avoid its flood plain. Identified settlements were typically set along paleo-water channels or close to low-lying and marshy areas. Where higher ground / loess terraces were present, sites tended to occur at the base of the terrace. These basic criteria refined our prospecting, enabling us to locate sites with lower surface visibility.

Subsequently, one of the authors (M. Estanqueiro) used satellite remote sensing (Sentinel-2 data) analyses for site detection to identify a little over 100 candidate archaeological sites with pale soil marks on both sides of the Tisza [122]. By analysing the spectral signatures of known TSG sites pale soil marks and comparing them with their immediate surroundings it was possible to detect differences in reflectance, translated in different soil proprieties, and identify the months where the soil marks are more visible. Because band combinations do not always allow the direct visualization of archaeological features, several vegetation indices were also calculated, and principal component analysis (PCA) performed. Focussing on candidate sites identified through various remote prospection on the east of the river, more than 100 TSG sites have been documented (Table 2) alongside 10’s of similar sites identified to the east and west of our survey area. To confirm their chronology, we visited or used literature and museum records to confirm the chronology of 101 out of 106 TSG sites identified by remote prospection (Fig 3).

**Table 2:** List of TSG sites documented during the SILT survey project.

ID	TSG Site Name	Min. Size (ha)	Number of enclosures	Number of activity areas	Water course	LBA 1	LBA 2	General LBA	Latitude	Longitude
1	Crna Bara	42	1	15–20	x			x	45.97670	20.25754
2	Mokrin 3	55	0	30	x			x	45.94787	20.32330
3	Čoka	160	1	0	x	x			45.93653	20.14473
4	Mokrin 2	9	0	10–15	x			x	45.91933	20.49381
5	Mokrin	58	6	35–45?	x	x	x		45.91379	20.45231
6	Jakovo	28	0	25–35	x			x	45.85464	20.55008
7	Gradište Iđoš	200	6	30–40	x	x	x		45.85713	20.39694
8	Iđoš 2	34	0	35–40	x	x	x		45.80753	20.32282
9	Kikinda	71	0	20–40?	x		x		45.78746	20.39917
10	Novo Miloševo 3	15	1	15–20	x	x?	x		45.74840	20.37785
11	Novo Miloševo 4	38	2	10–20	x		x		45.74331	20.22906
12	Banatska Topola 4	5	0	10–15	x			x	45.70569	20.44317
13	Novo Miloševo 2	47	1	15–20	x			x	45.71086	20.39010
14	Banatska Topola 3	11	0	20–25	x			x	45.72751	20.40453
15	Banatska Topola	13	1	24	x			x	45.69475	20.48779
16	Banatska Topola 5	15	2	14	x		x		45.67364	20.41582
17	Banatska Topola 2	12	1	10–15	x			x	45.66359	20.47486
18	Novo Miloševo	22	0	20		x?	x		45.65814	20.34712
19	Matejski Brod	10	0	12	x			x	45.64871	20.17923
20	Bašaid 2	22	2	15–30				x	45.64518	20.51593
21	Aleksandrovo	58	1	30–35				TBC	45.65321	20.66501
22	Novi Bečej	16	1	20			x		45.64272	20.31899
23	Novi Bečej 2	17	0	22				x	45.62960	20.33274
24	Novi Bečej 3	33	0	30	x			x	45.62745	20.25880
25	Bašaid	85	3	32	x			x	45.62510	20.46533
26	Bašaid 3	7	0	8	x	x	x?		45.61534	20.35762
27	Torda 2	40	1	25	x		x		45.60686	20.51261
28	Torda	80	0	44	x		x		45.59412	20.40506
29	Kumane 3	14	1	8	x			x	45.58640	20.29116
30	Melenci 5	25	0	30	x	x?	x		45.58388	20.34165
31	Kumane 2	6	0	9	x	x	x		45.58298	20.24921
32	Melenci 6	18	0	6	x			x	45.57749	20.35082
33	Melenci 3	10	0	17	x	x	x		45.57413	20.38839
34	Melenci 2	12	1	24	x	x	x		45.56576	20.30702
35	Melenci 4	12	0	15	x	x?	x		45.56355	20.32533
36	Međa	9	0	5	x			x	45.55145	20.81445
37	Međa 2	6	0	5–10	x			TBC	45.57995	20.80102
38	Srpski Itebej	24	0	12	x	x			45.55003	20.74978
39	Turija	25	2	40	x		x		45.54692	19.85128
40	Borđoš	95	4	40	x		x		45.52034	20.11632
41	Banatski Dvor	30	1	20–25	x		x		45.51812	20.47097
42	Kumane	16	1	19	x		x		45.51661	20.27269
43	Žitište 2	26	2	8	x		x		45.51061	20.53169
44	Novi Itebej	60	2	5–10			x		45.50705	20.71010
45	Melenci 7	4	0	5	x			x	45.50153	20.43775
46	Melenci	16	1	18	x		x		45.49760	20.34888
47	Jankov Most 2	67	1	20–30	x		x		45.48591	20.41151
48	Srpski Elemir	18	0	40	x			x	45.47298	20.31197
49	Srpski Elemir 2	30	2	14	x	x	x		45.43601	20.27508
50	Žitište	80	3	22–30?	x		x		45.47209	20.56016
51	Klek	32	3	15–30	x			x	45.46524	20.49064
52	Jankov Most	70	1	35	x		x		45.45617	20.45269
53	Čurug	32	1	20	x			x	45.45460	20.05486
54	Zrenjanin 4	15	1	15–20	x		x		45.44584	20.35642
55	Zrenjanin 5	15	0	15	x		x		45.44079	20.45095
56	Zrenjanin 3	12	0	25			x		45.43617	20.40266
57	Klek 2	11	0	14	x	x	x		45.41632	20.46439
58	Aradac	13	0	20	x			x	45.39809	20.32499
59	Sečanj	97	1	25–30	x	x			45.37611	20.79632
60	Zrenjanin	37	1	20–25	x			x	45.36570	20.46090
61	Zrenjanin 2	15	1	13				x	45.35245	20.46679
62	Boka	12	0	15	x		x		45.31462	20.76295
63	Tomaševac	20	0	17	x			x	45.26614	20.69696
64	Jarkovac	24	0	11–15	x	x	x		45.26026	20.78821
65	Dobrica	89	2	40–45	x	x	x		45.23487	20.86365
66	Uzdin 2	11	0	17	x			x	45.23223	20.59409
67	Plandište	25	0	25–30				x	45.19878	21.15020
68	Perlez	13	0	10	x			x	45.20024	20.40634
69	Dobrica 2	20	0	17	x			x	45.19170	20.84757
70	Uzdin	33	0	20–25	x		x		45.17731	20.59596
71	Sakule 2	18	0	10	x		x		45.17315	20.49075
72	Lokve	10	0	10		x	x		45.16198	21.06496
73	Idvor	54	1	37	x			x	45.15983	20.57294
74	Čenta	10	0	15	x	x	x		45.15803	20.41989
75	Čenta 2	20	1	10	x			TBC	45.16413	20.38427
76	Sakule	90	4	25–35?		x	x		45.11842	20.56245
77	Baranda	13	1	14	x	?	x		45.09619	20.51211
78	Baranda 2	48	0	25–30	x			x	45.08679	20.55004
79	Baranda-Bandaška	15	0	5	x			x	45.07029	20.43525
80	Opovo	30	0	13	x			TBC	45.05726	20.55772
81	Debeljača	11	0	23			x		45.05960	20.64733
82	Crepaja 3	75	0	20				x	45.05713	20.68236
83	Nikolinci	70	1	25–30	x			x	45.05188	21.08438
84	Crepaja	85	4	35–45	x	x			45.03401	20.66446
85	Sefkerin	30	1	20	x	x	x		45.03051	20.58079
86	Crepaja 2	18	0	15–25				x	45.01482	20.67443
87	Sefkerin 2	11	0	15–20			x		45.01265	20.52171
88	Kačarevo	40	2	20–30	x	x	x		44.99890	20.70814
89	Glogonj 2	14	0	15–20	x			x	44.99523	20.58593
90	Jabuka 2	30	2	4				TBC	44.96940	20.61239
91	Jabuka	56	1	30–35			x		44.98470	20.65362
92	Glogonj	80	1	17-?	x	x	x		44.98083	20.55402
93	Kačarevo 2	40	1	15–20	x	x	x		44.95195	20.71743
94	Banatsko Novo Selo	34	1	15–20	x			TBC	44.95872	20.81021
95	Pančevo	70	1	10	x	x			44.91953	20.75413
96	Pančevo 2-Stari Tamiš	25	0	8–10	x	x	x		44.88922	20.76719
97	Dolovo	10	0	9-?	?			x	44.88521	20.80095
98	Starčevo	21	1	6	x			x	44.85490	20.73478
99	Starčevo 2	3	2	2	x	x			44.84328	20.71649
100	Pančevo 4	42	1	10–15				TBC	44.84916	20.79799
101	Vranin-Salaš	11	0	10–20	x			x	44.86930	20.74643
102	Mramorak	34	0	29	x	x			44.86911	20.98912
103	Mramorak 2	8	0	8	x			x	44.85828	21.00802
104	Bavanište	30	1	14	x	x	x		44.84320	20.87960
105	Bavanište 2	21	1	12–15				x	44.82207	20.90449
106	Bavanište 3	70	1	20–30?	x	x			44.79761	20.94300

Ground truthing was influenced by accessibility of sites and agricultural cycles as ploughed land was typically required to allow for visibility of sherds and even modest rainfall made many sites inaccessible safely. Fieldwork involved non-systematic surface prospection by walking across the full extent of candidate sites and collecting diagnostic ceramics. Visibility of finds during site visits was dependent on how recently and in what manner the soil had been processed (e.g. ploughing) as well as how wet the soil was. At some sites visited more than once, it was observed that visibility of material could change substantially. In general, a dense concentration of pottery would be around 5 sherds per square meter, but more commonly even in activity areas (see below) this was closer to 2–3 sherds per square meter on sites with good visibility. Ceramics were collected by site only and no spatial data was documented.

**Fig 5:**
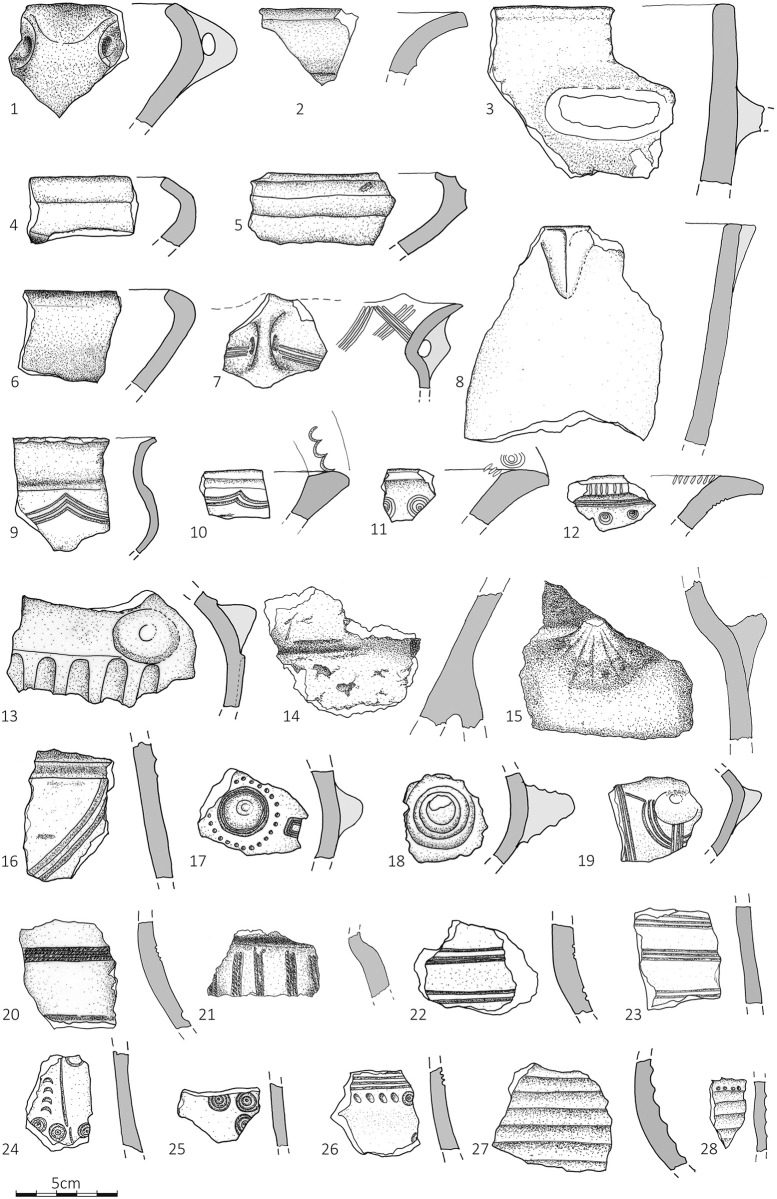
Selection of pottery from ground truthing survey. Gradište Iđoš = 1, 6, 16; Novi Bečej = 2; Jankov Most 2 = 3, 13; Žitište = 4; Crepaja = 5; Uzdin = 7; Sefkerin = 8; Dobrica = 9, 10; Bavanište 3 = 11, 12; Jankov Most 2 = 13; Bašaid 3 = 14; Melenci 7 = 15; Kumane 2 = 17, 18; Sakule = 19, 23; Jarkovac = 20; Čenta = 21; Kačarevo = 22; Mramorak = 24; Kumane 2 = 25; Novi Bečej 2 = 26; Melenci 4 = 27; Zrenjanin 4 = 28. (Drawn by Dragan Jovanović).

The vast majority of sherds were undiagnostic body sherds. At some sites hundreds of sherds were visible and a sample based on diagnostic features was taken. At other sites, fewer than 20 were visible and collected from the entire settlement and the range of diagnostics was therefore limited. Low visibility of sherds may relate to surface conditions (ploughed vs. unploughed) or depth of deposits and so quantity of finds retrieved was not interpreted as indicative of intensity of ancient activity. As a consequence of visibility and recovery biases, sites attributed only to the LBA 1 or to the LBA 2 phase in Table 2 demonstrate the presence of material from a given phase but do not indicate with confidence that activity during the other phase was absent. At some sites, there were insufficient diagnostic sherds to date the assemblage beyond a general LBA (that is LBA 1–2) date (Table 2, Fig 5 and S4 File).

**Fig 6:**
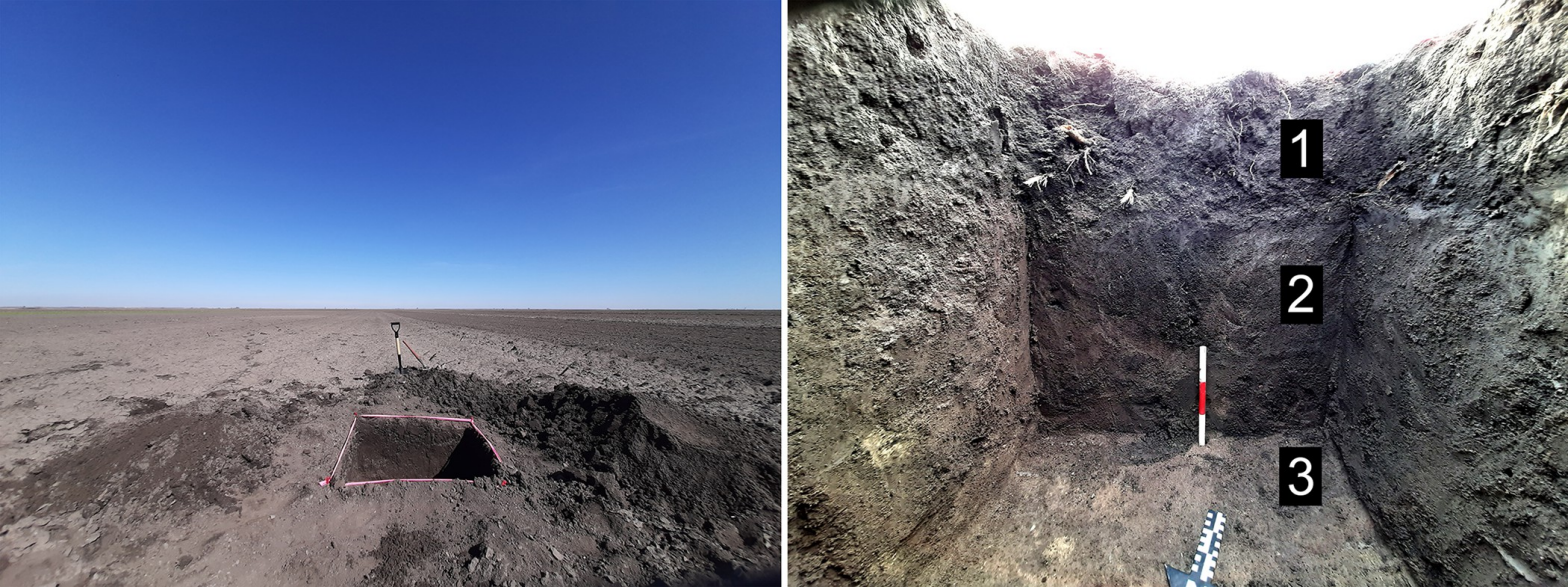
Test trench 2 showing depth of habitation debris and lack of clear stratigraphy within an activity area at Sakule (Photograph by Barry Molloy).

As part of our prospection program, absolute dates were required to confirm a general chronology of sites. Samples were obtained from 1x1 metre test trenches at Sakule, Bavanište and Kačarevo 2, from ongoing research excavations at Gradište Iđoš, Sakule and Žitište, and from developer-led works at Stari Tamiš and Turija Gradište. The 1x1m trenches were excavated into activity areas to ascertain the depth of cultural deposits and to recover material for 14C dating from contexts associated with ceramics below the plough-zone (Fig 6). This provided a control on the frequency of ceramics within the topsoil versus those visible on the surface of the topsoil. Due to the extensive ploughing at sites, our dates do not represent the final phase of activity areas but rather the final undisturbed phase of each feature. These dates confirmed the relative dating based on ceramic finds. Samples were taken also from cremation and inhumation burials associated with highly representative ceramics from the cemetery of Budžak Livade beside Gradište Iđoš and spanning the LBA 1 and 2 phases.

## Results

**Fig 7:**
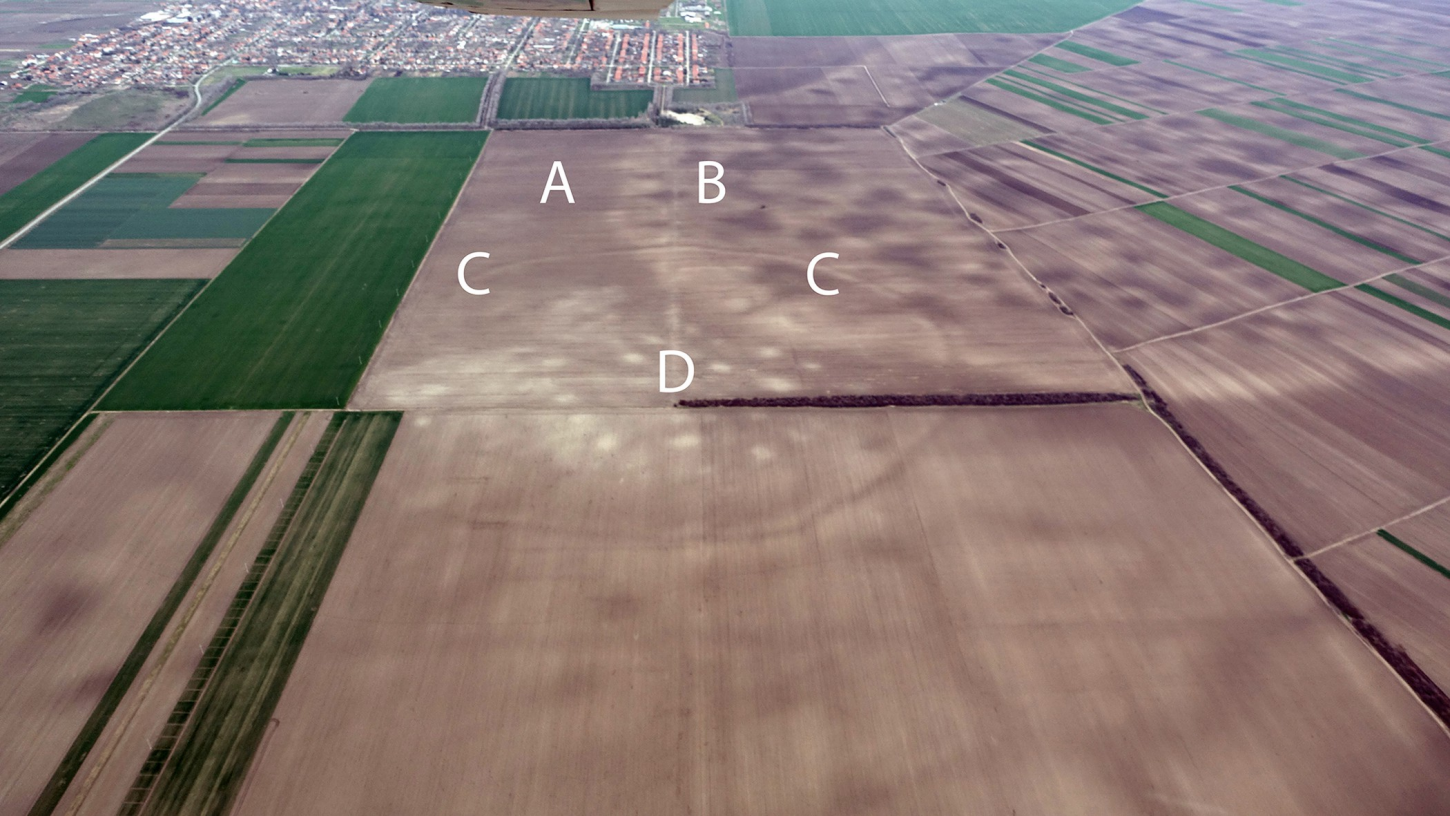
TSG site of Kačarevo 2 showing: A) Low-lying land, B) raised sand-plateau, C) enclosure ditch, D) activity areas (Photograph and key by Barry Molloy and Darja Grosman).

Particularly favourable soil conditions in Banat enabled us to use remote prospection to identify sites with highly consistent and recognisable features. First, the most common element is a patchwork of roughly equidistant but irregularly arranged sub-circular patches of pale soil measuring ca. 20-30m in diameter. These occur in clusters of ca. 10–30 patches. Second, the pale patches are commonly enclosed by one to several ditches, commonly with traces of flattened ramparts, visible in remote imagery and / or natural watercourses or soil ridges (Fig 7 and S2 File). Ditches can also serve to internally sub-divide spaces, as sometimes visible in aerial images.

Surface material (most commonly pottery, bone and ground stone) was concentrated on these characteristic pale patches and find density sharply dropped off away from them. We therefore termed these ‘activity areas’. Our preliminary test excavations at the settlement of Sakule indicate they were the location of disturbed domestic structures, with patches of floor level and possible rammed-earth wall bases tentatively identified. The pale colour arises from a mixing of the underlying loess subsoil, topsoil and probably other organic components used to build structures.

Our remote prospection approach is affected by a visibility bias. Only sites that present particular features can be recognised using this form of prospection. Some sites with feint / difficult to recognise activity areas have become known to us following developer-led excavations–notably Banatski Dvor–or through targeted intensive (area-specific) systematic surface prospection [123–127]. These surveys have also demonstrated that LBA domestic activity was taking place throughout the wider landscape and was not focussed solely within TSG settlements. Small, remote sites such as individual farmsteads no doubt existed also, as seen in the intensive survey of the Titel Plateau [95]. Nonetheless, we would argue that the comparatively large footprint of TSG sites relative to other known sites defines them as central places within their hinterlands and their dense distribution suggests that the entire landscape was very closely integrated and controlled by their builders.

### Characterizing the tisza site group

The 100+ TSG sites identified thus far in Serbia align broadly on a north-south axis on the east side of the Tisza-Danube north-south corridor. Sites with identical design occur immediately to the east between the Bega and Timiş rivers in Romania (Fig 3). They are located in similarly low-lying parts of the landscape, and whilee not visited on the ground as part of this project, their chronology has been confirmed by Dorogostaisky and colleagues [119, 120]. A handful of sites of this form have been identified thus far on the west side of the Tisza in Serbia, in Bačka county, including the settlement at Turija which had features dated to LBA 2 based on ceramics and absolute dates (Fig 1).

**Fig 8:**
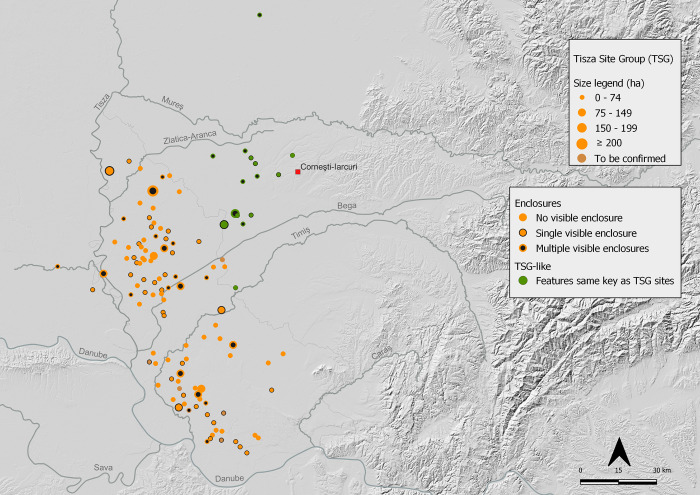
Size and visible features of TSG and TSG-like sites in Banat and east Backa. All definitions refer to features visible in aerial imagery. Single-enclosure sites have one complete or partially visible ditch outside of activity areas. Multiple-enclosure sites have two or more complete or partially visible ditches. Sites with no visible enclosures refers to detection using remote prospection visibility only and has not been ground truthed. (Map by Marta Estanqueiro and Caroline Bruyere; basemap hillshade derived from ALOS DSM AW3D30, reprinted from https://www.eorcJaxaJp/ALOS/en/dataset/aw3d30/aw3d30_e.htm under a CC BY license, with permission from JAXA -Japan Aerospace Exploration Agency, original copyright 2023).

On the basis of aerial imagery, TSG sites range in size from ca. 6 ha to as much as 200 ha. Based on the interquartile range from all known TSG sites, the visible footprint of activity derived from remote prospection at most sites is in the range of 10–50 ha (Fig 8). In the case of unenclosed sites at the lower end of this spectrum size is calculated according to the minimum *visible* activity zone (extent covered by activity areas) and excludes the equivalent ‘empty’ space that lies between activity areas and boundary ditches at enclosed sites. The smallest defined site had only 2 (possibly 3) activity areas at Kumane 3 and the largest is Gradište Iđoš at nearly 200 ha which has multiple enclosures and two distinct contemporary enclosures surrounding activity areas.

**Fig 9:**
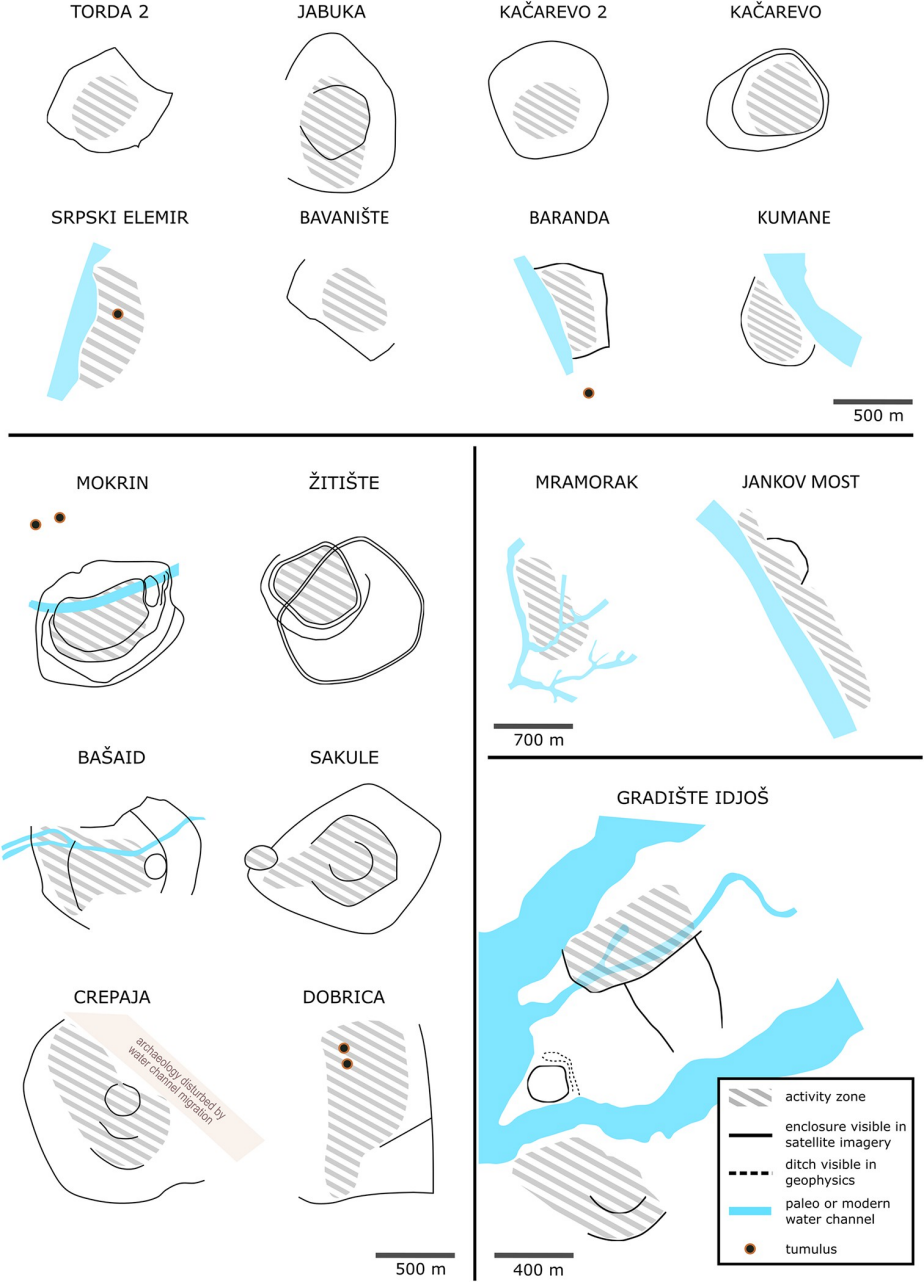
Outline of a selection of TSG sites showing range of organisational features including ditches and natural watercourses. (Drawn by Caroline Bruyere).

**Fig 10:**
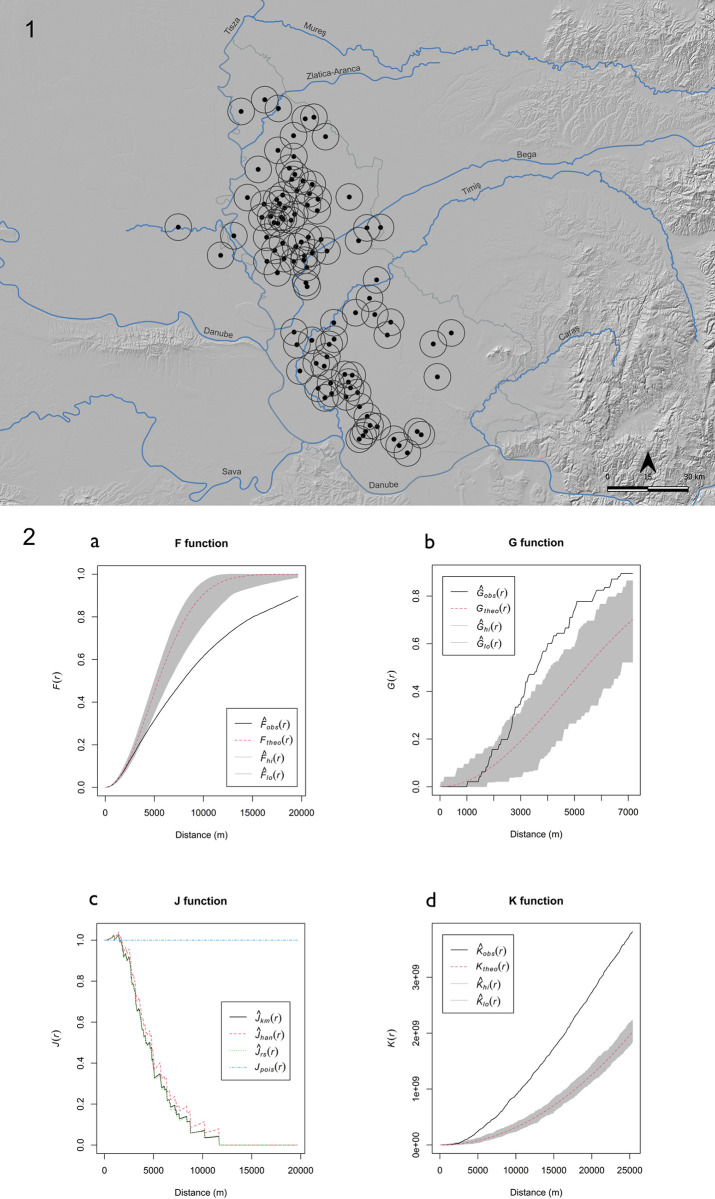
1) 5km radius range around TSG sites. 2) Distance-based cumulative distribution functions. For F, G and K functions—black lines: observed values; red lines: theoretical values; gray values range: Monte Carlo envelopes considering 99 simulations. For J function—blue line: theoretical values; black line: Kaplan-Meyer estimate; red line: Hanisch estimate; green line: reduced-sample estimate. (Map and graphs by Marta Estanqueiro; basemap hillshade derived from ALOS DSM AW3D30, reprinted from https://www.eorcJaxaJp/ALOS/en/dataset/aw3d30/aw3d30_e.htm under a CC BY license, with permission from JAXA -Japan Aerospace Exploration Agency, original copyright 2023. Graphs plotted using R 4.2.0).

Enclosed settlements are most commonly sub-circular to sub-oval in plan (Fig 9), though they are occasionally sub-rectangular with one or more straight boundaries (e.g. Dobrica and Bavanište). Ditches are commonly V-shaped in profile, in some cases shifting to U-shaped near to entrances, and are usually between 2 and 3 metres deep (from the modern field surface) and 3 to 6 metres wide at the top. Some enclosures can be irregular in shape when utilising landscape features, including watercourses and slopes (e.g. Melenci, Torda 2, Mramorak). Sites can also include a combination of these landscape features (e.g. Gradište Iđoš, Jankov Most, Novo Miloševo). Around 50% of sites, usual smaller ones, have no enclosure visible in aerial images, 30% of sites have one enclosure, while larger and more complex sites can have up to four concentric or sequentially accessed enclosures, as seen at Crepaja, Sakule, Bašaid, Mokrin and Gradište Iđoš for example (Figs 3 and 10). An estimated 30–40% of the megafort at Crepaja has been eroded in the northeast through the migration of a now-extinct minor watercourse. XXXX MOVE FIGURE REF HERE XXXXXXXXXXXXXX? Fig 9: Outline of a selection of TSG sites showing range of organisational features including ditches and natural watercourses. (Drawn by Caroline Bruyere)

A functional hierarchy can be recognised in site design, whereby the larger sites are invariably more complex than smaller ones and they invariably have spatially more extensive and intensive surface traces of activity (apart, perhaps, from Bašaid). Internal divisions are defined by enclosures visible either in aerial imagery or through geophysical survey [50]. The variety of site locations, from gentle slopes to isolated islands on the edges of wetlands, may also suggest that apart from size and complexity, different sites had different roles or special purposes within this network.

Activity areas commonly lie 20–40 metres apart and are rarely more densely spaced or overlap. They occur from the geometric centre of a site in a cluster and their distribution stops at least 40, and up to 100, metres away from enclosing ditches (when present). This indicates that a specific zone with no activity areas was usually retained around habitation loci but within the boundaries of a settlement, creating intentionally “empty” spaces. Fernández-Götz has recently discussed the relevance of such divisions and empty spaces for understanding complexity at non-urban settlements [128, 129]. The density of activity areas is relatively consistent across sites and so the number usually, but not as a rule, correlates with the size of the enclosed area if present. This trend can be broken when sites are spread along a watercourse rather than clustering around a spot beside it, as seen at the Jankov Most, Novo Miloševo or the Melenci clusters of sites. Intensive systematic surveys of a sample of settlements conducted by C. Bruyere and geophysical survey data by J. Pendić and G. Topić will shed further light on spatial layout and density of activity within some enclosures in publications in preparation.

All sites are set within active agricultural lands today that are subject to ploughing, thereby damaging them annually and impacting on preservation and visibility of features. Despite this, at some sites activity areas were slightly raised above the surrounding surface indicating that many, and perhaps all, had once been positive features standing proud of the surrounding ground level. Test excavations at Sakule confirm that activity areas were a locus for sequential phases of activity, similar in concept but different in execution to intergenerational modes of tell occupation.

TSG sites are densely spaced in the landscape, and the vast majority lie within 5km of at least one other known site (Fig 10.1). Their close proximity, and the fact that the catchment areas (5km radius) overlaps in most cases, suggests that the communities that lived there were closely related, most probably through kinship but certainly politically. This clustering created a very dense societal network that controlled not only the surrounding territory at the local scale but this entire landscape at the larger regional scale, including resources and communication routes. This in turn demonstrates the presence of a complex political system that integrated, managed and served the needs of multi-local communities living within and around hierarchically, and possibly functionally, differentiated settlements.

At a visual level, Fig 10.1 suggests that, similar to the distribution of activity areas within a settlement, the distribution of settlements in the landscape was not random. In order to evaluate if this was systematic clustering, several spatial functions were estimated and plotted. From a statistical perspective, a completely random spatial pattern can be described by a homogeneous Poisson process with intensity λ, so we computed several distance-based cumulative distribution functions to explore the spatial distribution of settlements and their departure from this hypothesis.

For this assessment, G (nearest neighbour function), F (empty space function), J and Ripley’s K (detection of spatial patterns at multiple distances) functions were chosen (Fig 10.2). The first three are based on nearest neighbour distances, while the K function is based on distances between all pairs of points (settlements). Point-wise Monte Carlo envelopes were also computed. As we can observe in Fig 10.2, the empirical lines for settlement distribution indicates a trend towards an aggregated/clustered pattern. The F function assumes that if the settlements are randomly spaced, so is the empty space between them. Therefore, if a clustered pattern is present, the empirical line would be below the theoretical one, as seen in Fig 10.2A. The opposite can be said of the G function, where an empirical line above the theoretical one indicates clustering (Fig 10.2B). The J function is a combination of the previous two, and values of J(r) < 1 would suggest clustering (Fig 10.2C).

In order to assess the spatial pattern at different scales, Ripley’s K function was also used. This can help detect more complex patterns at different distances, not only at nearest-neighbour scale. In this case, the Fig 10.2D suggests a clustering trend at multiple scales. For our data we observed that until approximately 3km the empirical lines followed the theoretical ones, after that, there are indications of clustering. We can also see that most settlements have a nearest neighbour within a 6km (approximate) distance. This tells us that site distribution was unlikely to be random and that close spacing / proximity was a function of a social strategy more than the unplanned emergence of settlements within each other’s hinterlands. An element of horizontal movement of communities is plausible within the duration of our quite broad chronological blocks of 100–200 years, though this would not explain the relative uniformity of the clustering across this social landscape.

The gap between a northern and southern cluster of sites in the land between the Bega and Timiş rivers appears to be an ancient phenomenon. Whether that was socially and / or topographically dictated is unclear, but the clustering of tumuli along the southern bank of the Timiş suggests this line was meaningful in prehistory (Fig 3). Following the chain of TSG-type sites between the Bega and Zlatica rivers to the east in Romania, the easternmost sites are situated in the immediate vicinity of the megafort of Cornești Iarcuri. The settlements of Carani, Biled and possibly Satchinez Râtul Popii are of TSG type and lie 8, 24 and 19 km respectively to the west of Cornești Iarcuri. Despite the uniqueness of this latter site, its location at the end of this chain of sites with similar designs indicates a social relationship.

### Climate change and the new domestic landscapes of the Tisza Site Group

Nicodemus and O’Shea [77] observed that MBA tells around the Mureș and Tisza rivers commonly targeted landscape features that were raised, and Ljuština [57, 101] argues the same for Židovar, thereby occupying natural islands within a marshy and wet hinterland. This created spatial discontinuity by land, emphasising the importance of riverine transport. We might assume–on the basis of settlement distribution alone–that certain areas of the landscape in the MBA were undesirable due to seasonally or perpetually marshy conditions. For example, this avoidance may have arisen from (perceived) poor suitability for their farming conventions or weaker connections to major rivers, which were important elements in MBA lifeways and communication networks.

**Fig 11:**
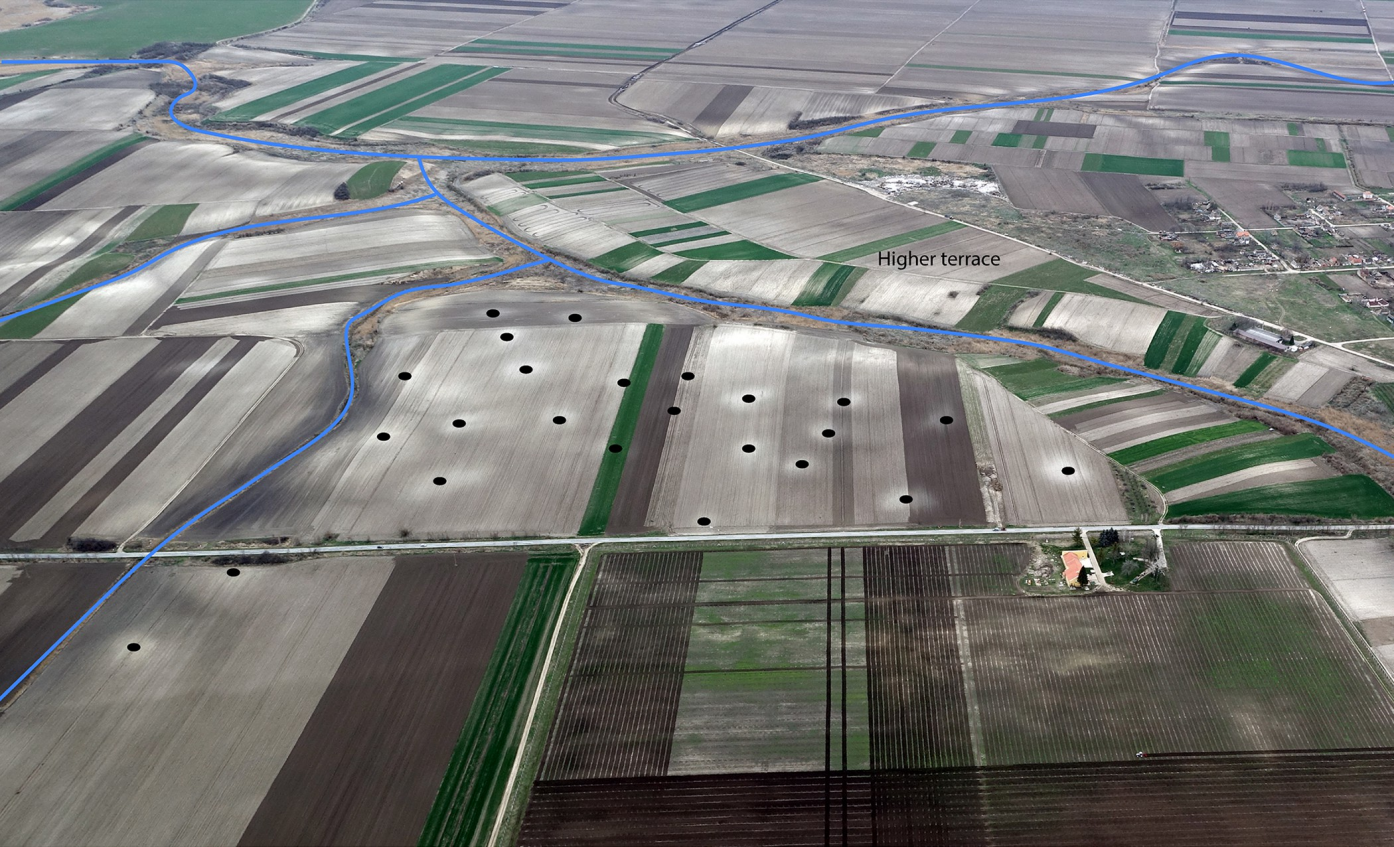
Aerial image of Mramorak showing water courses, activity areas and site location at base of higher terrace (Photograph and markup by Barry Molloy with thanks to Darja Grosman).

Locational choices, including use of landscape features, changed substantially in the LBA as areas with previously low-density occupation became very high-density settled landscapes. Many TSG sites incorporated water channels running through them. In cases where water channels did not pass through sites, they commonly ran immediately adjacent (Table 2). Particularly in the southern cluster, where a terrace is present with flat areas above and below, sites are commonly located at the base of the slope separating the two levels, for example at Bavanište, Dobrica, Pančevo, Pančevo 2, Dolovo, Mramorak, and Mramorak 2. When a more gradual slope is present, some sites have elements at the base and along the slope, for example Kačarevo 2 (Fig 7) and Sakule. At major sites such as Crepaja and Sakule, as well as smaller sites, substantially higher ground on sandy terraces lies within ca. 5km to the east, but these were evidently rejected as settlement loci, with few exceptions e.g. Bavanište 2. The elevation variance between the base and top of terraces close to sites commonly exceeds 8 metres height differential. The characteristic ditches that surround sites may have been intended to aid drainage, although this was unlikely to be their primary function in light of water channels passing through some sites and others ditch circuits being built on sloping ground (Fig 11).

The topographic location of most sites is either within or beside some of the lowest points in the landscape and this landscape is in turn one of the lowest lying and flat expanses of the Pannonian Plain, which is the lowest lying part of the Carpathian Basin (S2 File). Remote imagery reveals myriad palaeo-channels alongside natural and artificial waterways active today. While geomorphological investigation into which waterways were active in the Bronze Age has not been undertaken, the location of prehistoric tumuli along smaller channels indicates many were flowing in the Bronze Age. Historic maps and records for the area demonstrate that much of the province of Banat was subject to flooding and standing bodies of water prior to large-scale drainage works in the 19^th^ century AD and subsequent upgrades [130, 131]. The levees on the River Tisza were constructed to a height of 5 metres to prevent flooding and lateral movement of the now culverted water course [132]. Prior to this, over 35–50% of the Serbian province of Vojvodina, covering much of the southern Pannonian Plain, was under water, marshland or regular threat of flooding, whereas today 92% is under cultivation [130, 133]. Even so, flooding remains a regular seasonal threat in the south Pannonian Plain, particularly in low lying areas including former marshes, cut-off channels and oxbow lakes of old river courses. The risk of flooding is, and must have been in prehistory, unpredictable with some years being considerably more difficult to navigate than others. The development of soils in the Banat region supports high groundwater levels [134]. Retention of surface water due to a high water table after a rainy period was a further potential risk for the inhabitants of the TSG sites, perhaps greater than flooding of major nearby rivers. Historical records also show a contrary pattern whereby a substantial reduction in rainfall can have substantial short-term impacts on soil hydration and productivity due to the interconnected nature of waterways and their relevance to the water table across a wide area [133]. Most TSG sites are located on deposits formed in the Late Pleistocene, indicating alluvial deposition from flooding was not a major factor shaping Holocene topography at those locations [135]. Alluvial deposits of Holocene age have been documented near to the sites along present-day river courses.

With the low-lying topography and relationship to a network of water channels and possible seasonal or perennial open bodies of water in mind, the builders of these sites established a wetland-oriented settlement strategy which took advantage of the particular environmental conditions this offered. De Marinis [136] and Dalla Longa [137, 138] both observe for the contemporary Po Valley a choice to occupy low-lying areas associated with water courses (Terramare sites) or to build houses on stilts within areas of standing water (Palfitte sites). This reflects what Dalla Longa calls [138] “a clear intention to set up settlements along main river courses or on their banks … sites are located near or within wet basins, following the “wetland-oriented” settlement strategy well-known in literature”. This similarity in settlement strategy may be a further reflection of systematic cultural, ideological and probably economic exchanges linking these two exceptionally large and flat plains [139–141]. Furthermore, this switch from the avoidance of low-lying areas in the MBA to embracing them in the LBA can be read as changes to worldviews and cosmologies, potentially including the integration of previously liminal spaces as inhabited spaces. The highly similar locational choices for almost all TSG sites, however, also reveals a dearth of risk spreading, particularly in light of their apparent co-dependence.

In the case of the TSG, these settlement strategies suggest that communities exploited a finely balanced ecosystem that was enabled by the prevailing climatic conditions between 1500 and 1200 BC. Though requiring systematic environmental research, paleoclimate markers for increased rainfall indicate that areas that had been marginal marshy lands could have become more predictable bodies of water, thereby becoming a resource rather than a liminal or hazardous space. This hypothesis may also be supported by the relative stability of hydroclimate markers of the LBA relative to the MBA, which indicate conditions that would have reduced the risks associated with settling in close proximity to water courses and wetlands. As a cautionary note, it should be emphasised that the stable isotope data from the cited speleothem records primarily relates to wintertime rainfall levels and we lack proxies specific to summertime rainfall or temperature levels [21, 142]. If higher water levels were sustained annually this would ensure hydration of soil during the comparatively hot summer months, a phenomenon identified in other studies [35]. Future analyses of chironomid head capsules from wetlands, for example, may provide insights into summertime conditions in the Middle and Late Bronze Age to further explore this [143–145]. We argue that the prevailing climate in the 16^th^ century BC encouraged decisions to engage in novel land use regimes. Emergent social strategies no-doubt balanced myriad societal factors, so that climate and environment should be read as enablers operating at a multi-decadal scale that facilitated, but did not determine, adaptive human choices [30, 146, 147].

There are insufficient environmental data to model the conditions in the TSG landscape at this time, but the settlement location choices themselves tell us two things about the environmental conditions. First, they were stable enough to allow sites to be located at low-lying elevations (relative to water courses) without substantial risk of flooding, indicating reasonably predictable conditions. Second, the relationship of people to their environment was different to the Middle Bronze Age, during which time these same topographic locations were actively avoided.

In the highly similar environment of the Po Valley, it has been observed that the cooler (not cold) but humid conditions of 1500–1200 BC created an environmental niche in which particular forms of society developed. The practices and ideologies of landscape use were tailored to supporting specific needs of communities within a specific hydroclimate and landscape hydrology niche. In the Po Valley, climate conditions with predictable winter rainfall were paired with a comparatively high water table in the LBA (Recent Bronze Age) relative to the MBA and Final Bronze Age [148]. Looking from the beginning of the TSG phenomenon, pioneering groups must have demonstrated an attractiveness to the new lifeway in this environmental niche that became attractive to others over time, leading to this unprecedented density of settlements.

There are convincing grounds to argue that climate change played a role in how change from MBA to LBA social systems unfolded and again during the time of the decline and abandonment of TSG sites from the 13^th^ to 12^th^ centuries BC. The decline of MBA communities was protracted and socially driven, but the changing climatic conditions were arguably less favourable to their modes of using the landscape. This was not a catastrophic change and indeed it may even exemplify resilience and adaptability, whereby societal scale change was needed to successfully adjust to a new normal [34, 149]. The MBA system had long-since entered a crisis state by the 16^th^ century BC and so rather than narrowing options, climate change appears to have broadened them providing alternative pathways to prosperity. These were readily exploited but appeared to correlate with a fundamental reorganisation of settlement and society. LBA settlement locations led to infilling and linking up of areas between the distinct tell-networks (or perhaps polities) characterised by the use of Maros pottery in the north and northwest and Vatin pottery in the south and east. There was evidently sufficient agricultural land located around LBA settlements to sustain this very dense network of communities.

A second factor influencing site location appears to have been the desire for colocation with pre-existing mortuary landscapes, tumuli in particular (Fig 4).This may have enabled LBA communities to differentiate their ideologies from those of MBA entities and create a neutral linchpin to aid integration of a heterogenous population base. An association between tumuli and watercourses is also evident, for example in the hinterland of Glogonj, Mramorak, Novo Miloševo 4, Idvor, Melenci 2 and Mokrin. This suggests a three-point relationship for settlement choice in many cases, requiring watercourses and tumuli. Concentrations of tumuli along water courses served a dual purpose as navigation points marking river-land cross-roads and as boundary markers signalling control over rights to disembark boats or to cross between politically differentiated areas.

### Relative chronology

The chronology of the pottery we collected during site visits confirmed those exhibiting all TSG features were all LBA 1–2 in date. With very few exceptions, those that had clustered activity areas (with no ditches) were also LBA 1–2 in date. It is unlikely that all TSG sites were occupied at the same time, and there must have been an element of horizontal movement of communities building sites. However, if this occurred within a short space of time–even decades–the resolution of neither the absolute nor relative chronologies is precise enough to document this. The regular spacing observed between ‘activity areas’ within the confines of each site suggests that all, or most, domestic spaces were occupied simultaneously or within living memory.

Our dating of sites relies on diagnostic surface finds (Fig 5, Table 2 and S4 File). Belegiš I and II pottery are found throughout the TSG area. As noted by Szentmiklosi [55] and Falkenstein [94], SDŽB pottery occurs in the areas surrounding the TSG in the south, and our survey demonstrates it was consumed quite commonly within the southern cluster of sites. Distribution of ceramics of this broad family of encrusted pottery can be mapped now in an area extending from the Danube to south of the Bega river. It is present, but less frequent, in the northern cluster of TSG sites and is exceptionally rare in the northernmost TSG sites.

Considering our survey finds, there is a greater frequency of sites that have only Belegiš I / SDŽB pottery along with relatively fewer or no sherds of Belegiš II in the southern site cluster (Table 2). It is emphasised that this can be a bias of the recovery of diagnostic sherds, as only a handful of diagnostics were recovered from sites that only had Belegiš I pottery. Nonetheless, the difference in assemblages between sites north and south of the Bega appears to be a real phenomenon. This can be explained in two ways. The first is that the making of Belegiš II pottery and displacement of Belegiš I occurred gradually during the 14^th^ century BC, with the stylistic trend spreading from the north while SDŽB was still being made in the southern cluster, thereby blurring or offsetting the LBA 1–2 ceramic transition more there than it does in the northern cluster. The other possibility is that a higher proportion of sites in the south were abandoned before LBA 2.

Pottery indicates that the earliest TSG sites in both clusters were occupied early in LBA 1 and that there was an increase in activity, and possibly site construction, at the beginning of LBA 2. Based on both relative and absolute chronologies, all TSG sites appear to have been abandoned in the decades around 1200 BC. Of these, only the largest sites at Gradište Iđoš, Sakule and possibly Bašaid have indicators of reoccupation in small areas during the 11^th^ to 10^th^ centuries BC. The site of Jaša Tomić is an exception as this exhibits TSG features but was (probably) a new foundation of 12^th^ to 10^th^ centuries BC based on pottery recovered.

A common feature at TSG sites was reoccupation in either the Sarmatian (final centuries BC) and / or Medieval periods (broadly 1100–1400 AD). The Sarmatian and Kumani (11^th^ to 13^th^ centuries AD) groups are known to have extensively practiced mobile pastoralism [150, 151]. Re-use of prehistoric tumuli and construction of new ones in this area is a phenomenon of the 11^th^-13^th^ centuries AD, potentially related to the documented arrival of non-Christian Kumani groups from the Steppes [50]. Other periods were very rarely represented at TSG settlements–notably the Neolithic–potentially indicating related concepts of landscape and land use occurred only at specific points in the history of the region.

### Absolute chronology

**Fig 12:**
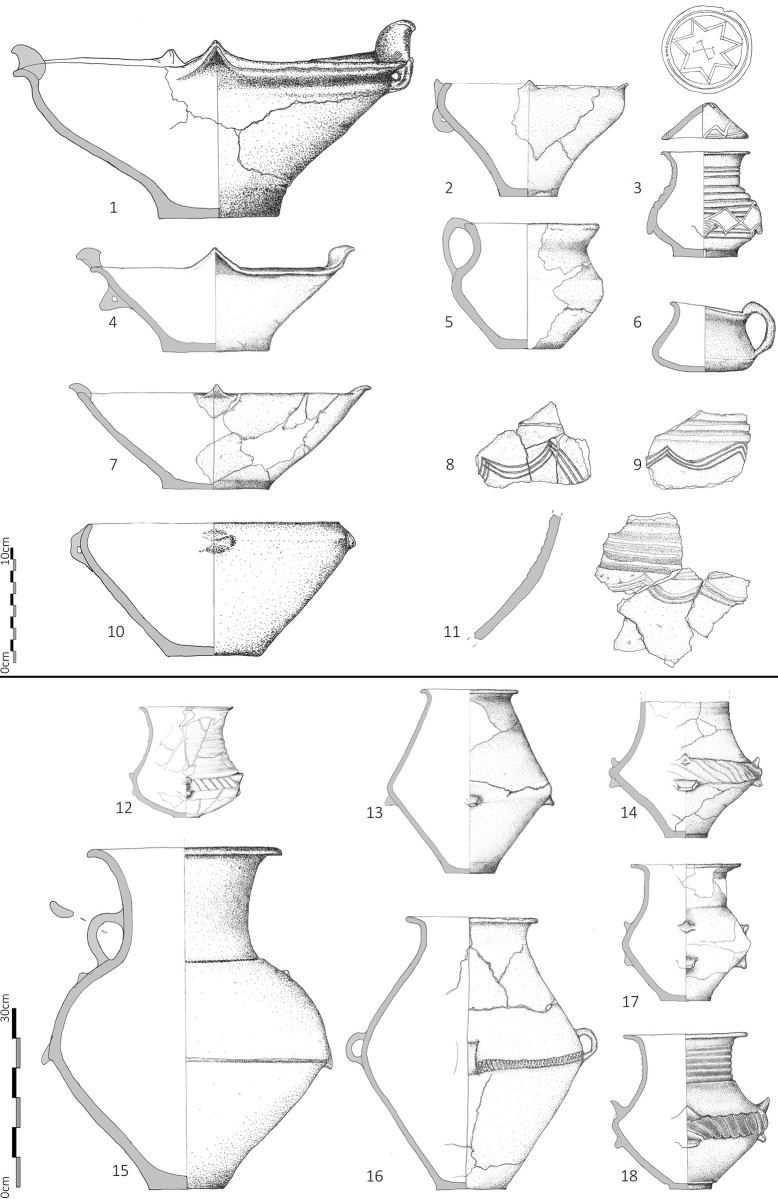
Pottery from dated contexts at Budžak Livade. Grave 2: 3, 6; Grave 9: 4, 16; Grave 12: 1; Grave 22: 10, 15; Grave 23; 18; Grave 24: 2, 5; Grave 26: 12; Grave 29: 17; Grave 31: 8,9; Grave 32: 14; Grave 33: 13, Grave 36: 7; Grave 37: 11 (Drawn by Dragan Jovanović).

**Fig 13:**
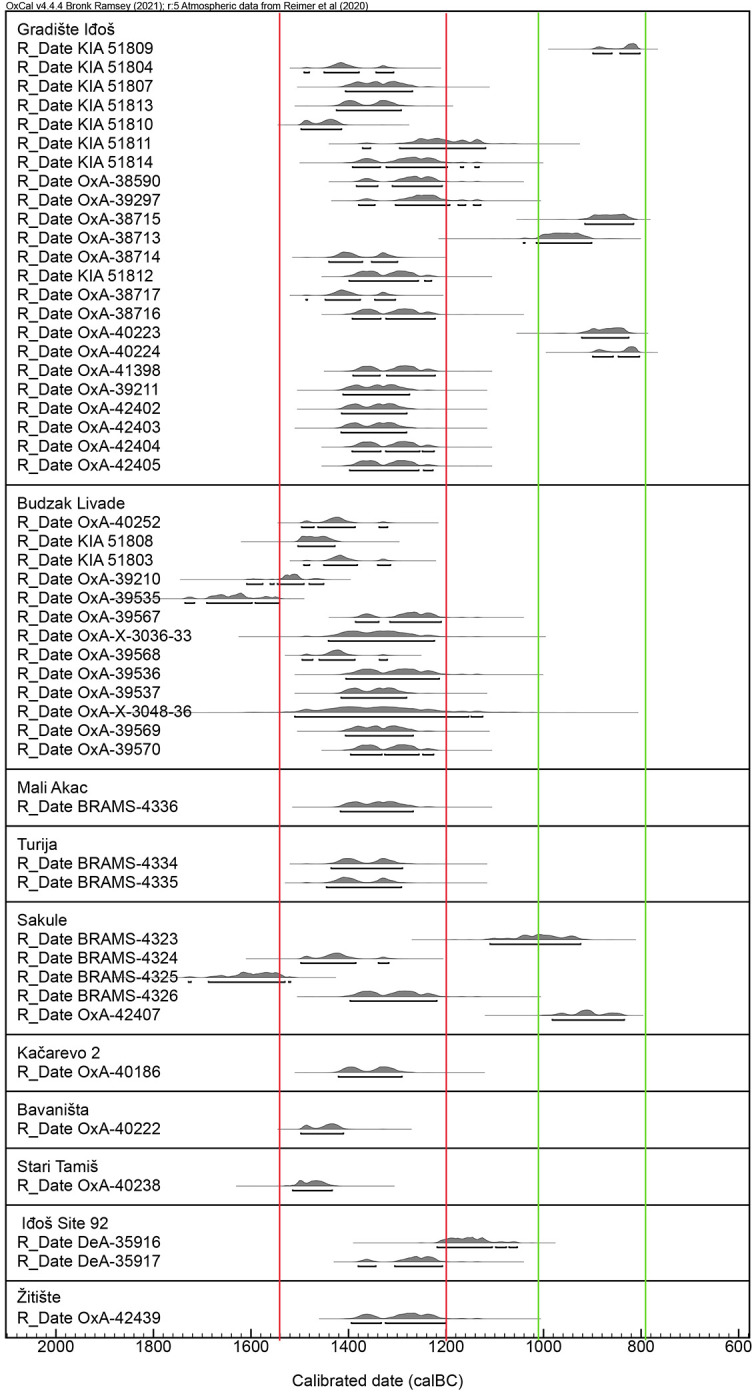
Absolute chronology of TSG settlements and cemeteries. (Figure by Christopher Bronk Ramsey).

Defining an absolute chronology for the TSG sites is difficult given the number of sites, their complexity, and their massive size. This is compounded by the dearth of scientific excavation. Absolute dates from well-stratified contexts at Gradište Iđoš and the nearby cemetery of Budžak Livade provide a general framework for the occupation history of one such community. Ceramics from graves with absolute dates include vessels of Belegiš I and II and of tumulus culture forms, typical to the LBA assemblages of the northern TSG area. This LBA 1 and 2 chronology is contrary to Daróczi et al.’s [152] recent unsubstantiated re-attribution of tumulus culture ceramics from this cemetery to the MBA. The cemetery was established by 1600 calBC and it continued in use into the 13^th^ century calBC (Fig 12 and S1 Table). The earliest occupation thus far excavated at the settlement is 15^th^ century calBC and there are no dates later than the decades around 1200 calBC, until a limited 10^th^-9^th^ century calBC reoccupation (Fig 13). In his regional survey of absolute dates, Sava defines a 16^th^ century calBC date for the first sites using Belegiš pottery, with increased numbers in the 15^th^ century calBC and a rapid drop off of sites in the 13^th^ to 12^th^ centuries calBC [38]. This drop off in site numbers after 1200 BC is also suggested in survey and some excavation data for nearby parts of southern Hungary [41, 108, 153]. Absolute dating at other sites in the TSG network, while the product of small windows of excavation, reveal a complementary picture. Details of sample type, context and uncalibrated date are provided in S1 Table. All necessary permits were obtained for excavation and sampling from the Ministry of Culture and Information of the Republic of Serbia, complying with relevant regulations for archaeological research (Permit numbers: 631-02-154/2021-02, date 08.11.2021.; 631-02-94/2019-02, date 28.10.2019.; 631-02-146/2022-02 date 27.10.2022).

**Fig 14:**
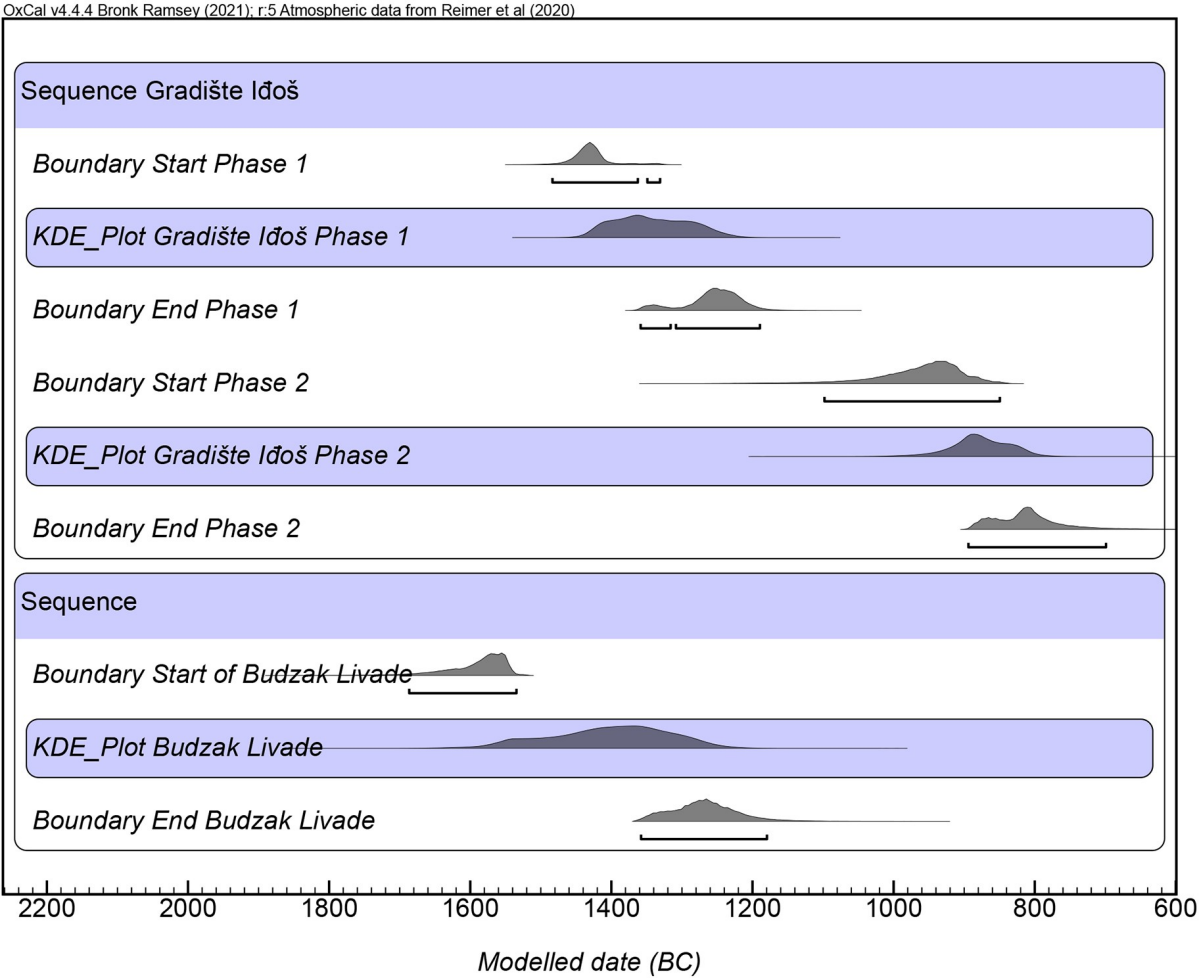
14C phase model for Gradište Iđoš and Budžak Livade (Figure by Christopher Bronk Ramsey).

At the settlement of Sakule, test excavations were focussed on activity areas. These returned dates covering the full duration of the use of Budžak Livade and Gradište Iđoš (Fig 14). The site appears to have been first occupied at the MBA to LBA transition and dated contexts cover most centuries until ca. 1200 calBC. After that, the site appears to have been abandoned until the reoccupation of a small area from ca. 1000 to 800 calBC. A similar hiatus is observed at Cornești Iarcuri [16]. Dates from an activity area in the TSG site of Stari Tamiš reveal occupation at the turn of the 16^th^ to 15^th^ century calBC and the settlement of Bavanište was occupied since at least the 15^th^ century calBC. The inhumation burial from Mali Akač has an uncertain specific context, but is probably from beside a tumulus in close proximity to Novo Miloševo 4 settlement and this skeleton is dated to the 14^th^ century calBC.

Looking at absolute dates from the wider LBA settled landscape of the south Pannonian Plain, the increased intensity of activity from ca. 1400 calBC seen in dates from Gradište Iđoš is reflected in the chronology of other well-dated enclosed sites at Sântana Cetatea Veche, Corneşti Iarcuri, Csanádpalota and Turija Gradište as well as smaller unenclosed sites in Romania [38, 154]. There appears to be evidence for burning / and or infilling of ditches at several excavated sites in the earlier 13^th^ century calBC. Activity continues at all other well-dated sites in the 13^th^ century and at some possibly into the early 12^th^ century calBC. At the site of Iđoš 2, 14C data show it was occupied into the 12^th^ century calBC [155].

Absolute and relative dates suggest that TSG sites and cemeteries–and indeed most of the lower Pannonian network–were abandoned en masse around 1200 BC. It is noteworthy that this was precisely the time of the contraction of settlement on the Titel plateau and reoccupation of the tell at Mošorin-Feudvar. A similar pattern, based on surviving strata, is probably detected at Židovar, which was resettled. No re-occupation horizon is observed at tells in the area of the Maros group in the 12^th^ century BC. These changes indicate that knowledge of tell sites was sustained in societies during LBA 1–2 but they were marginalised as loci for settlement until another major societal shift took place after 1200 BC through which loci for LBA settlements were rejected.

## Discussion

Looking to the construction of social landscapes, rather than being an unnavigated drift away from one system to another, the introduction of LBA lifeways were an active rejection of MBA regimes. This is evident in 1) material culture, 2) mortuary rites, 3) cemetery location, 4) settlement design, 5) settlement inter-relations, 6) the landscape foci of activity, 7) congregation spaces and catchments.

In turn, when we consider macroculture as the long-term accrual of culturally specific habits within a given place, then the shedding of much of this in the LBA of the southeast Pannonian Plain must relate to 1) substantial inward migration bringing new ideas, 2) active rejection of old ideas by the same population, 3) a crisis-driven adaptation or 4) a combination of these. Many of the building blocks of LBA society were derived from MBA norms and practices, for example pottery craft, but how they were configured and embedded in the landscape gave a substantially different shape to these communities and their lifeways.

Kienlin [76] recently argued that the EBA-MBA tells represent a “long-term stability of a traditional way of life rather than Bronze Age communities fundamentally [being] different from everything that had come before.” This emphasises just how different in long-term perspective the LBA settlement network was. Their duration of several centuries suggests any notion of “experimentation” is implausible and so they represent an alternative means to exploit resources of this same landscape. Kienlin further defines this long-term heritage as particularly local rather than reflecting Mediterranean influences, as argued for by others [9, 156]. This in turn contrasts with the LBA when it is very clear that the lower Pannonian network links into long-distance networks that include Mediterranean societies. This is seen in novel mortuary practices along with metalwork and ceramics styles that link the region to the Adriatic ambit, particularly the Terramare groups of the Po Valley [139, 140, 157, 158]. Carpathian metalwork styles become influential throughout Greece alongside Italian ones in a wider stylistic network alongside elements of the Belegiš ceramic tradition in the north of Greece [62, 159–168]. Though this increase in frequency is set in a context of collapsing long-distance networks, they represent the expansion during the 12^th^ century BC crisis of pre-existing links. Few lead-isotopic data exist from MBA contexts in the Pannonian Plain, but there appears to be a shift in metal exchange networks, whereby particular networks are expanded (southeast Alps) and new ones are created in the LBA–and notably Cypriot copper is attested for the first time [141, 169–171].

The nature and design of settlements was also different, even if a desire to monumentalise central places was retained. Monumentality speaks of a community’s desire to aggrandise their position through display of energy and resources invested in creating spaces. In the case of tells, monumentality emerged through a succession of generations occupying the same space, so that legitimacy and power emerged from this materialised intergenerational heritage. Sites of the lower Pannonian network took a very different approach by building massive and enclosed settlements in previously unoccupied loci, commonly integrating tumuli that aligned them with mortuary (more than lived) ancestral landscapes. Monumentality was not co-constructed with ancestors but by harnessing a large labour force to create (short-term) and maintain (long-term) enclosure ditches and ramparts. This enabled the emergence of new social relations within a community, including hierarchies and other status / activity categorisations. Speaking of Bronze Age Crete, but relevant here, Driessen and Letesson document a trajectory of complexity arising from shifts through which “original coresidential corporate groups were progressively replaced by proximate corporate groups that eventually were supplanted by dispersed corporate groups” [172]. These latter are relevant to the lower Pannonian network as power brokers became more distributed throughout settlement networks.

Acts of building forts also had capacity for relationship building between communities as labour became a resource required to sustain a settlement, thereby making it a resource that could be exchanged or otherwise shared [173–175]. Furthermore, constructed defensible sites were rarely static physically or socially–their state of being was political because it related them to neighbouring and distant communities through acts of building and maintenance [15]. There are good grounds to consider the dense and interlocking network of TSG sites as creating local to regionally relevant “powerscapes”. Their creation was a transformation of each local landscape by actively enclosing spaces, but together these myriad, contemporary local acts came together to characterise the TSG as a coordinated, regional phenomenon. These sites created venues where acts of construction, the ensuing monument, its landscape setting and activities carried out within came together to convey the significance of these places and those who built them [176]. Translating that into a more refined model of political geographies at this whole-landscape scale is not yet possible and, as Driessen argues, requires a clearer sense of “at what scale(s) power was implemented and to what extent power co-varied with scale… gradually, incrementally or erratically?” [177].

The dense spacing of TSG communities went further than enclosing interior spaces because they also shared lands between sites, forming a massive defensible chain of sites, making a statement at a total-landscape scale. While this was relevant at an ideological level, this claim was also supported through force of arms [178]. The importance of preparing for and enacting violent conflict is evident through the major innovations in, and massive quantities of, weaponry that were developed hand-in-hand with the construction of new defensible central places throughout TSG and wider lower Pannonian network. Developments included the invention of metal helmets, shields and armour along with new forms of sword, spear and battle axes and the introduction of chariots [179–182]. As a package of settlement and material culture developments, this may reflect the awakening of a “territorial consciousness” in which the enclosed spaces and lands lying between them became open to possession as territorial entities bounded by geographical features, in this case major rivers [183–185].

Looking to internal organisation of settlements, from middle-sized TSG sites of 10’s of hectares up to Cornești Iarcuri, LBA sites retained substantial open areas within their boundaries. There was a physical difference between spaces with intense domestic activity and other enclosed spaces that were kept open. Enclosure ditches defined inside and outside spaces in the landscape, but they also created gateways and divisions within sites creating social topographies by manipulating movement of people into and through the complex [128, 186, 187]. Social topographies refer to how hierarchies are enacted and interpreted through physical space–controlling access was conceptual, not enforced, and was grounded in appropriate behaviour in particular spaces. Spatial divisions may encourage public recognition and self-concept to be formed through seemingly benign cultural processes (e.g. defensibility), but differences were reinforced in practice through managing venues for social encounters [188]. In larger sites, spaces were often hierarchically ordered from outer rings to innermost zones. The various spaces created and access to them must have related to rights, status or activities to be conducted; that is, not all spaces were created to be equal. In this way, status inequalities came to be distributed not only vertically, as in a hierarchy, but also laterally throughout the social landscape.

Moore and Fernández-Götz favour a multifunctional role to the kinds of open spaces within enclosures that were an integral feature of TSG settlements, ranging in purpose from specialist cropping to places for temporary assembly of people or animals [128]. Gaydarska and Chapman [111] favour a dedicated congregation role for the largest enclosed sites. The use of open spaces for gathering need not be an exclusive function of such sites and so Gaydarska and Chapman’s argument for having recognised places for congregation is important. Such multifunctional open spaces could serve for gatherings that were scalable from an intimately local site cluster using smaller enclosures to the regional-scale of representatives of many or all communities in the largest ones.

Survey evidence for Bronze Age activity in the landscape around these sites reminds us that they are focal points, not the only loci of activity. It is fully possible that we are looking at a semi-sedentary society in which mobility along this chain of sites by some of the population was a norm, perhaps part of animal herding strategies. The proportion of game animals (>10%) in LBA faunal assemblages further hints at this close articulation between settlements and less intensively managed parts of the landscape [22]. Given the flat landscape and its susceptibility to flooding, the ability to concentrate herds within enclosures may have been a form of risk management with group members being able to shift between sites locally or to larger ones in times of acute trouble. Motivation to focus people in different enclosures could equally arise from episodes of violent conflict.

Cornești Iarcuri remains a site of extraordinary size in this landscape, though its hierarchy of spaces mirrors that of the smaller centres in its hinterland. The paradox of this site lies in the dearth of evidence for occupation within and the massive labour resources to maintain it–these are wholly incompatible on current evidence [111]. There is a strong case to be made that it was a communal centre for many communities living within the lower Pannonian network, including those of the TSG. Indeed, the same pottery and metalwork styles were consumed and mortuary practices used in and around all of these settlements, indicating highly similar modes of social reproduction. In Gaydarska and Chapman’s model, the TSG settlements would provide a justifiable “congregation catchment” who could sustain a place on the scale of Cornești Iarcuri. In this way power was manifested through, rather than wielded from, this megasite during gatherings. In Graeber and Wengrow’s view, people may have followed “kings whose courts and kingdoms existed for only a few months of the year, and otherwise dispersed into small communities” [189]. A recent argument for the emergence of Cretan palaces may also relate to Cornești Iarcuri, whereby building shared megastructures compensate for emergent power asymmetries. Driessen and Letesson argue that “their very construction was a collaborative effort and an integrating process but they were also used to stage ceremonial practices … to advocate a symbolic equivalence” between settlement-community clusters that varied in size, power, and prosperity [172]. This served to downplay status differences in principle while reinforcing them in practice. Structures like Cornești Iarcuri brought power brokers together and provided a context for controlling this large settlement network and external relations. Given the upland setting of Cornești Iarcuri, it could also have served as a place of security, whether seasonally or as a crisis response, should flooding or violence upset the routines of life in lower lying areas. Seasonal aggregations at this and other sites in the network, using movable shelters, may well have been part of established societal routines and aid in explaining the ephemeral settlement traces at many of the largest sites [189].

This model takes very poor account of other important sites, such as Sântana Cetatea Veche or Csanádpalota, but our focus in this paper is on TSG type sites. They held a closer spatial relationship with Cornești Iarcuri than the above megaforts (Figs 1 and 3), though clearly each of these centres played important roles within the lower Pannonian network. We are only at the very beginning of attempting to define a political geography–defined as the interface between landscapes and politics–yet it is evident even now that status and functional differentiation between sites was structured and part of the self-understanding of these communities from the time they emerged. The largest sites in this network appear to have been built a century or so after the earliest, and so a developmental trajectory needs to be investigated as more absolute dates are obtained.

Many or most communities of the lower Pannonian network were mutually dependent and closely interwoven with each other and their shared landscape. Coupled with the large population this represents, the settlement archaeology implies the presence of complex social institutions and attendant power structures to manage interaction, cooperation, competition and violence [77]. The more complex sites must have performed some roles of contemporary urban centres with their specialised areas and features for specific activities from ritual to politics to feasting to defence. However, any temptation to call megaforts proto-urban implies a more “primitive” social order. This undermines our capacity to view phenomena like the lower Pannonian network as representing alternate trajectories of complexity based around a network of multifunctional or polyfocal centres [128, 190]. Earth and wood enclosures were to remain important as central places across Europe well into the first millennium BC, a duration that belies a perpetual state of being stuck at a spurious proto-urban level. This persistence and broad continuity of layouts (e.g. multivallate, open interior spaces) may reflect a contra-urban phenomenon that supported a different model of complexity.

### TSG collapse

The low-lying location of TSG sites within a very flat landscape afforded them low diversity and exposed them to highly similar risks, for example flooding. It was argued above that they emerged within a specific and relatively stable climatic niche and exploited this landscape in an extensive manner never practiced before then. The relationship is not directly causal; we argue that change in land use was opportunistic rather than forced and ultimately supported rather than drove changes in settlement organisation. Apparent climatic shifts ca. 3.6/3.5 and 3.2 kya indicate that the LBA was bookended by warmer and drier conditions [191–193]. These shifts were not extreme and nothing indicates they caused catastrophic social change. Taking a decadal perspective, however, it is plausible that the increasing aridification recorded in the 13^th^ to 11^th^ centuries BC incrementally compounded social stressors, becoming a force multiplier for other causes of social conflict. The exceptionally dense and co-dependent settlement regime of the TSG may itself have laid the foundation for competition and conflict to arise if resources became scarcer, even marginally so. The finely balanced TSG landscape is a good candidate for the classic overshoot collapse model where increasing productivity and population co-occur in a wet cycle only to be undermined by decreased productivity and capacity to sustain a population in a dry cycle [34, 194]. Cautious of the balance between documenting correlation and causation, it is salient that both settlements and cemeteries were abandoned en masse in the 13^th^ to early 12^th^ century BC in the lower Pannonian network (Figs 12 and 13). Social change occurred, and it was devastating for these communities. We may find hints of the responses to this crisis in the material record. The deposition of hoards of broken bronze objects is commonly considered to have been a response to threats to individuals (e.g. violent conflict) or whole communities (e.g. environmental changes) or as votive offerings (e.g. propitiating divine forces) across time and space in European prehistory [195–198]. A massive increase in hoarding in the south Pannonian Plain is documented precisely from the 13^th^ century to 12^th^ centuries BC [199–203].

Settlement location choices left the social system of the TSG in an inherently fragile state arising from a dependency on particular hydrological conditions [29, 31, 34, 147]. This is not to say people were not adaptable, but rather deviation from a given system in order to be adaptable undermined the organisational logic of the system. The level of change to tip such a fine balance and push TSG communities beyond their stability threshold appears to have been relatively small [204]. One hypothesis would be that a need to seek out resources beyond the cramped hinterlands of the TSG network led to a doubling down on mobile pastoralism as a lifeway. Whole communities could have taken advantage of mobility to maximise their productive potential in a changing physical landscape. This would have led to gradual disaggregation between lifeways and political systems, potentially over decades, but constituting a tumultuous transitional period, what Raffield has recently termed a “shatter zone” in social trajectories [205]. The pieces remained, but their order was broken.

## Conclusion

The 16^th^ century BC was a time of social upheaval in the Pannonian Plain, with the decline of long-established centres of power, climaxing with the abandonment of settlements and most cemeteries. Ancestral claims to places of the living and the dead to legitimate power in earlier generations were cut off permanently. In some areas this was accompanied by depopulation and the simplification of settlement networks. In the south Pannonian Plain, however, massive new monumental settlements were established alongside many smaller sites with similar features. These emerged together with both appropriated mortuary monuments and the creation of new cemeteries and mortuary rites.

We have discussed the discovery of a network of over 100 densely spaced settlements, many with large, monumental enclosures. Their society was evidently culturally permeable and they embraced material and mortuary heritage from disparate traditions. TSG settlements emerged in a particular climatic niche that enabled them to successfully occupy very low-lying parts of the landscape that connected them intimately with waterways and wetlands, promoting a wetland-oriented lifeway. Rising temperatures and increased aridity in the 13^th^ to 12^th^ centuries BC appear to have severely impacted on these niche human-landscape relations that sustained TSG communities and their relationships with one another. In a society where sun worship was paramount or even exclusive, we should also take account of the impact of severe shifts in weather patterns on beliefs, both in divine forces and the ability of social leaders to propitiate them [9, 81, 206].

There was a coherency to the development of the lower Pannonian network that indicates communities were participants in a social phenomenon with a regional political order. They were motivated to build massive, enclosed sites with different spatial zones within. Lands locked between this chain were an integral part of the network, with enclosures serving as focal points punctuating a widely exploited landscape. Sites served different roles, ranging from status to function based, and some may have been designed to support regular population dispersal and aggregation, potentially as seasonal phenomena linked to semi-sedentary lifeways. Constructing enclosures was part of a trajectory that began around 1600 BC, increased in intensity by 1500 BC, saw a consolidation phase around 1400 BC, began to decline after 1300 BC and came to an end point by ca. 1200 BC.

The TSG sites as a phenomenon are remarkable due to their visibility allowing them to be remotely identified and then for their extent to be defined and aspects of their internal organisation evaluated using remote sensing and basic survey data. This network of over 100 sites, impressive as it is in its own right, was participating in a cultural landscape that gave rise to the construction of the largest monuments of the entire European Bronze Age. More striking still is the creation of the TSG built environment that operated at a near total-landscape level through the chain of co-dependent monumental settlements. The legacy of these massive, monumental, defensible central sites of earth and wood with comparatively low-density occupation may be recognised in the hillforts of the European Iron Age.

## Supporting information

S1 File KMZ file with all TSG sites currently known. (KML)

S2 File 1) Record of flight track for taking aerial photographs documented using GPS for synchronising photographs and locations; 2) Extensive, low-lying drainage network and localised flooding in aerial perspective in March 2020 with archaeological site of Baranda in the margin of the left foreground and the modern town of Baranda in left nearer background; 3) Aerial view of Kačarevo; 4) Aerial view of Bavanište 2; 5) Aerial view of Mramorak; 6) Aerial view of Pančevo 2—Stari Tamiš; 7) Aerial view of Jabuka; 8) Aerial view of Bavanište; 9) Aerial view of Crepaja, 10) Aerial view of Debeljača; 11) Aerial view of Sakule. Central enclosure is centre field, small “citadel” enclosure is in left background. Footprints from pedestrian survey grid visible within enclosures; 12) Aerial view of Sefkerin; 13) Aerial view of Opovo; 14) Aerial view of Dobrica; 15) Aerial view of From Debeljača to Crepaja view; 16) Nikolinci Sentinel-2 image FCC 8-3-2; 17) Lokve Sentinel-2 image FCC 8-4-3; 18) Aerial view of MBA tell at Židovar. 19) A) Oblique aerial photograph of Kacarevo; B) Georeferenced and rectified version of the aerial photograph set on the contemporary land divisions; C) Interpretation and map of all features visible on the aerial photograph of Kacarevo. Fig S2.1 Map by Marta Estanqueiro. Data by Darja Grosman. Basemap hillshade derived from ALOS DSM AW3D30 reprinted from https://www.eorcJaxaJp/ALOS/en/dataset/aw3d30/aw3d30_e.htm under a CC BY license, with permission from JAXA -Japan Aerospace Exploration Agency, original copyright 2023. Graphs plotted using R 4.2.0. Photographs S2.2–15 and 18 by Barry Molloy and Darja Grosman. Sentinel 2 images S2.16 and S2.17 defined by Marta Estanqueiro. Fig S2.19 photographs and drawings by Darja Grosman.
(PDF)

S3 File KMZ file of tumuli identified in Banat through remote prospection (File compiled by D. Grosman with contributions from D. Jovanović). (PDF)

S4 File Illustration of ceramic finds from the sites of Gradište Iđoš, Novo Miloševo 3, Novo Miloševo 4, Matejski Brod, Novi Bečej, Novi Bečej 2, Bašaid 3, Kumane 2, Melenci 4, Srpski Itebej, Novi Itebej, Melenci 7, Jankov Most 2, Klek, Žitište, Zrenjanin 4, Klek 2, Sečanj, Zrenjanin, Boka, Jarkovac, Dobrica, Uzdin, Uzdin 2, Sakule 2, Lokve, Idvor, Baranda, Sakule, Crepaja, Sefkeri, Sefkerin 2, Kačarevo, Glogonj 2, Jabuka, Glogonj, Kačarevo, Pančevo, Mramorak, Mramorak 2, Bavanište, Bavanište 2, Bavanište 3 and Jaša Tomić. All drawings by Dragan Jovanović.
(KML)

S1 Table Contextual, species and laboratory data for samples with 14C dates. (XLSX)
