# Supplementary material for: Resilience, innovation and collapse of settlement networks in later Bronze Age Europe: New survey data from the southern Carpathian Basin
Source: PLoS One. 2023 Nov 10;18(11):e0288750. doi: 10.1371/journal.pone.0288750 (PMC10637690; doi:10.1371/journal.pone.0288750)
Supplement: S2 File — 1) Record of flight track for taking aerial photographs documented using GPS for synchronising photographs and locations; 2) Extensive, low-lying drainage network and localised flooding in aerial perspective in March 2020 with archaeological site of Baranda in the margin of the left foreground and the modern town of Baranda in left nearer background; 3) Aerial view of Kačarevo; 4) Aerial view of Bavanište 2; 5) Aerial view of Mramorak; 6) Aerial view of Pančevo 2—Stari Tamiš; 7) Aerial view of Jabuka; 8) Aerial view of Bavanište; 9) Aerial view of Crepaja, 10) Aerial view of Debeljača; 11) Aerial view of Sakule. Central enclosure is centre field, small “citadel” enclosure is in left background. Footprints from pedestrian survey grid visible within enclosures; 12) Aerial view of Sefkerin; 13) Aerial view of Opovo; 14) Aerial view of Dobrica; 15) Aerial view of From Debeljača to Crepaja view; 16) Nikolinci Sentinel-2 image FCC 8-3-2; 17) Lokve Sentinel-2 image FCC 8-4-3; 18) Aerial view of MBA tell at Židovar. 19) A) Oblique aerial photograph of Kacarevo; B) Georeferenced and rectified version of the aerial photograph set on the contemporary land divisions; C) Interpretation and map of all features visible on the aerial photograph of Kacarevo. Fig S2.1 Map by Marta Estanqueiro. Data by Darja Grosman. Basemap hillshade derived from ALOS DSM AW3D30 reprinted from https://www.eorcJaxaJp/ALOS/en/dataset/aw3d30/aw3d30_e.htm under a CC BY license, with permission from JAXA -Japan Aerospace Exploration Agency, original copyright 2023. Graphs plotted using R 4.2.0. Photographs S2.2–15 and 18 by Barry Molloy and Darja Grosman. Sentinel 2 images S2.16 and S2.17 defined by Marta Estanqueiro. Fig S2.19 photographs and drawings by Darja Grosman. (PDF) [file pone.0288750.s002.pdf]

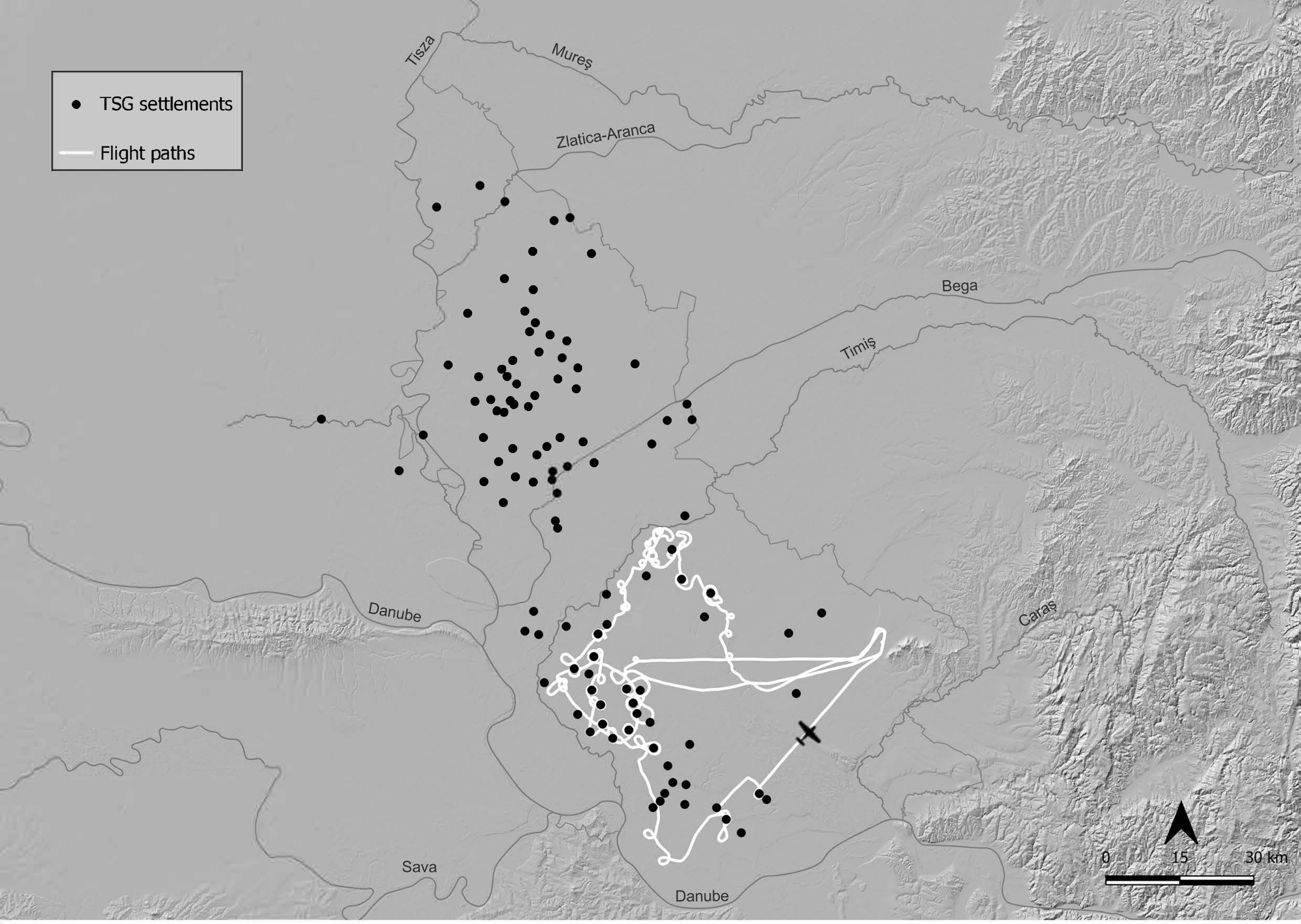

1. Record of flight track for taking aerial photographs documented using GPS for synchronising photographs and locations. Illustration by Marta Estanqueiro using data provided by Darja Grosman using basemap hillshade derived from ALOS DSM AW3D30, reprinted from https://www.eorcJaxaJp/ALOS/en/dataset/aw3d30/aw3d30_e.htm under a CC BY license, with permission from JAXA -Japan Aerospace Exploration Agency, original copyright 2023. Graphs plotted using R 4.2.0

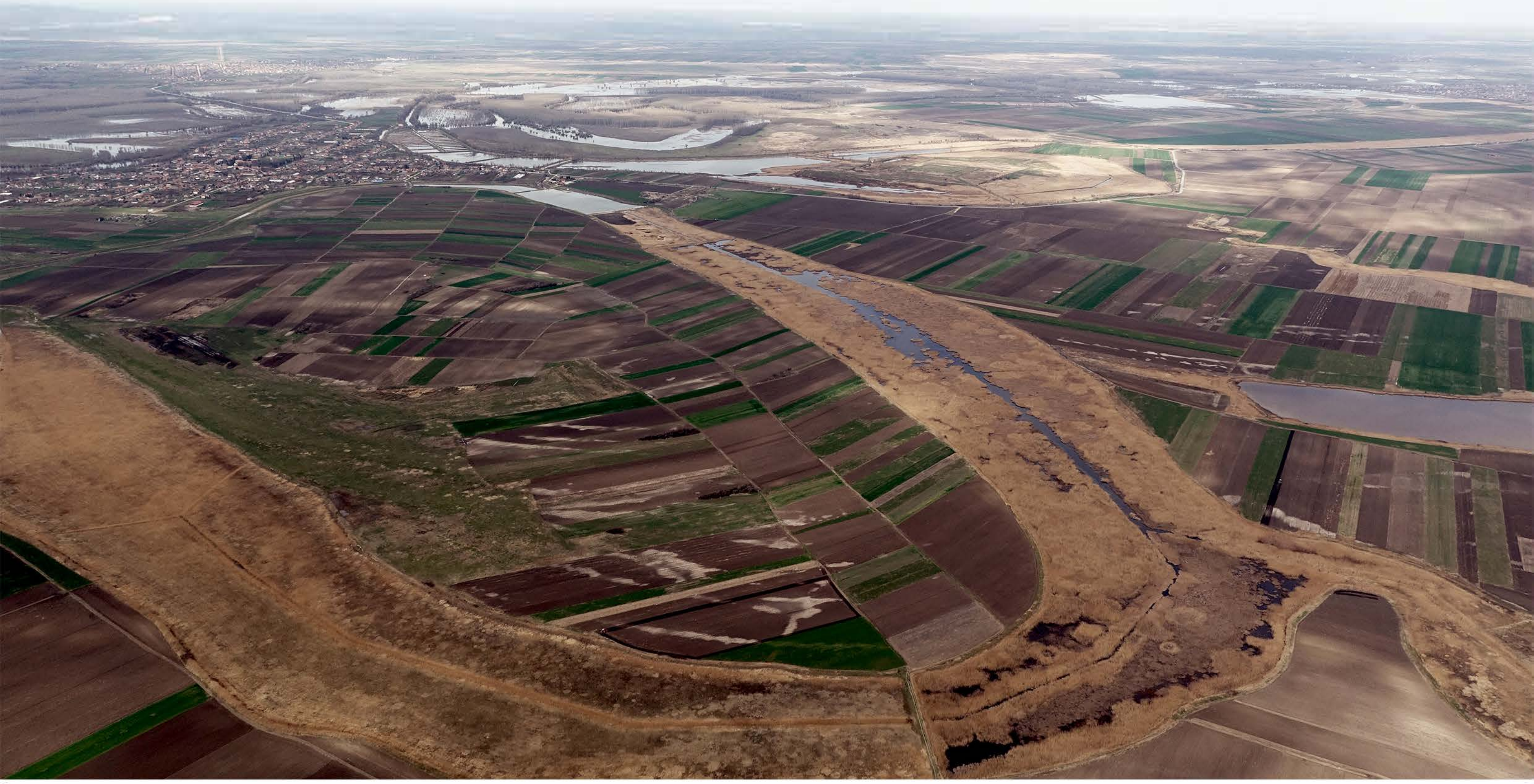

2. Extensive, low-lying drainage network and localised flooding in aerial perspective in March 2020 with archaeological site of Baranda in the margin of the left foreground and the modern town of Baranda in left nearer background.

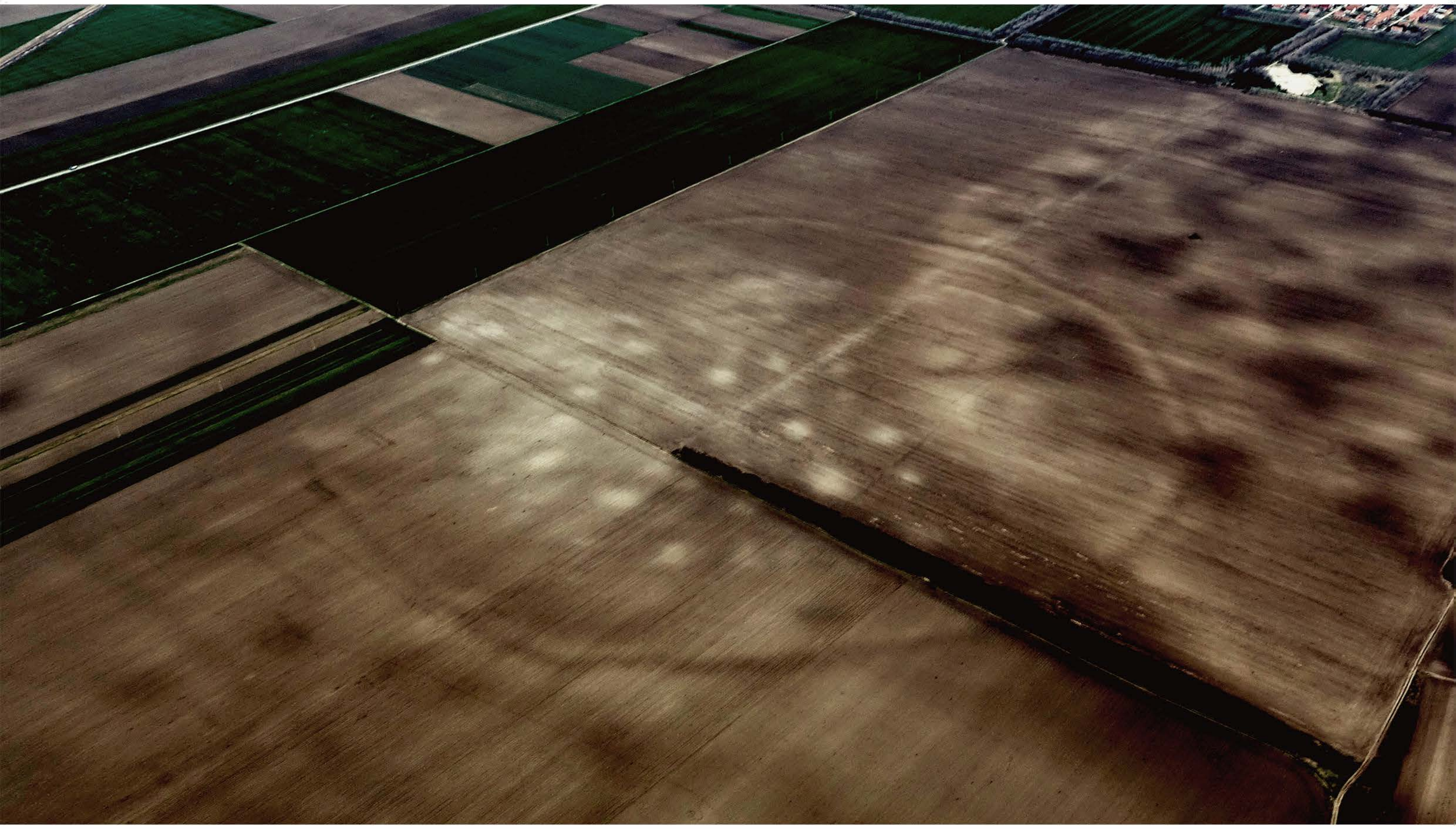

3. Aerial view of Kačarevo

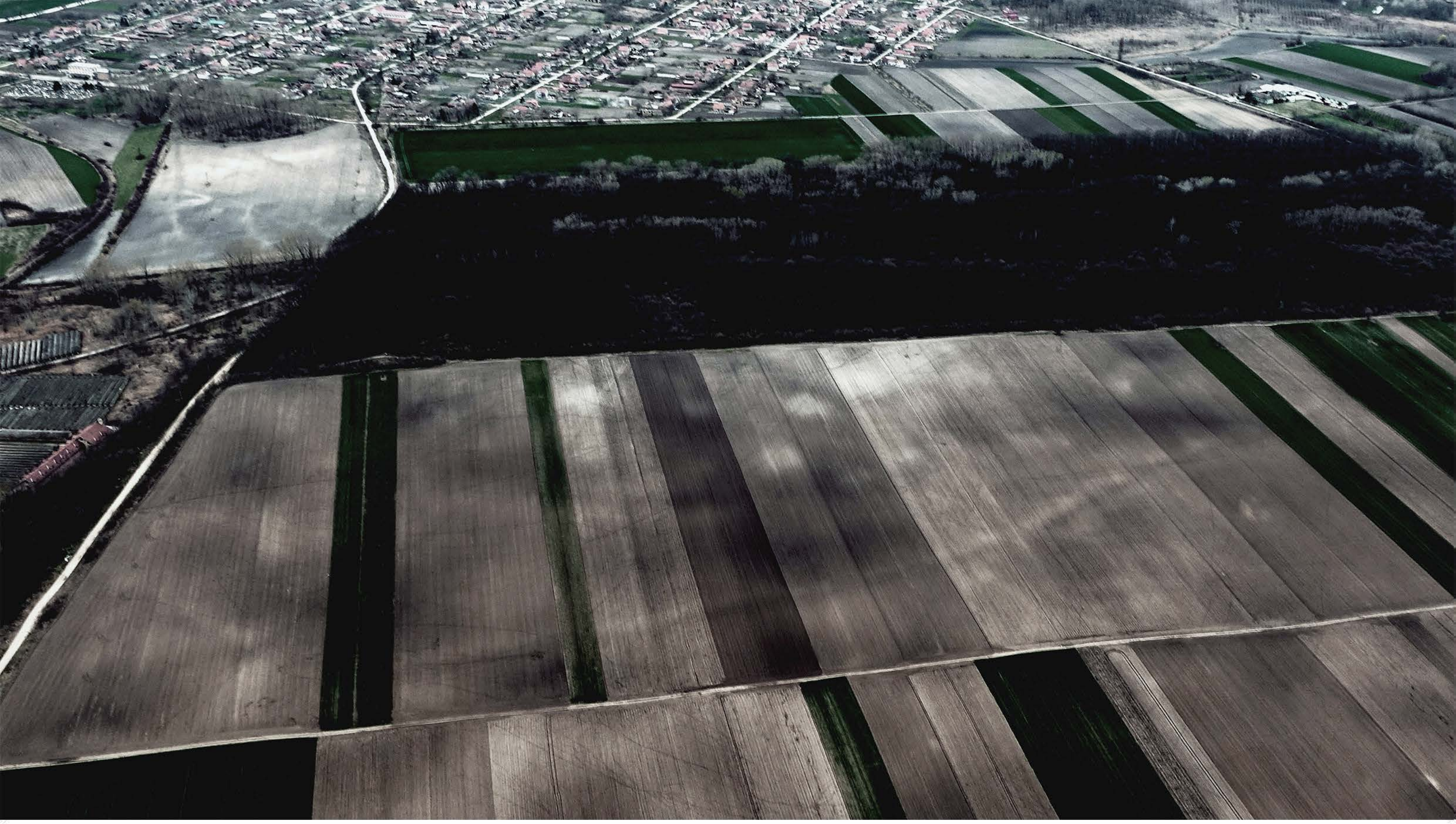

4. Aerial view of Bavanište 2

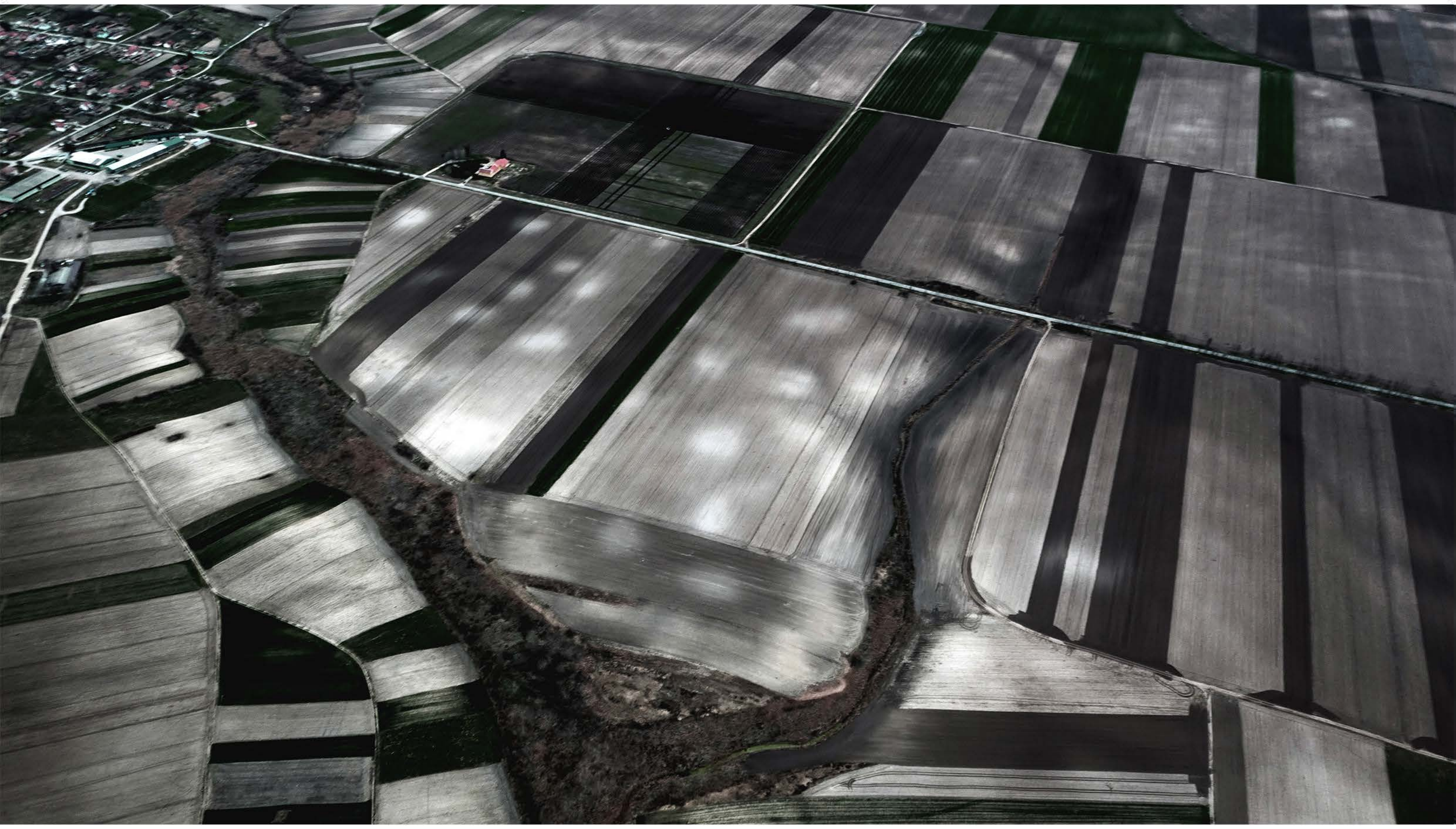

5. Aerial view of Mramorak

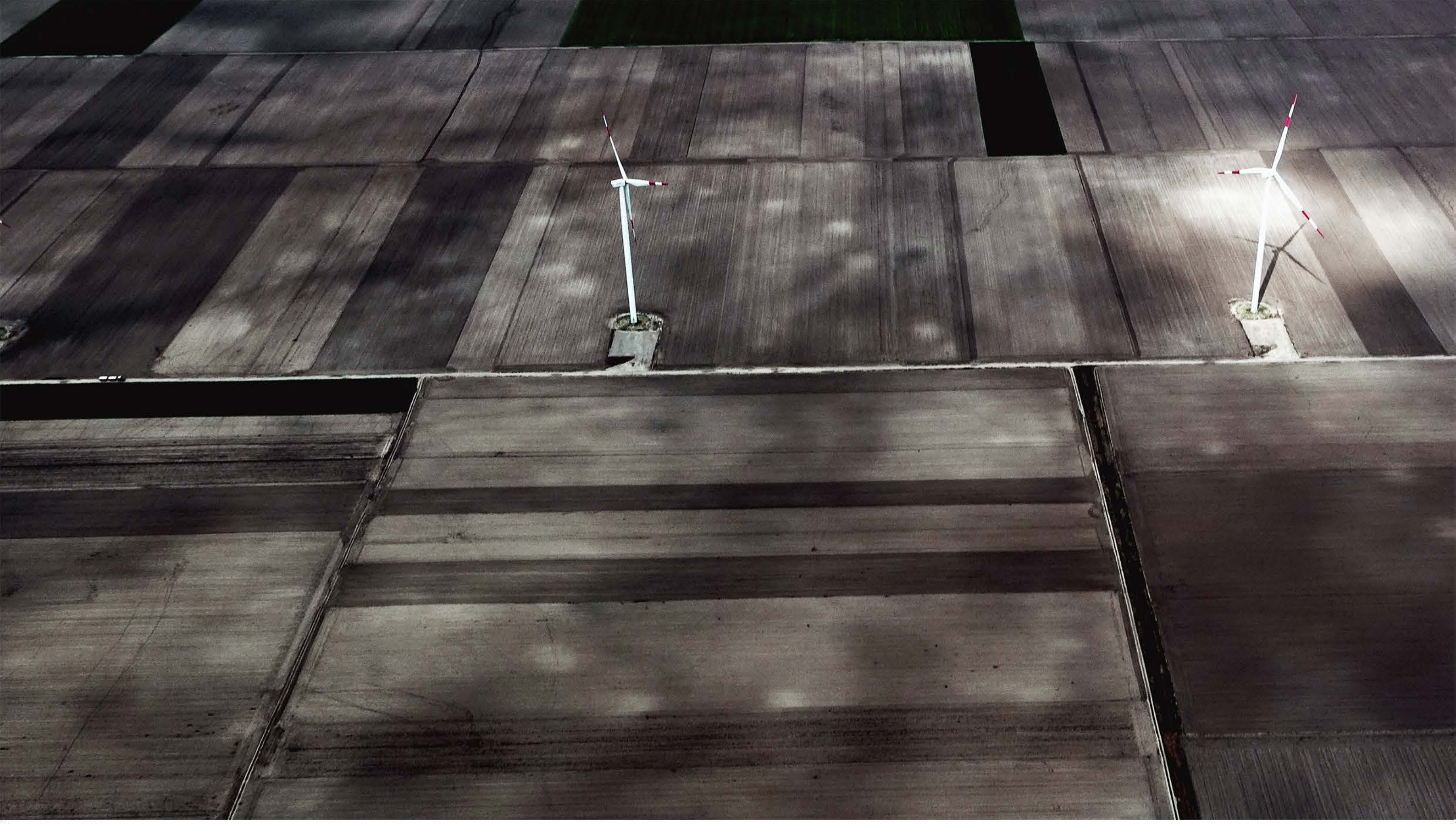

6. Aerial view of Pančevo 2 - Stari Tamiš

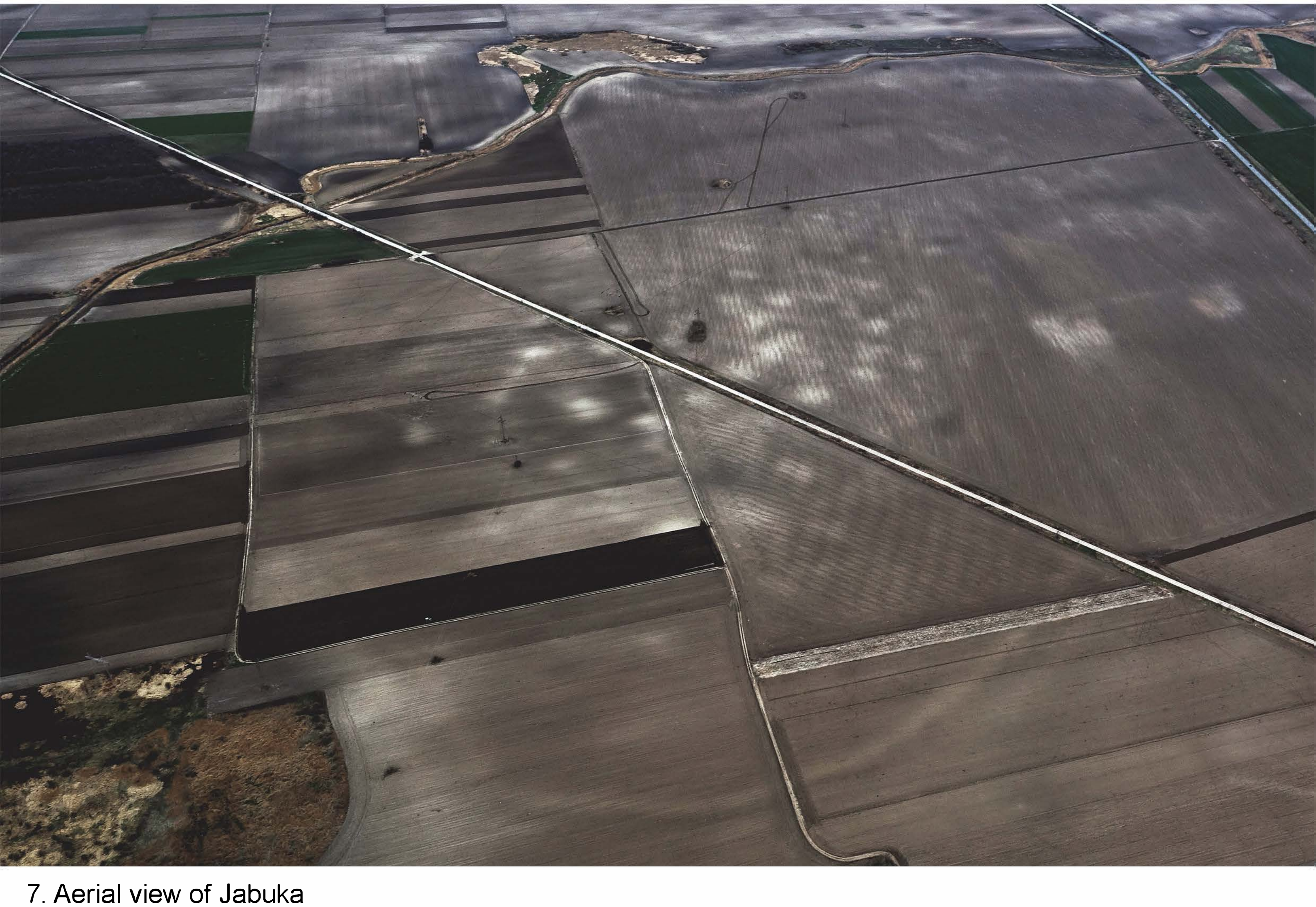

7. Aerial view of Jabuka

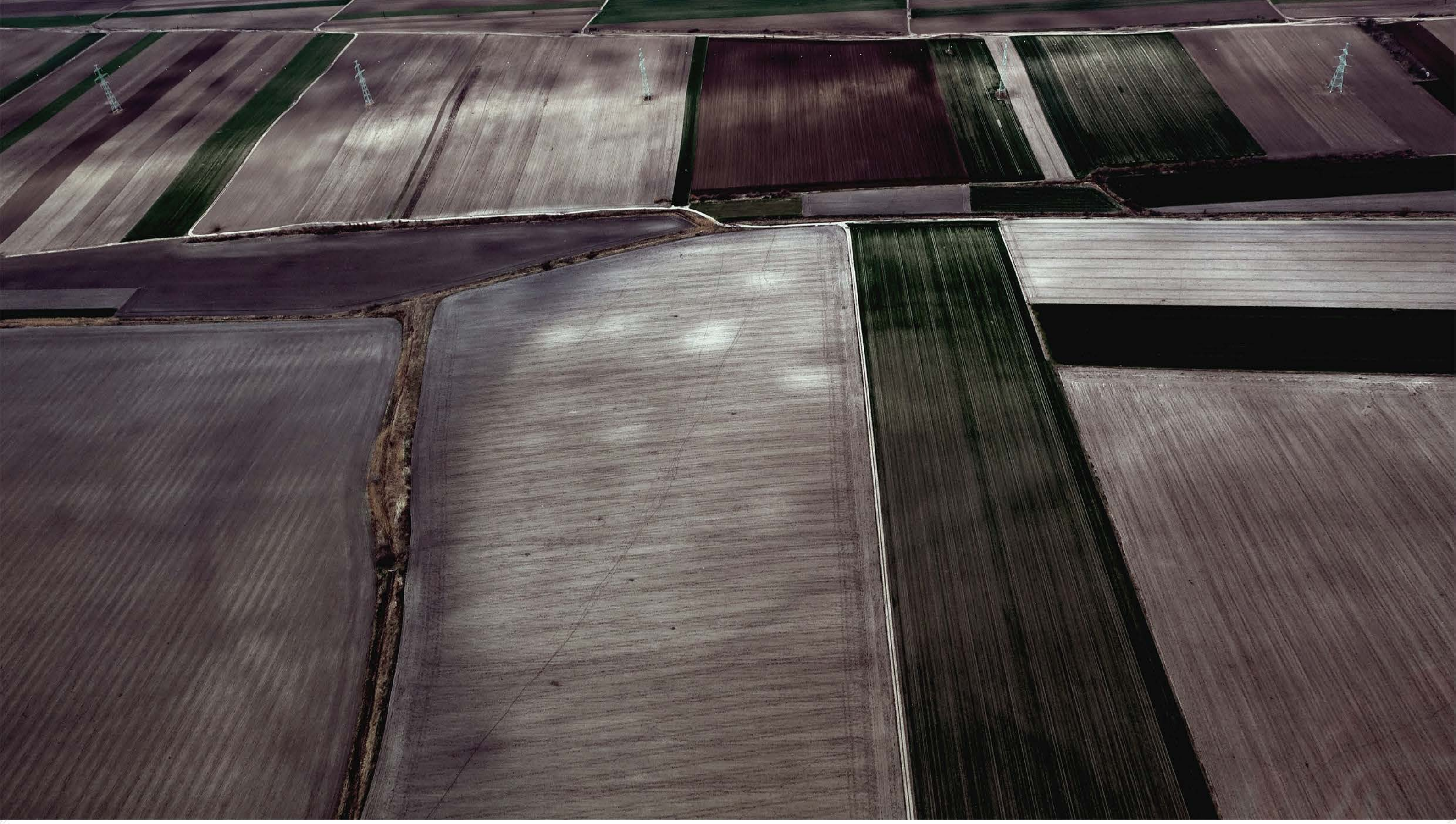

8. Aerial view of Bavanište

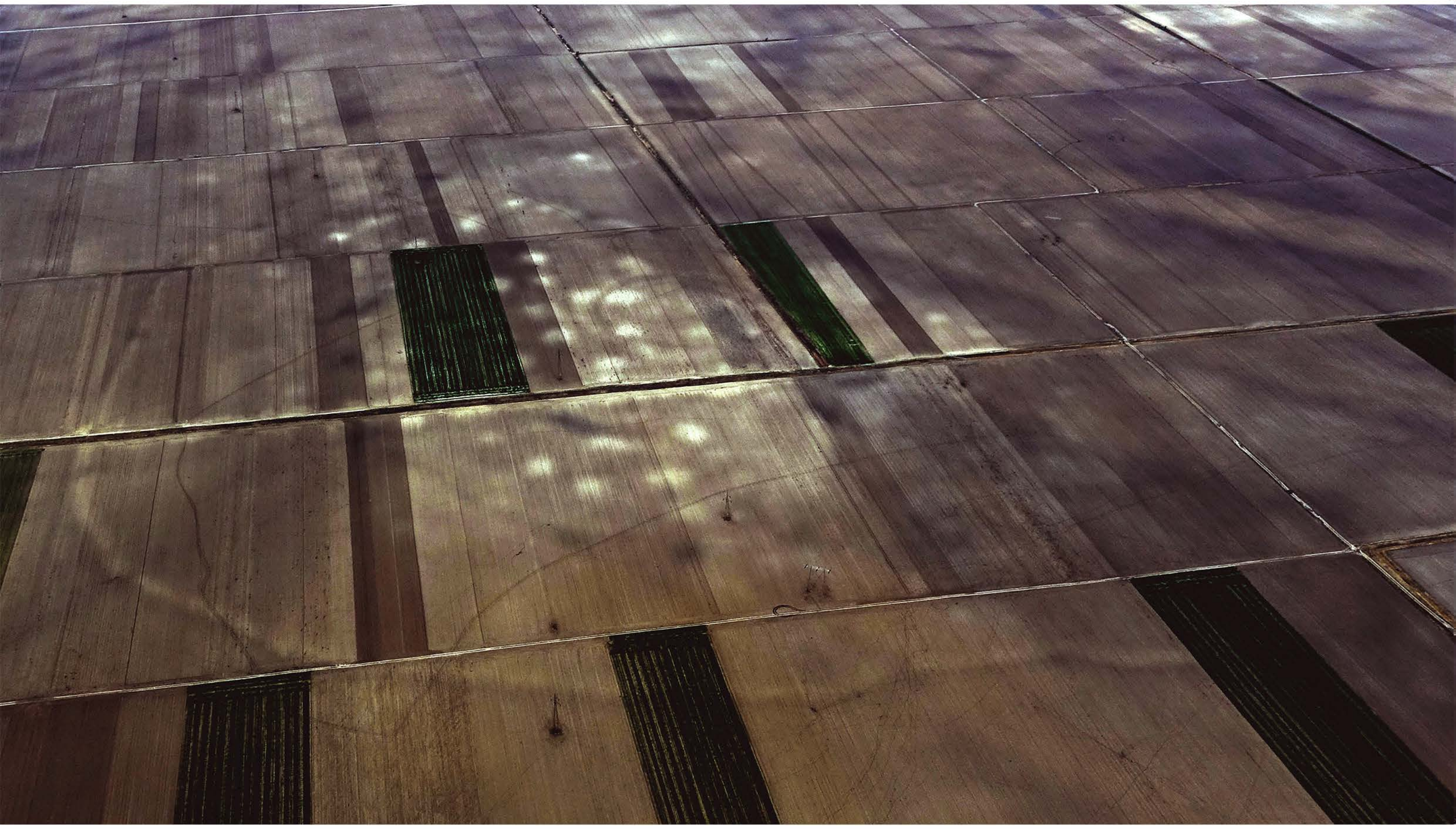

9. Aerial view of Crepaja

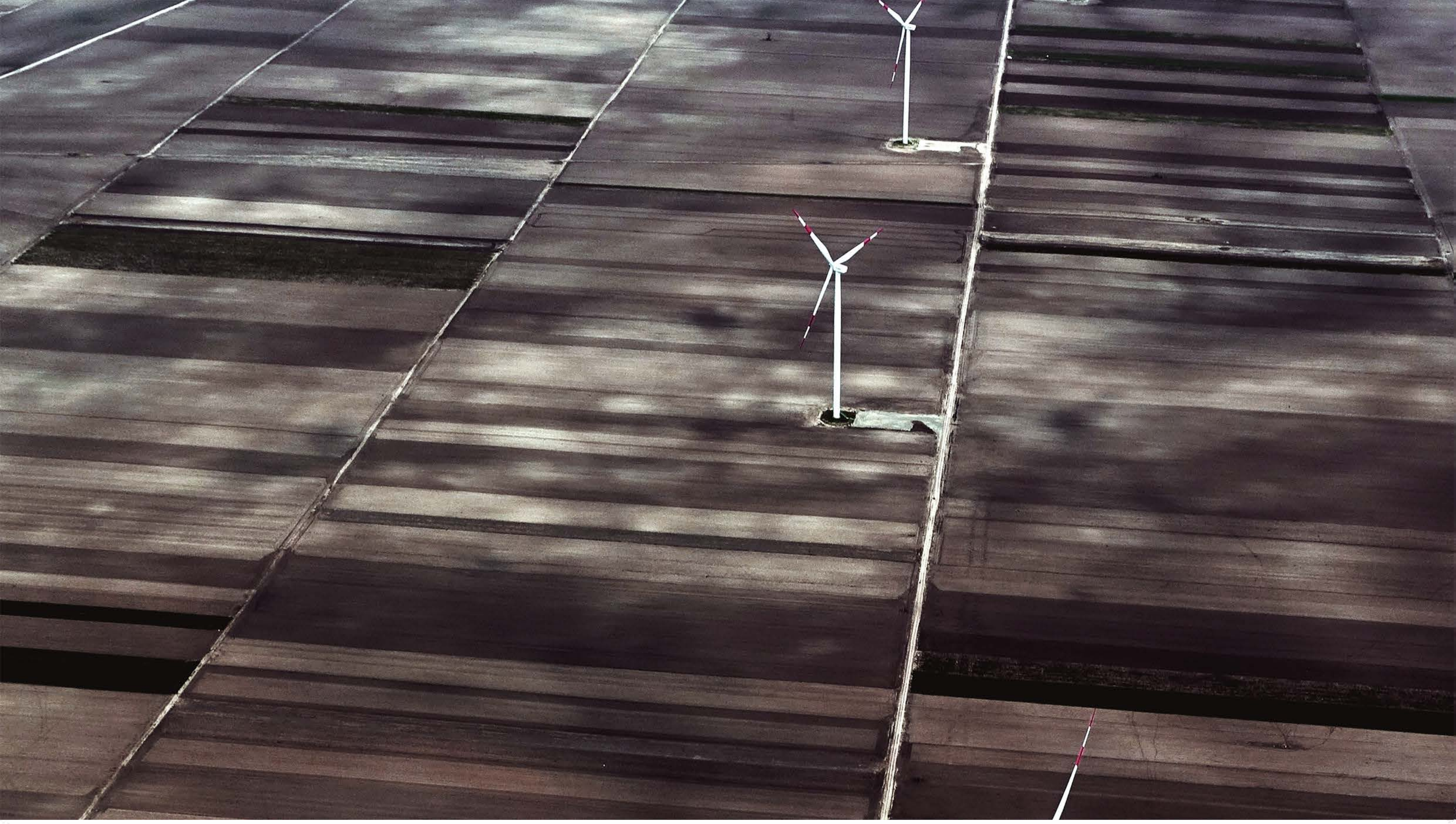

10. Aerial view of Debeljača

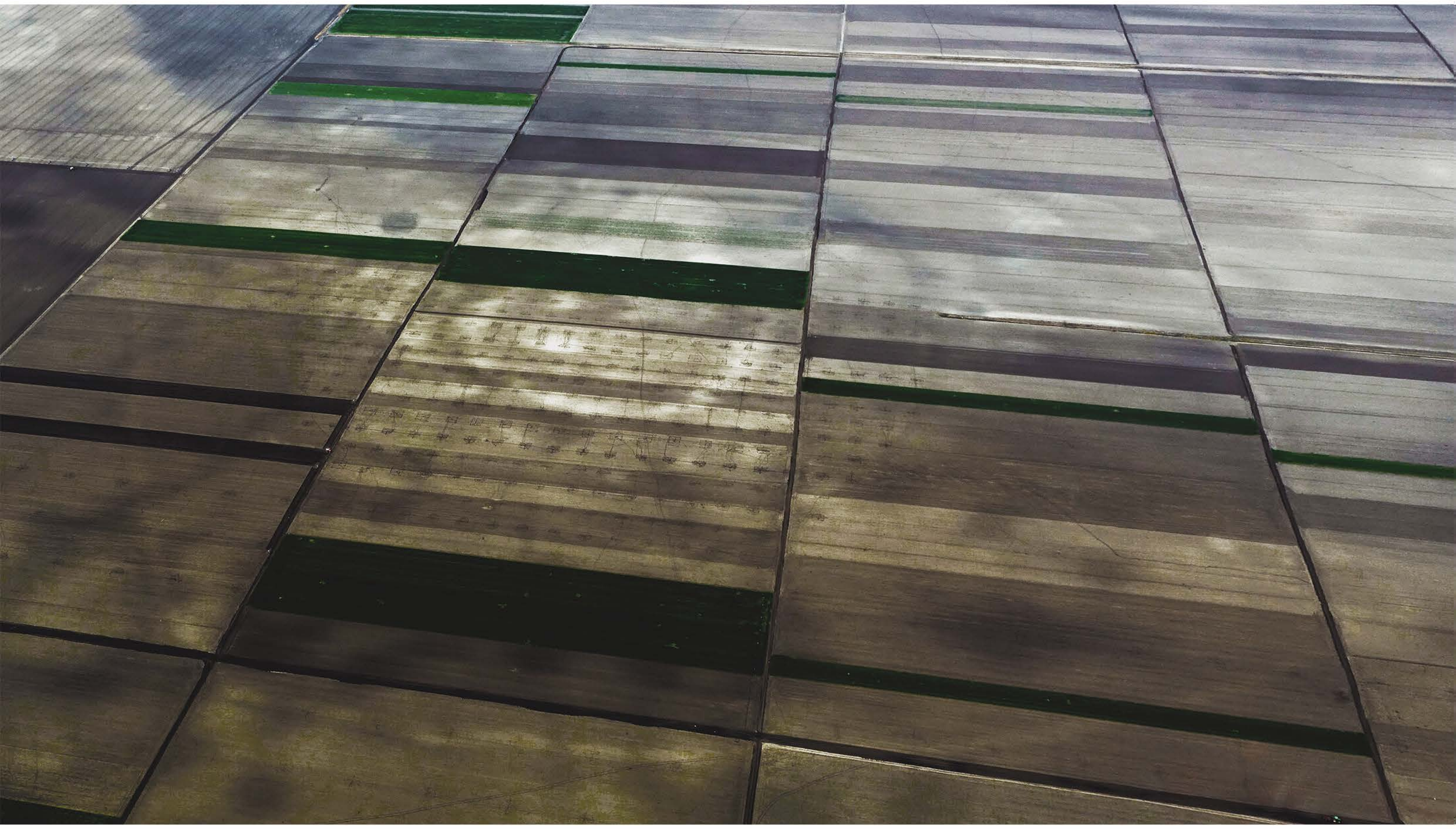

11. Aerial view of Sakule. Central enclosure is centre field, small “citadel” enclosure is in left back-ground. Footprints from pedestrian survey grid visible within enclosures

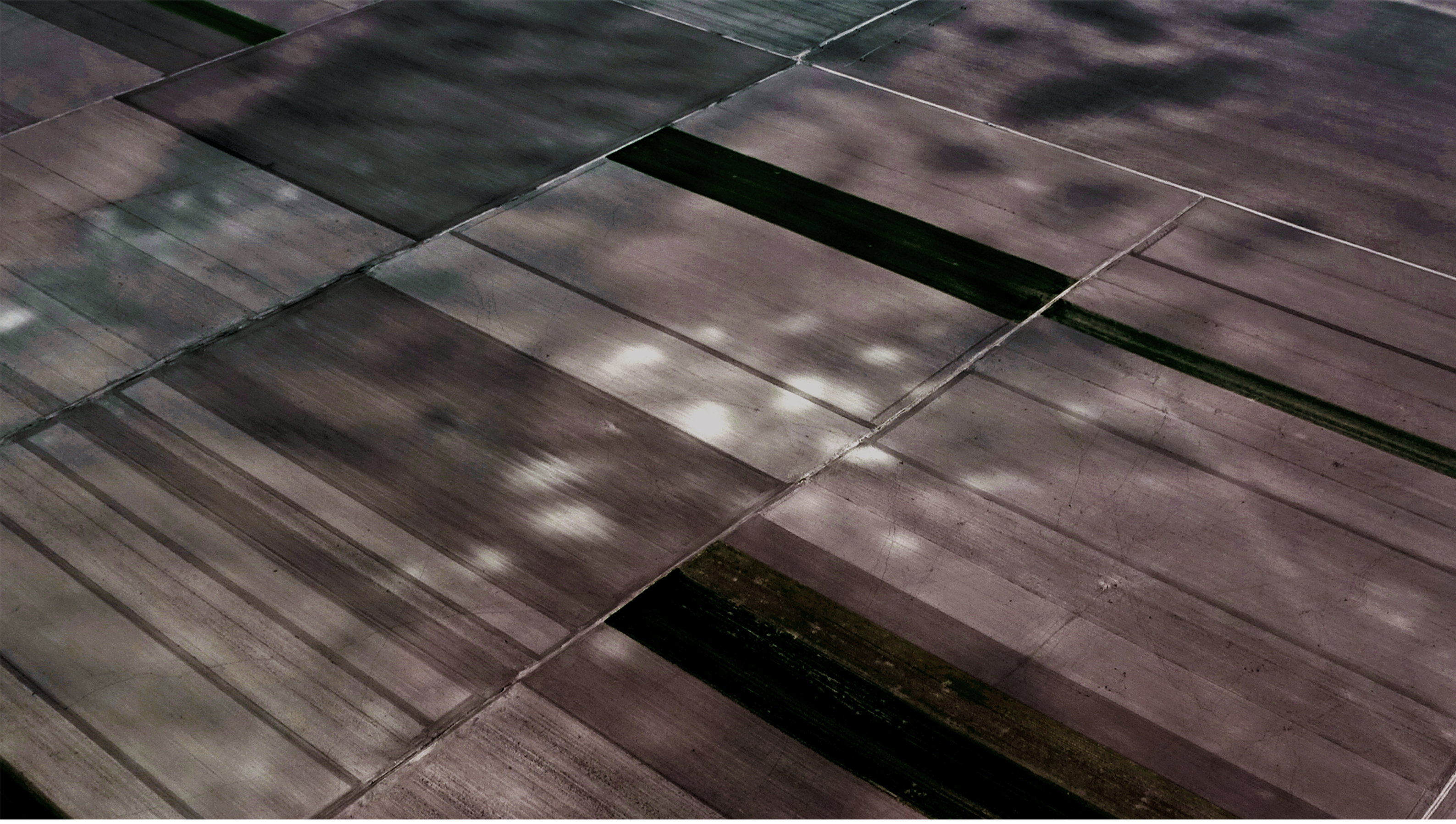
12. Aerial view of Sefkerin

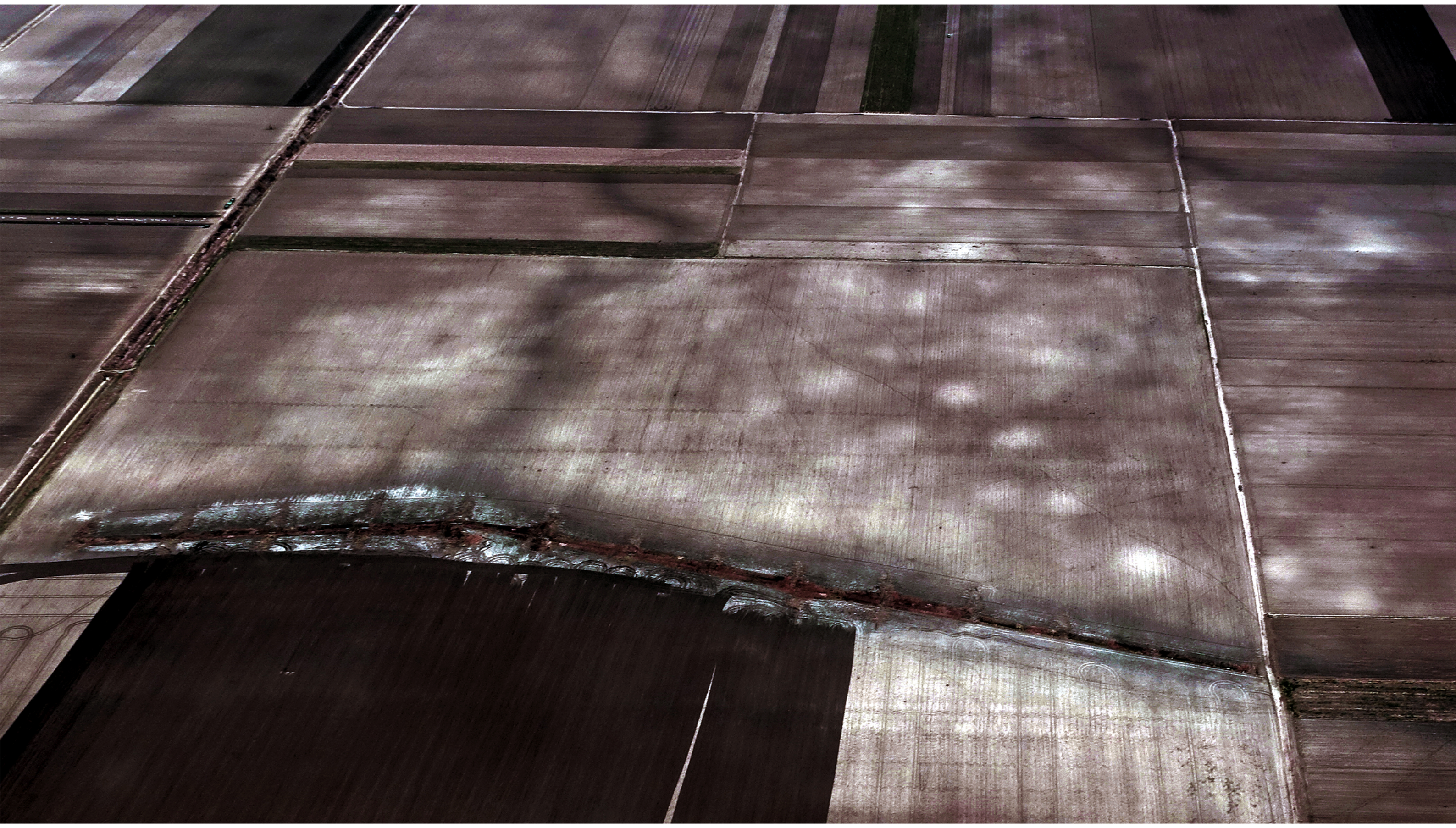
13. Opovo

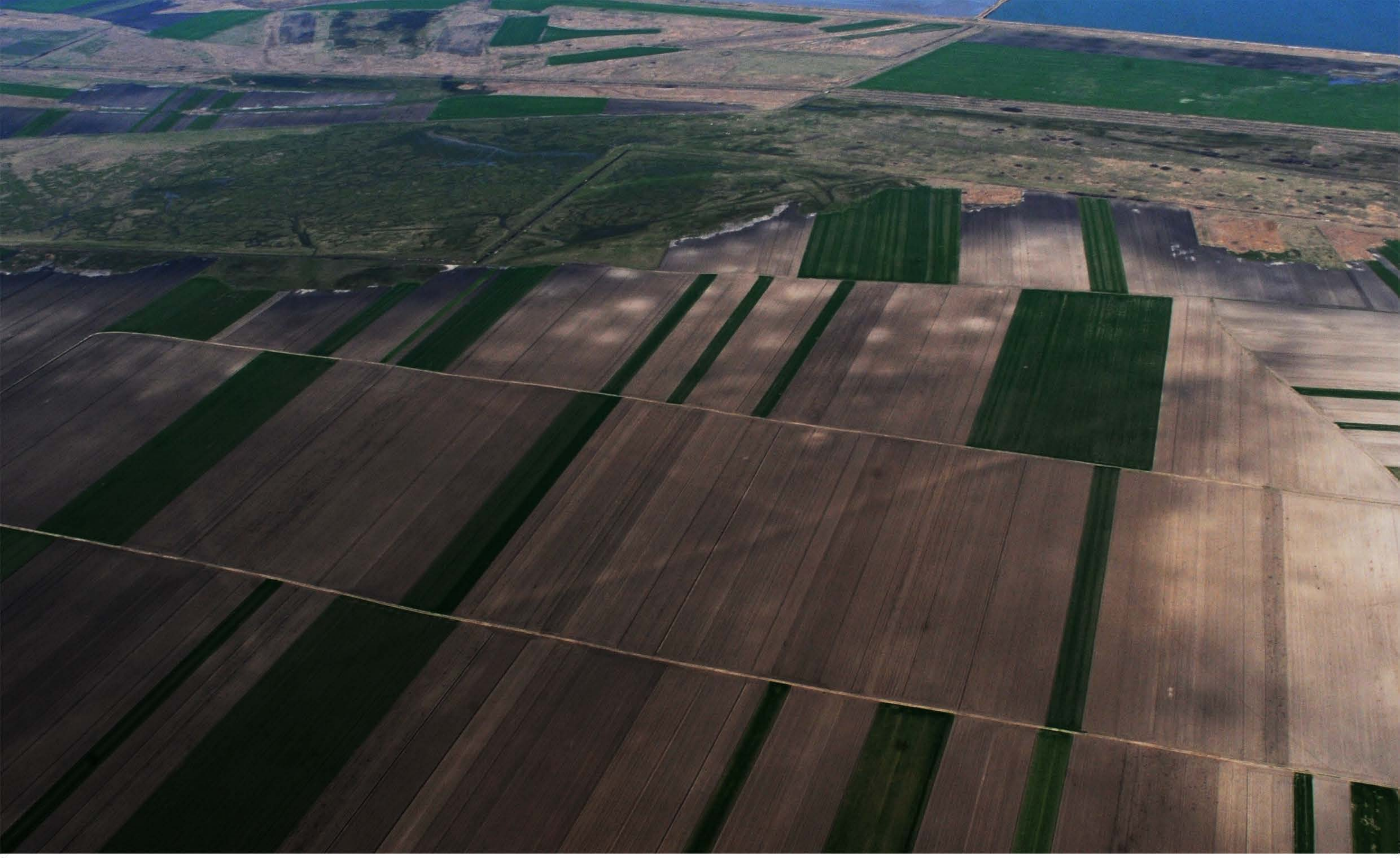

14. Aerial view of Dobrica

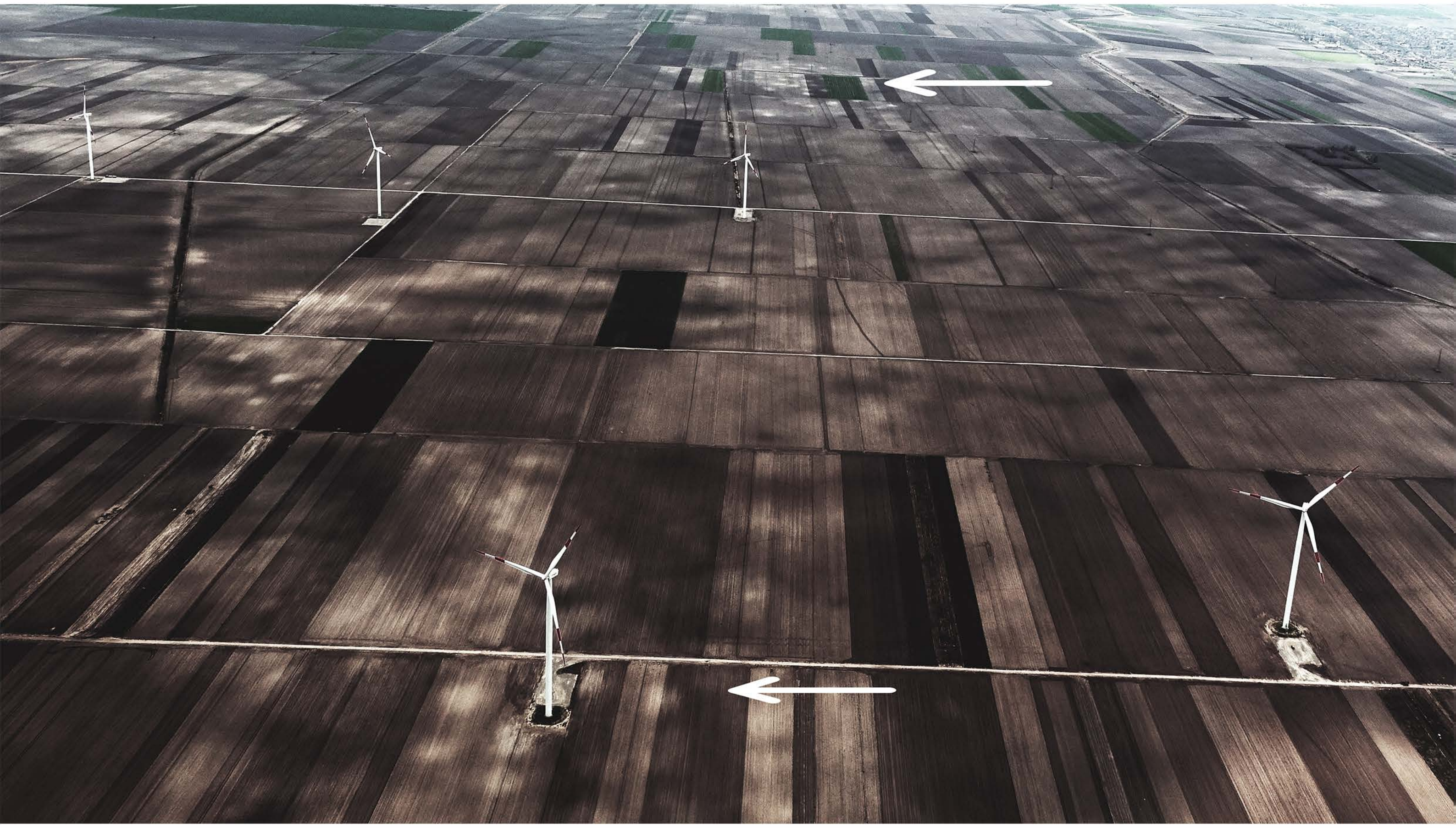

15. Aerial view from Debeljača to Crepaja

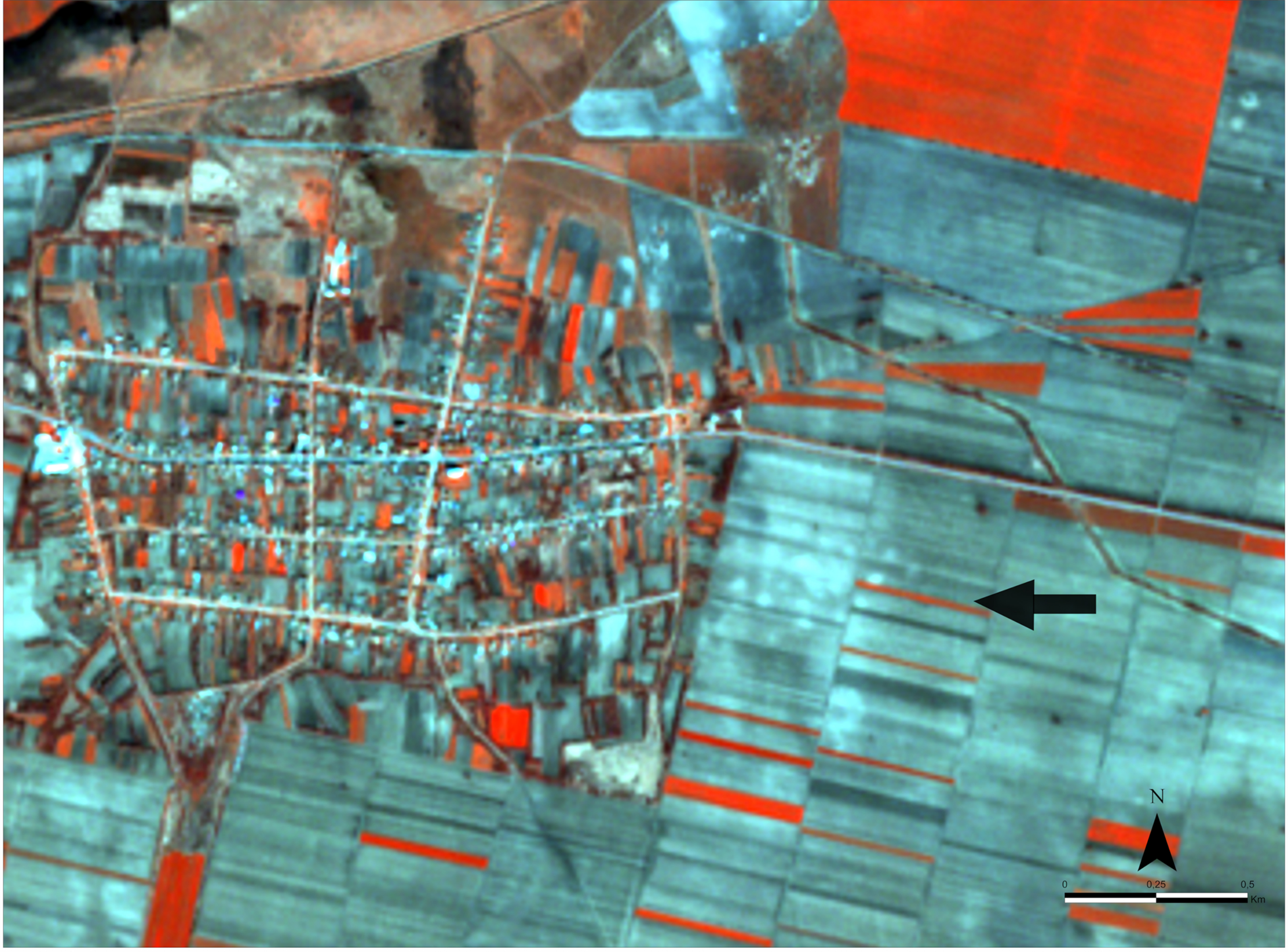

16. Nikolinci Sentinel-2 image FCC 8-3-2

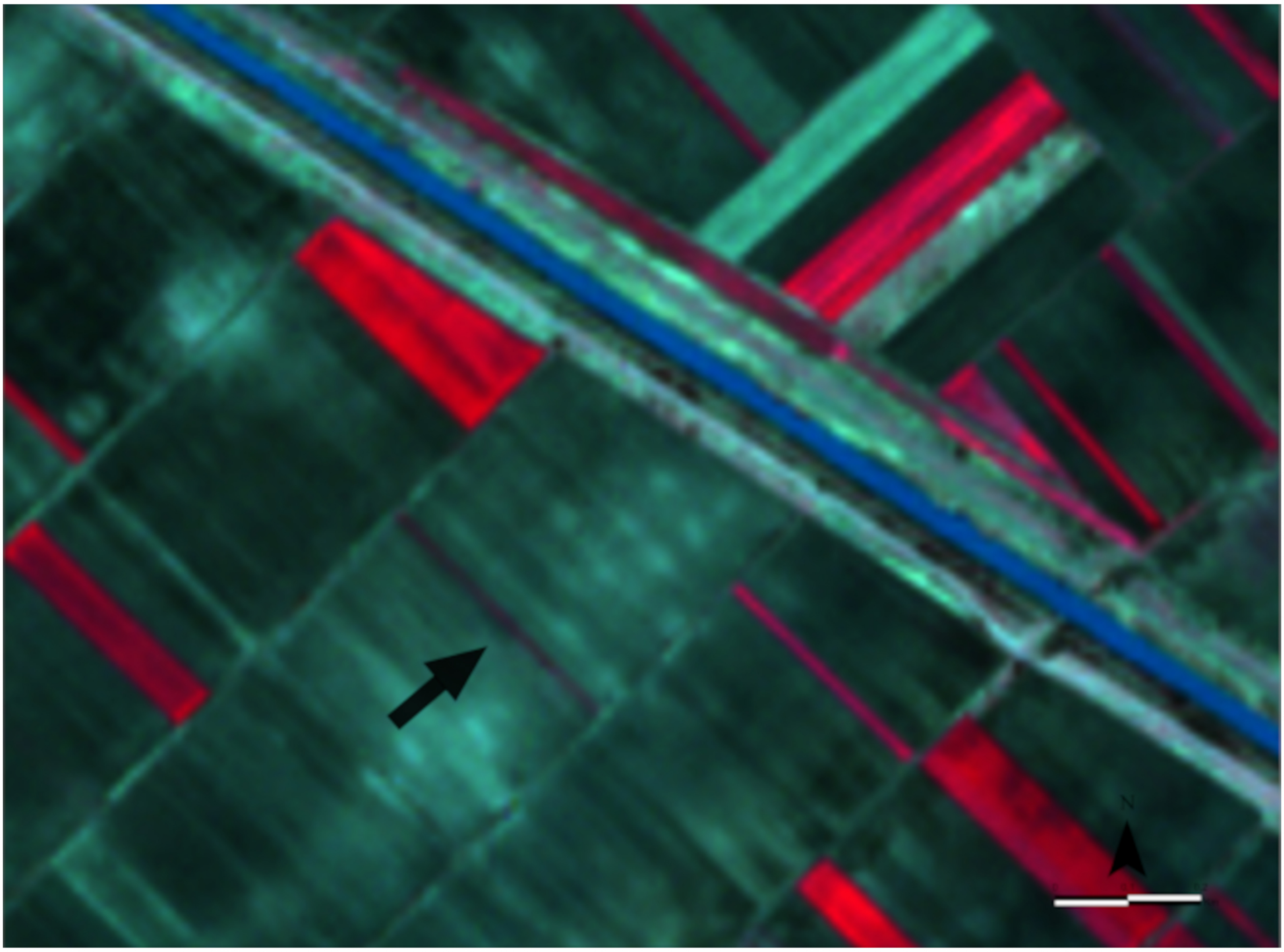

17. Lokve Sentinel-2 image FCC 8-4-3

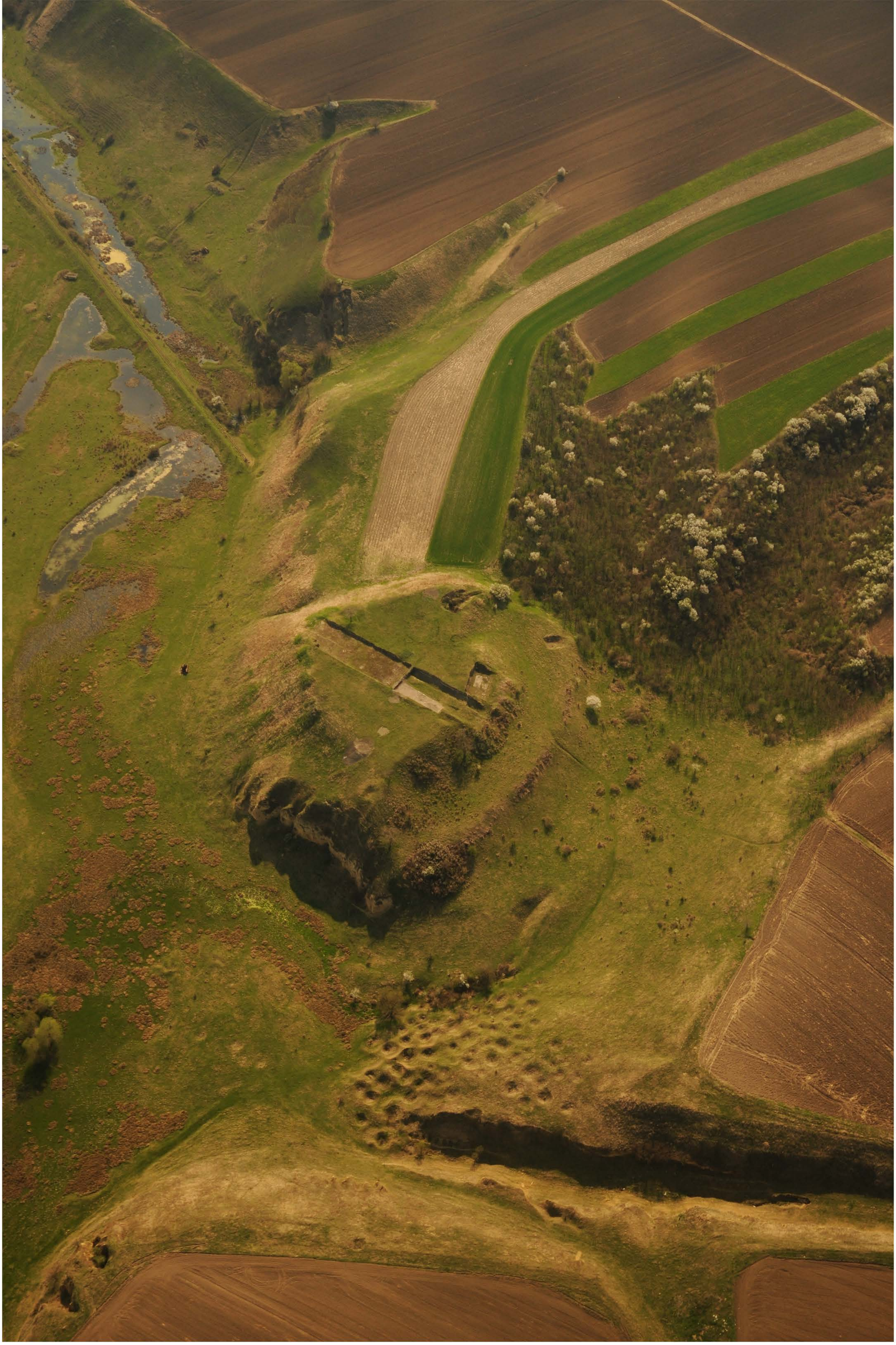

18. Aerial view of MBA tell at Židovar

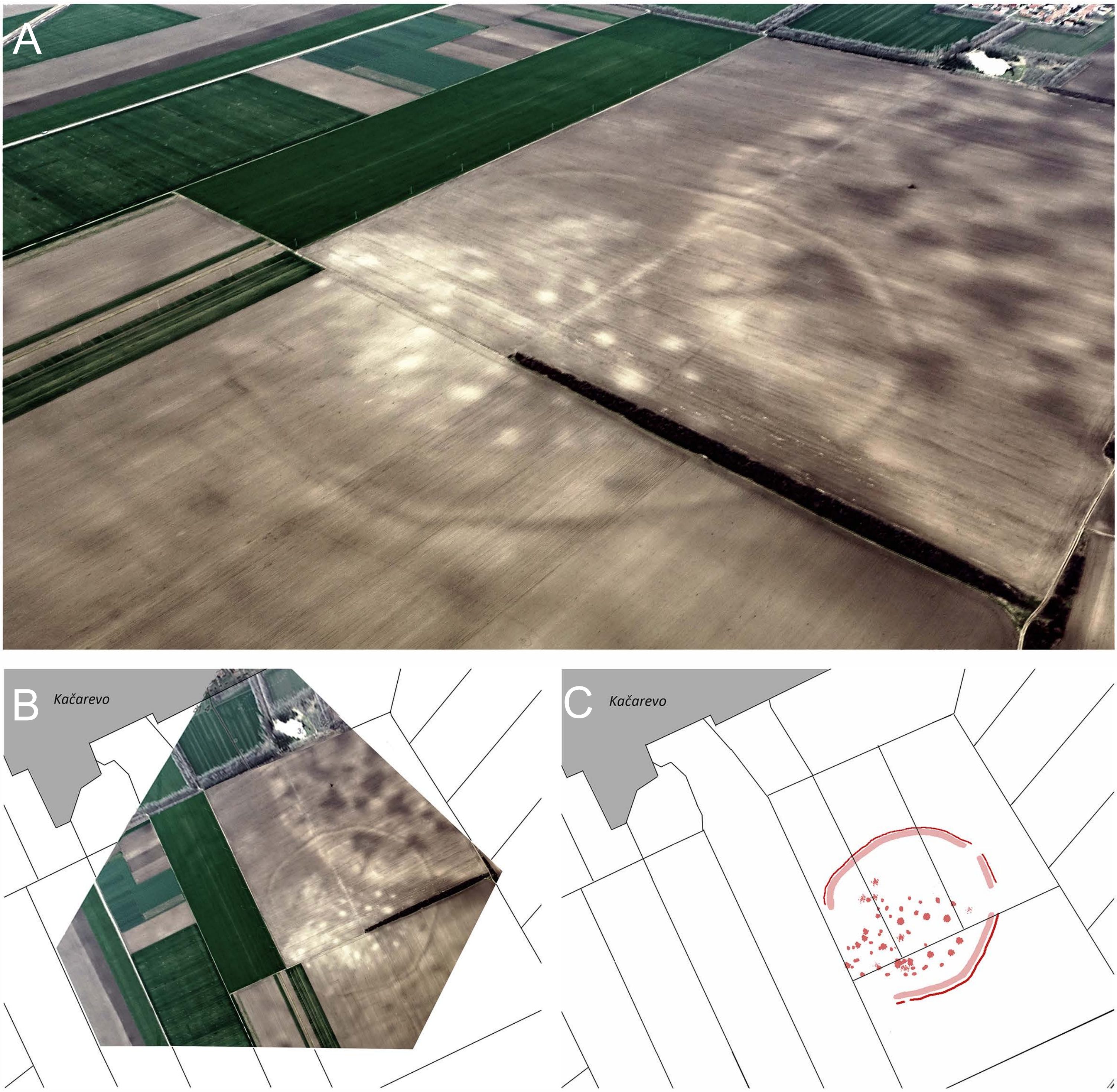

19. A) Oblique aerial photograph of Kačarevo; B) Georeferenced and rectified version of the aerial photograph set on the contempoary land divisions; C) Interpretation and map of all features visible on the aerial photograph of Kačarevo. Photographs and all illustrations by Darja Grosman.
