# Supplementary material for: Resilience, innovation and collapse of settlement networks in later Bronze Age Europe: New survey data from the southern Carpathian Basin
Source: PLoS One. 2023 Nov 10;18(11):e0288750. doi: 10.1371/journal.pone.0288750 (PMC10637690; doi:10.1371/journal.pone.0288750)
Supplement: S3 File — (PDF) [file pone.0288750.s003.pdf]

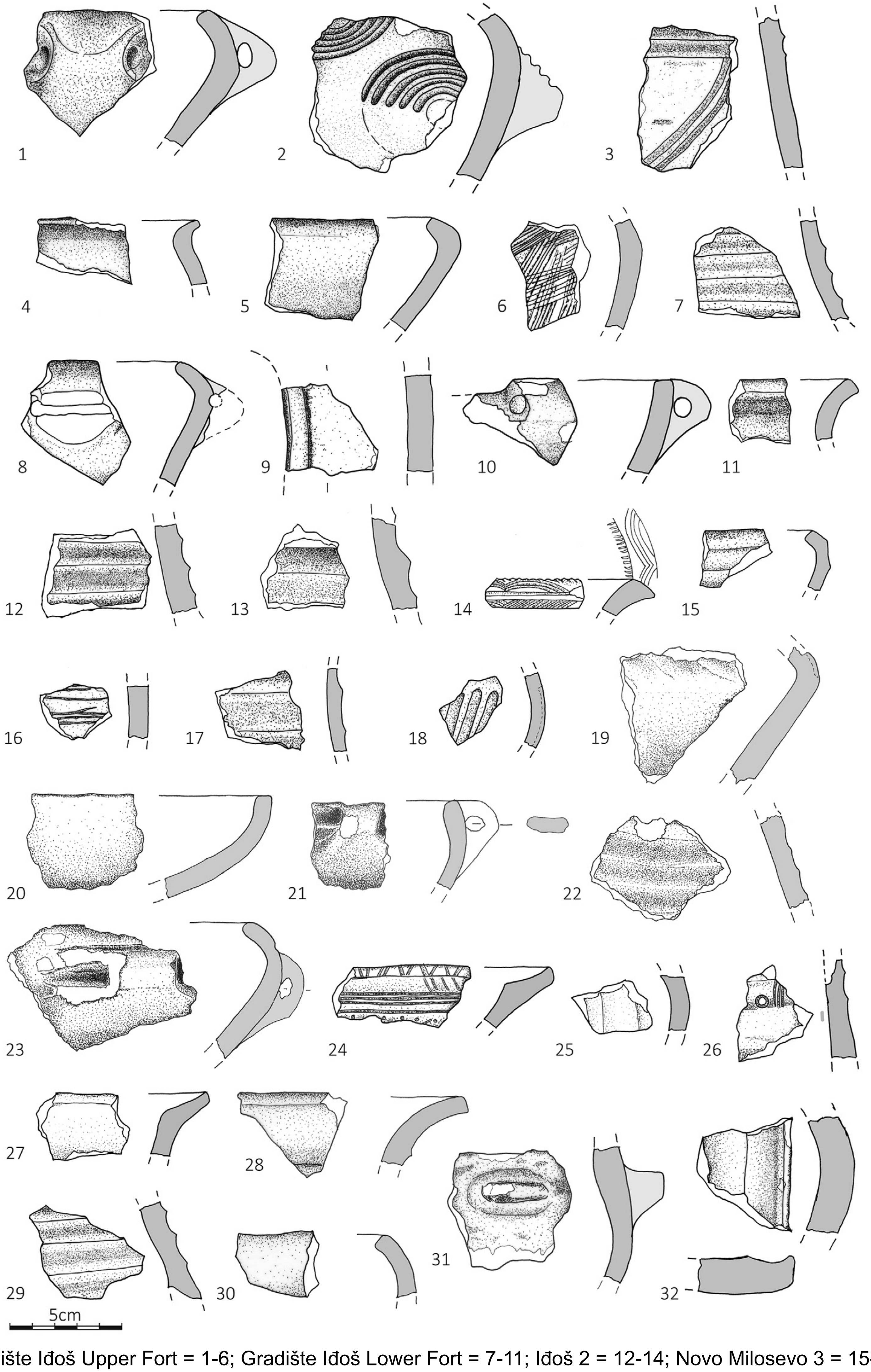

Gradište Iđoš Upper Fort = 1-6; Gradište Iđoš Lower Fort = 7-11; Iđoš 2 = 12-14; Novo Milosevo 3 = 15-18; Novo Milosevo 4 = 19-23; Matejski Brod = 24-27; Novi Bečej = 28-32.

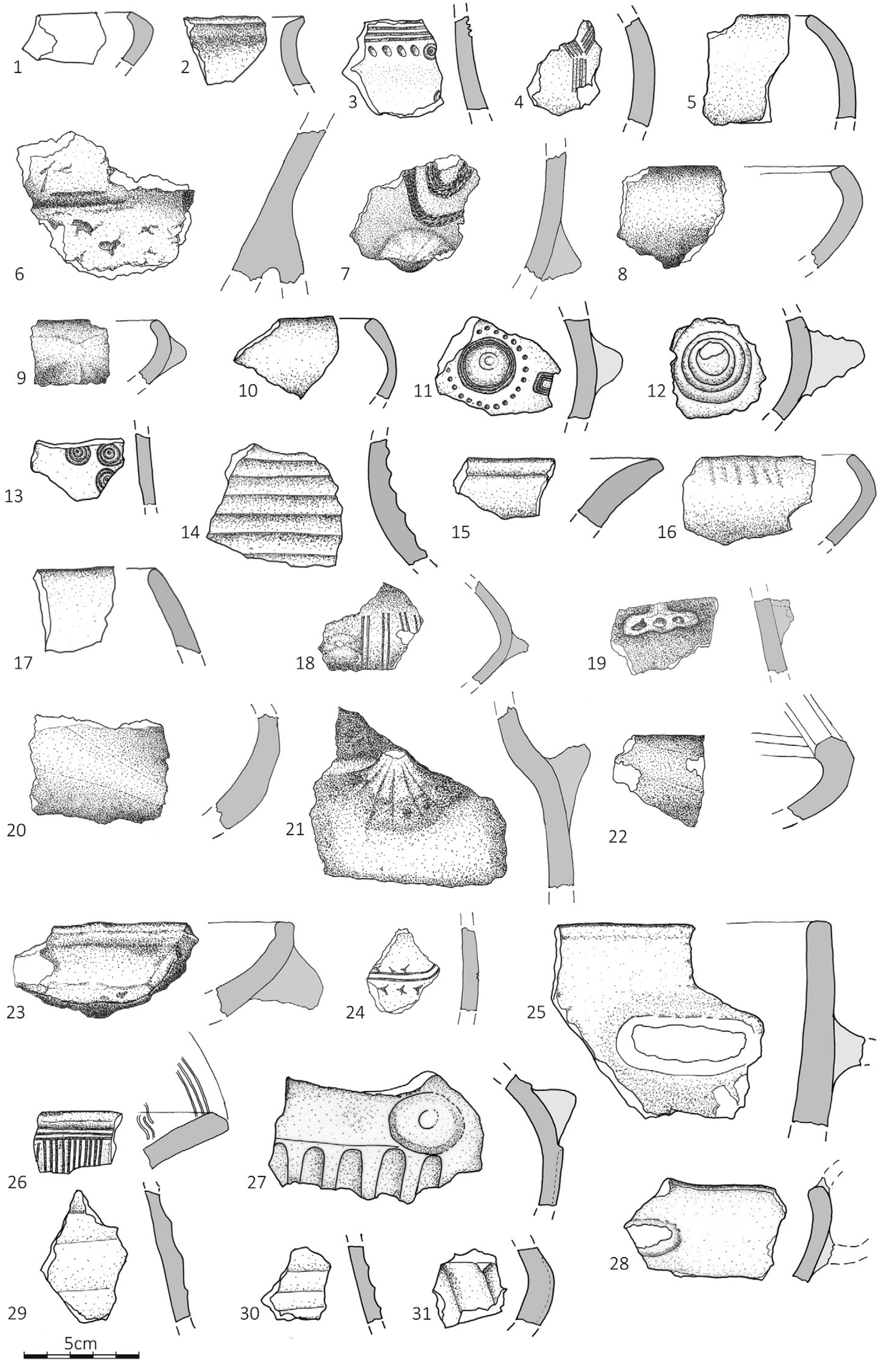

Novi Bečej 2 = 1-5; Bašaid 3 = 6-9; Kumane 2 = 10-13; Melenci 4 = 14-17; Srpski Itebej = 18-19; Novi Itebej = 20; Melenci 7 = 21-24; Jankov Most 2 = 25-28; Klek = 29-31.

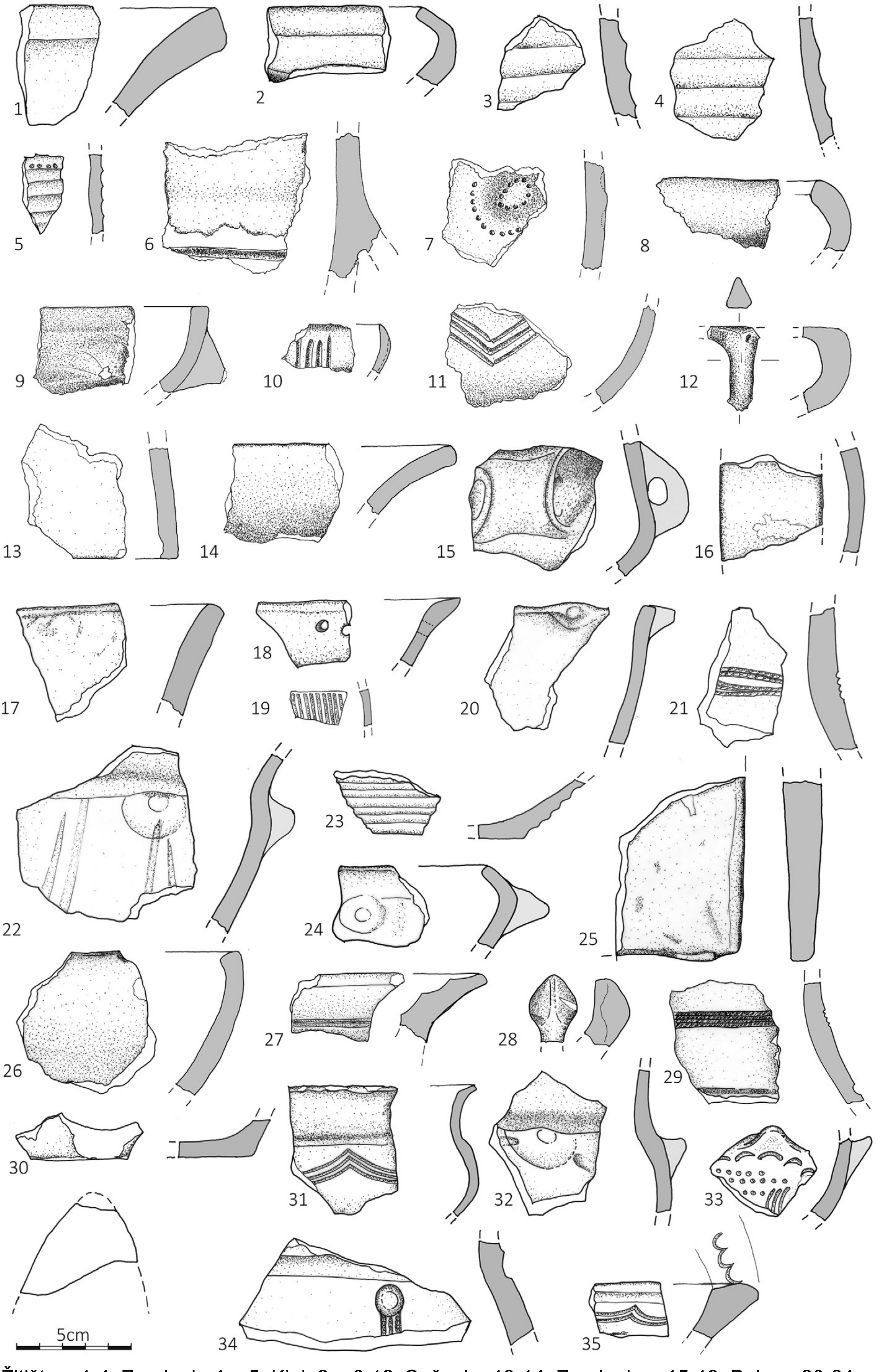

Žitište = 1-4; Zrenjanin 4 = 5; Klek 2 = 6-12; Sečanj = 13-14; Zrenjanin = 15-19; Boka = 20-24; Jarkovac = 25-29; Dobrica = 30-35

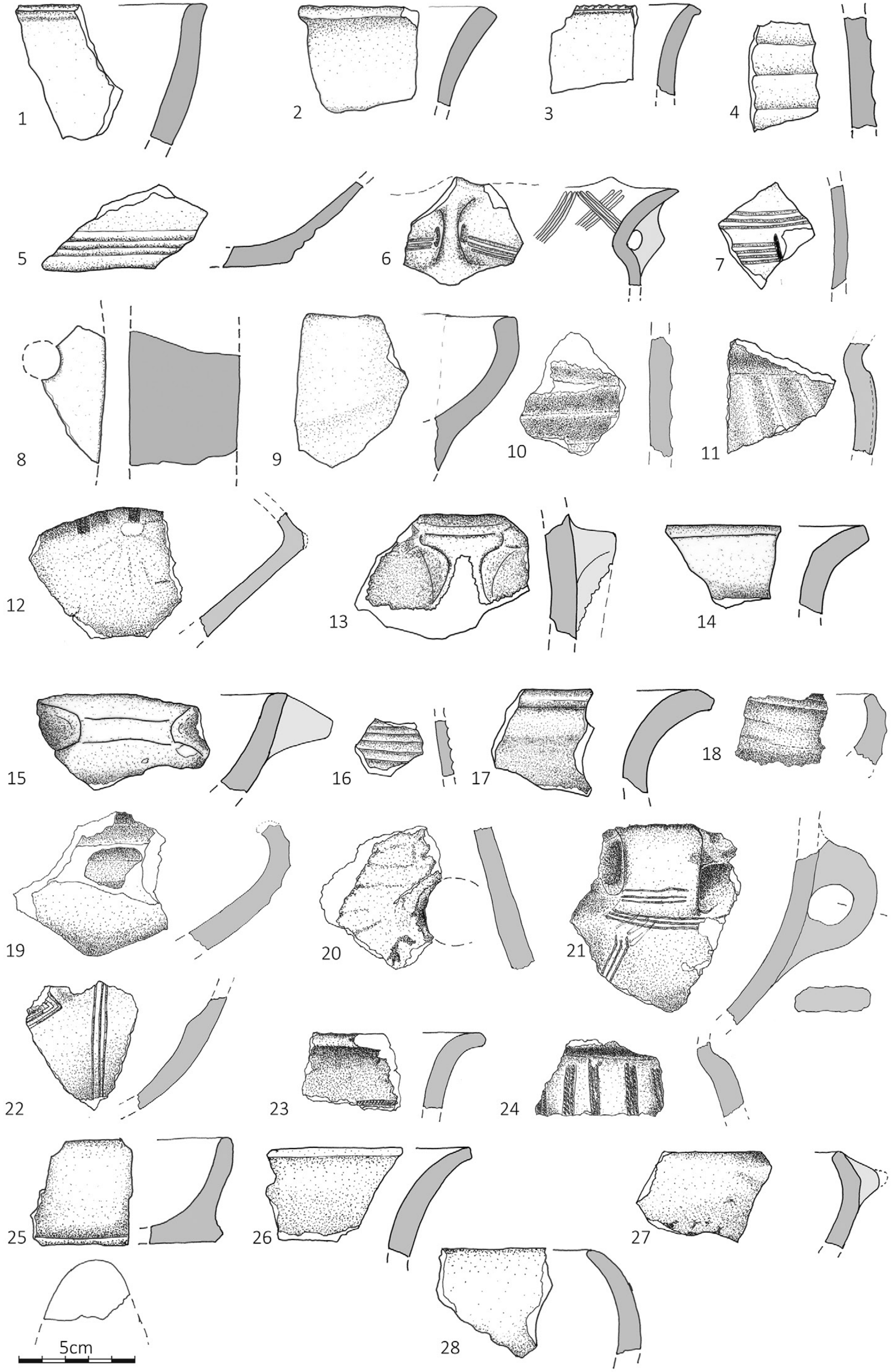

Uzdin 2 = 1-5; Uzdin = 6-9; Sakule 2 = 10; Lokve = 11-12; Idvor = 13-17; Čenta = 18-24; Baranda = 25-28

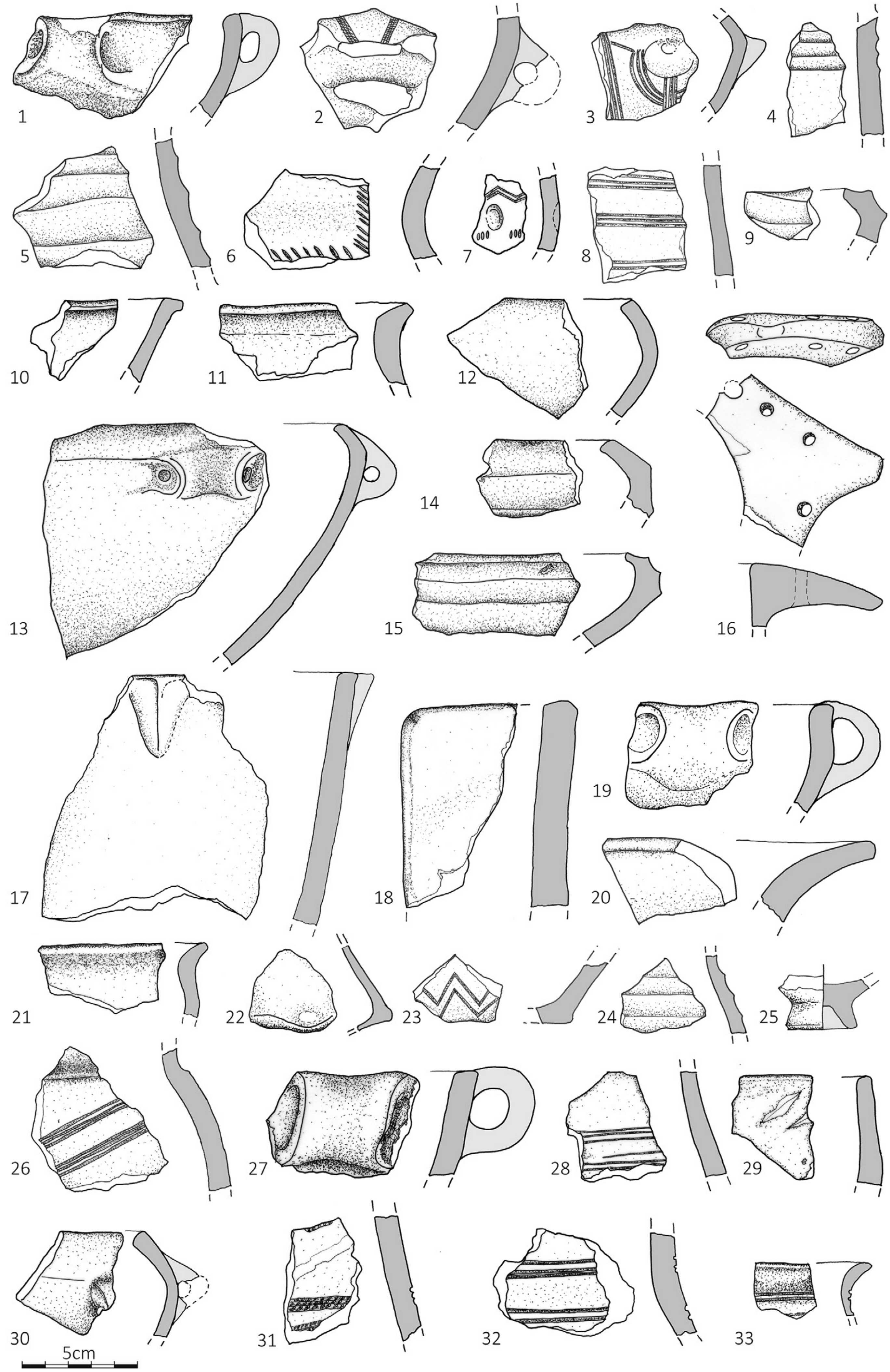

Sakule = 1-11; Crepaja = 12-16; Sefkerin = 17-26; Sefkerin 2 = 27-30; Kačarevo = 31-33

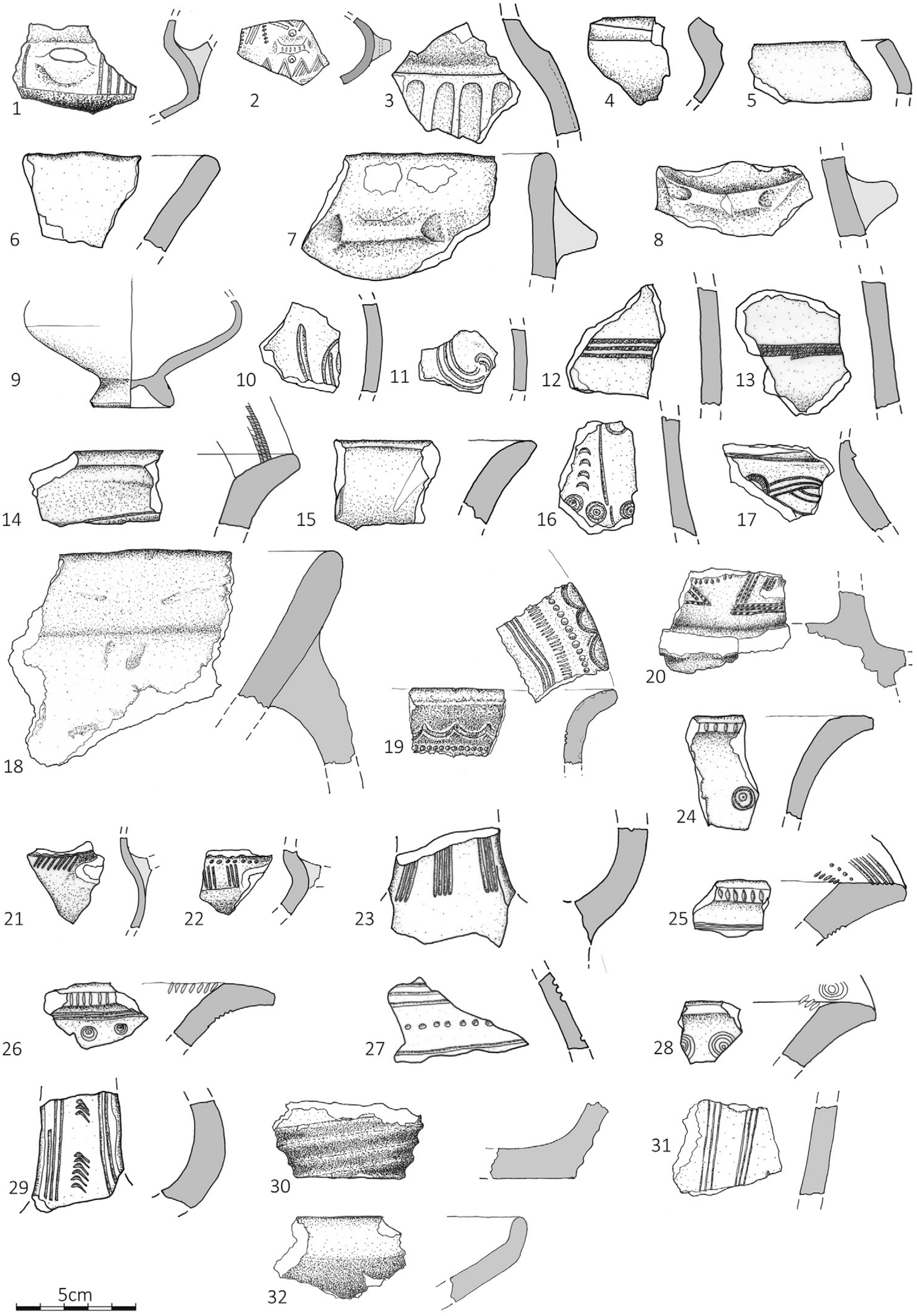

Glogonj 2 = 1-2; Jabuka = 3-5; Glogonj = 6-8; Kačarevo 2 = 9; Pančevo = 10-11; Mramorak = 12-16; Mramorak 2 = 17; Bavanište = 18-20; Bavanište 2 = 21-22; Bavanište 3 = 23-29; Jaša Tomić = 23-29
